# Evolutionary plasticity of the NHL domain underlies distinct solutions to RNA recognition

**DOI:** 10.1038/s41467-018-03920-7

**Published:** 2018-04-19

**Authors:** Pooja Kumari, Florian Aeschimann, Dimos Gaidatzis, Jeremy J. Keusch, Pritha Ghosh, Anca Neagu, Katarzyna Pachulska-Wieczorek, Janusz M. Bujnicki, Heinz Gut, Helge Großhans, Rafal Ciosk

**Affiliations:** 1 Friedrich Miescher Institute for Biomedical Research, Maulbeerstrasse 66, 4058 Basel, Switzerland; 2 Swiss Institute of Bioinformatics, 4058 Basel, Switzerland; 3 Laboratory of Bioinformatics and Protein Engineering, International Institute of Molecular and Cell Biology in Warsaw, ul. Ks. Trojdena 4, 02-109 Warsaw, Poland; 4 Institute of Bioorganic Chemistry, Polish Academy of Sciences, ul. Noskowskiego 12/14, 61-704 Poznan, Poland; 5 Faculty of Biology, Institute of Biotechnology and Moleular Biology, Adam Mickiewicz University, ul. Umultowska 89, 61-614 Poznan, Poland; 6 University of Basel, Petersplatz 1, 4001 Basel, Switzerland

## Abstract

RNA-binding proteins regulate all aspects of RNA metabolism. Their association with RNA is mediated by RNA-binding domains, of which many remain uncharacterized. A recently reported example is the NHL domain, found in prominent regulators of cellular plasticity like the *C. elegans* LIN-41. Here we employ an integrative approach to dissect the RNA specificity of LIN-41. Using computational analysis, structural biology, and in vivo studies in worms and human cells, we find that a positively charged pocket, specific to the NHL domain of LIN-41 and its homologs (collectively LIN41), recognizes a stem-loop RNA element, whose shape determines the binding specificity. Surprisingly, the mechanism of RNA recognition by LIN41 is drastically different from that of its more distant relative, the fly Brat. Our phylogenetic analysis suggests that this reflects a rapid evolution of the domain, presenting an interesting example of a conserved protein fold that acquired completely different solutions to RNA recognition.

## Abstract (web summary)

The *C. elegans* LIN-41 and its homologs, including human TRIM71/LIN41, contain the RNA binding NHL domain. Here the authors combine computational analysis, structural biology and in vivo studies, to explain how these proteins bind RNA and how rapid evolution of NHL domains resulted in different solutions to RNA recognition.

## Introduction

RNA-binding proteins (RBPs), by controlling different aspects of RNA metabolism, are central to gene expression. The TRIM-NHL family of RBPs includes key regulators of cell proliferation and differentiation^1–3^. Highlighting their importance, mutations in the human TRIM-NHL proteins have been associated with various diseases, including cancer and neurological disorders^4^. The term “TRIM” refers to a tripartite motif consisting of RING finger, B-box(es), and coiled-coil domains, and the “NHL” to the so-called NHL repeats, named after the proteins NCL-1, HT2A, and LIN-41^4,5^. Some TRIM-NHL proteins additionally contain a filamin domain between the TRIM and NHL domains^4^. The *Caenorhabditis elegans* TRIM-NHL protein LIN-41 (here referred to as CeLIN41) is widely known as a prototypic target of the *let-7* miRNA and a key factor in the heterochronic pathway, which regulates developmental transitions in the soma^6,7^. In addition, the germline-expressed CeLIN41 controls reprogramming into pluripotency during the oocyte to embryo transition^8^. Also the human TRIM71/LIN41 (HsLIN41) has been implicated in cellular plasticity, facilitating the reprogramming of differentiated cells into pluripotent cells^9^. In both worms and mammalian cells, LIN41 function has been connected to messenger RNA regulation. By associating with specific mRNAs, LIN41 targets them for either degradation or translational repression^10,11^. However, what determines the specificity of LIN41 toward its targets has remained unknown.

Different types of RNA-binding domains (RBDs), such as K homology, RNA recognition motif (RRM) or zinc finger among others, are used by RBPs to associate with specific RNA elements. Recent studies suggest that the currently understood types of RBP–RNA interactions are but the tip of an iceberg, implying the existence of many uncharacterized RBDs^12–14^. The NHL domain, which folds into a WD40-like β-propeller, is an example of a recently reported RNA-binding fold^10,15,16^. However, how exactly the NHL domain binds RNA remains largely unexplored. Thus far, the RNA-binding specificity has been explained only for the NHL domain of the *Drosophila melanogaster* protein Brat (DmBrat), which associates with single-stranded RNA (ssRNA) in a sequence-specific manner^15,17,18^. In contrast to this binding strategy, we find that LIN41 evolved a very different solution to RNA binding. By combining computational analysis with in vivo studies and crystallography, we find that a positively charged pocket, specific to the NHL domain of LIN41, recognizes a short stem-loop (SL) element, LRE (LIN41 response element), whose shape dictates the binding specificity. By analyzing predicted structure models of NHL domains from various proteins and species, we propose that the distinct RNA-binding mechanisms of LIN41 and Brat reflect a rapid evolution of the NHL domain.

## RESULTS

### Different RNA-binding preferences of TRIM-NHL proteins

**Fig. 1:**
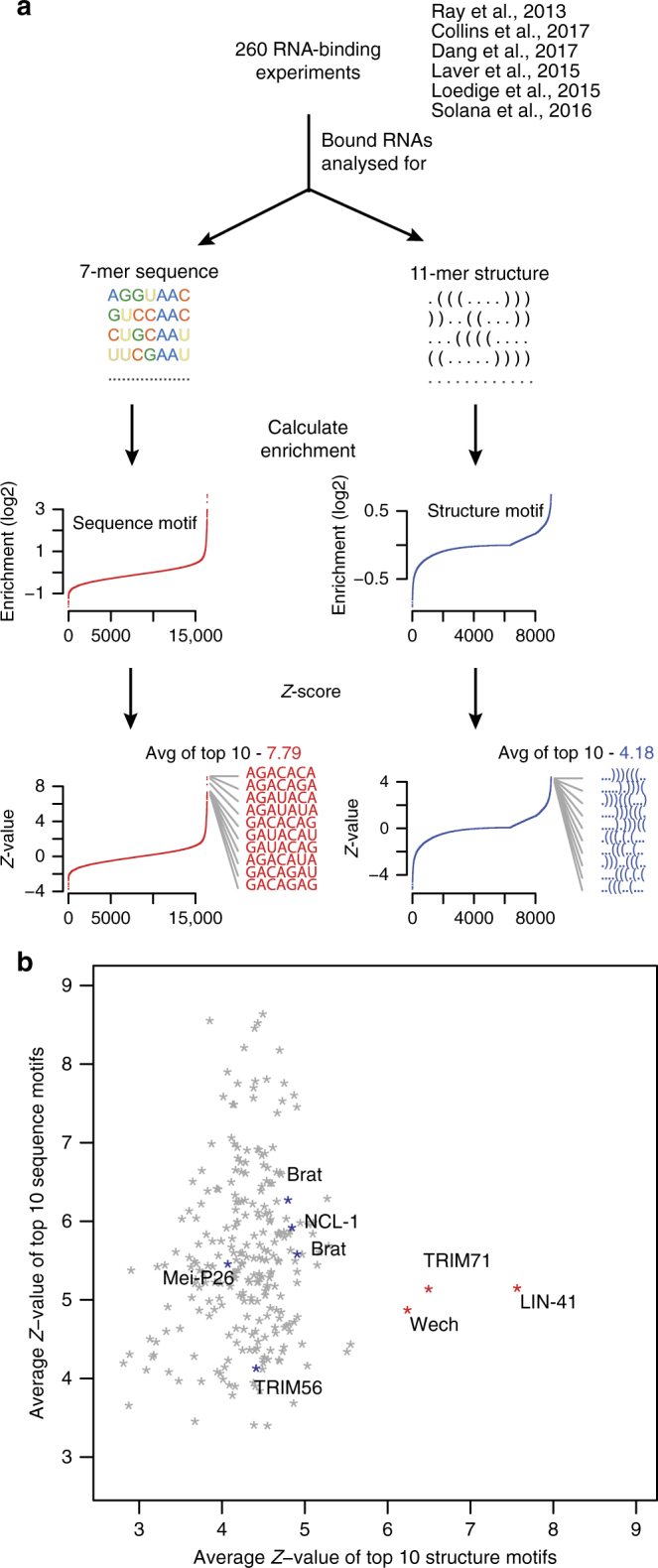
Structure vs. sequence preference of RNA-binding proteins. **a** Schematics describing the meta-analysis of 260 RNAcompete experiments. The top 2% of RNAs enriched in pulldowns were considered as bound fraction. All possible 7-mer sequence and 11-mer structure motifs were counted in the bound and unbound fractions to calculate enrichment of each motif. The enrichment values were then *Z*-value transformed and average *Z*-values of the top 10 motifs were calculated. The schematics shows these calculations for one of the 260 RNAcompete experiments as an example. **b** Average *Z*-values of the top 10 sequence motifs were plotted against the top 10 structure motifs, for each RNA-binding experiment, comparing preference for sequence vs. structure. The top three outliers with high structure preference, LIN-41, TRIM71, and Wech, all related TRIM-NHL proteins, are shown in red. Other TRIM-NHL proteins included in the meta-analysis are shown in blue (NCL-1, Brat, Mei-P26, TRIM56; Brat is marked twice, as it was analyzed in two separate studies)

Recent technological advances made characterization of RBP–RNA interactions possible on a massive scale. RNAcompete utilizes a single step in vitro binding reaction to determine preferences of RBPs/RBDs to short RNA sequences^19^. A large-scale RNAcompete study of 205 RBPs reported that the vast majority of RBPs bind to ssRNA^20^. Although a handful of those proteins showed statistically significant evidence for binding secondary structures, the structures were not absolutely required. Since then, additional studies using RNAcompete on diverse RBPs were published^17,18,21–23^, opening the possibility to revisit the question of preference of RBPs for RNA sequence versus structure. This experimental platform (GPL16119) uses RNA oligonucleotides ranging from 30 to 41 nucleotides, potentially allowing assessment of compact RNA structures. Similar to previous analyses, we quantified sequence preferences by calculating enrichments of sequence 7-mers (4^7^ = 16,384 combinations) in the RBP-bound fraction over the unbound fraction. To capture potential structure preferences in a comparable fashion, we in silico folded all RNA sequences by RNAfold^24^ and obtained RNA secondary structure in a dot-bracket format. In this format, a dot represents an unpaired nucleotide and a bracket a paired nucleotide. To roughly match the total number of combinations for sequence and structure *n*-mers, we set the length of the dot-bracket *n*-mers to 11, which resulted in a total of 9020 combinations. Henceforth, binding preferences for sequence 7-mers or structure 11-mers were calculated identically: for each RBP experiment, we asked whether there exists a small number of sequence 7-mers, or structure 11-mers, which show substantially higher binding enrichments than any other 7-mer or 11-mer, respectively (Fig. 1a). This approach ensured that only highly specific RBP–RNA associations were considered, avoiding bias for general features like GC content or overall tendency to form structured RNA. In Fig. 1b, we compared binding preferences for specific sequence motifs (*Y* axis) versus structured elements (*X* axis) for every RBP. In accordance with previous findings^20^, we found that the majority of RBPs displayed preference for sequence over structure. However, a handful of RBPs deviated drastically from that trend. Those RBPs were not part of the original large-scale study^20^, which may explain why they were not previously reported as having preference for secondary structure. The top three outliers (*C. elegans* LIN-41, human TRIM71, and *D. melanogaster* Wech; red asterisks in Fig. 1b) are all homologous TRIM-NHL proteins; no other homologs were analyzed by RNAcompete. For simplicity, we refer to these proteins as LIN41. Their preference for structure was striking, given that other members of the TRIM-NHL family, such as NCL-1, TRIM56, Brat, and Mei-P26, showed no such preference in the past and in our analysis (Fig. 1b, blue asterisks), suggesting a distinct RNA-binding mechanism for LIN41.

### LIN41 proteins repress mRNA via structured RNA elements

**Fig. 2:**
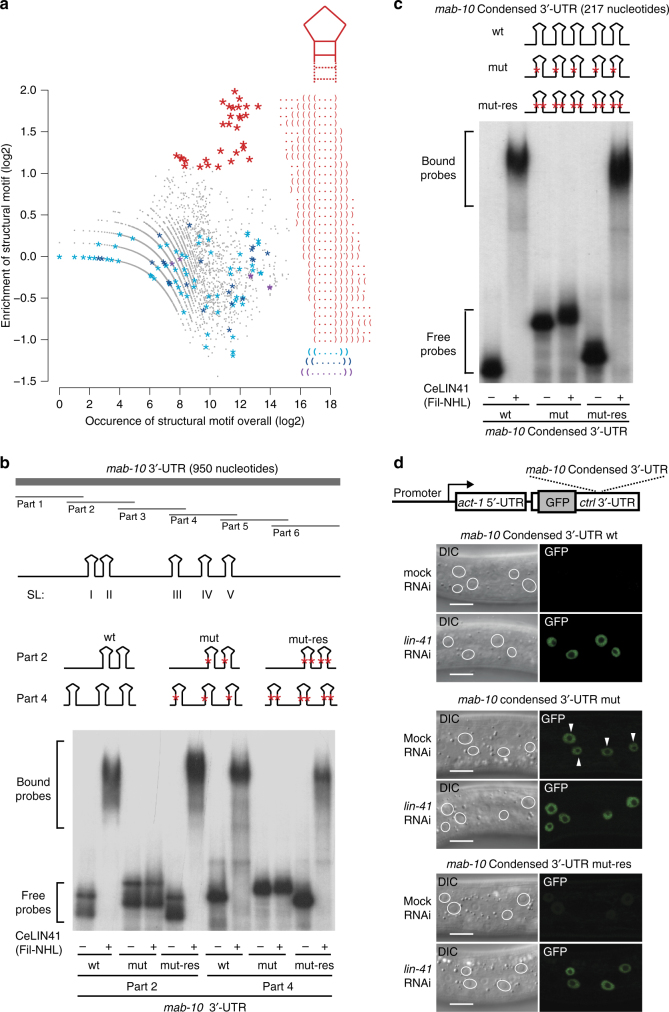
CeLIN41 recognizes structured RNA. **a** Enrichment for each 11-mer structure motif in the CeLIN41 RNAcompete experiment was plotted against its overall occurrence in the RNA pool. Top 30 enriched 11-mer structures are highlighted in red and the corresponding dot-bracket strings are indicated. The dot-bracket strings correspond to a stem-loop motif, with exactly three nucleotides in the loop, schematically represented above. As controls, all structure 11-mers containing a loop with four, five or six nucleotides, highlighted in cyan, blue and purple, did not show any enrichment. **b** CeLIN41 binding to parts of *mab-*10 3′-UTR. Top: schematics of the *mab-10* 3′-UTR divided into six overlapping RNA probes (part 1 to 6), each of 200 nucleotides. Tri-loop SLs are present in parts 2 and 4. Single red asterisk on one side of the stem indicates a stem disrupting mutation (mut) and two red asterisks, one on each side of the stem, indicate a stem disrupting and the compensatory stem-restoring mutations (mut-res). Respective sequences are listed in Supplementary Table 1. Bottom: in gel-shift experiments, CeLIN41 bound to parts 2 and 4 of *mab-10* 3′-UTR. Mutations disrupting the stems in part 2 and part 4 nearly abolished CeLIN41 binding. Compensatory mutations in the stems (mut-res) restored the binding to wild-type levels. **c** Top: the “wt” RNA corresponds to a synthetic “condensed” *mab-10* 3′-UTR reporter construct containing five stem-loops (I to V). Asterisks denote mutations as in **b**. Respective sequences are listed in Supplementary Table 1. Bottom: in gel-shift experiments, CeLIN41 bound the synthetic *mab-10* condensed 3′-UTR. Mutations disrupting the stem (mut) abolished binding, whereas compensatory mutations (mut-res) restored binding. **d** Micrographs of early L3-stage *C. elegans* larvae, treated with either *lin-41* or mock RNAi, showing reporter GFP in hypodermal nuclei (white circles demarcate nuclei), expressed from the *lin-29A* promoter under the control of unregulated *act-1* 5′-UTR and *unc-54* (ctrl) 3′-UTR. The 3′-UTR insert corresponds to the constructs in **c**. The *mab-10* condensed 3′-UTR imposed CeLIN41-mediated repression on the GFP reporter. Mutations disrupting the stem abolished this regulation (white arrowheads point to GFP-expressing nuclei), while compensatory mutations reinstated the repression. Scale bars, 10 µm

To determine the RNA secondary structure recognized by LIN41, we initially focused on the *C. elegans* protein. We re-normalized the RNAcompete data for CeLIN41 (see Methods) and calculated enrichments for structure 11-mers, as described in Fig. 1a. For each possible dot-bracket *n*-mer, we compared its overall occurrence in the RNA pool with its enrichment in the bound RNA fraction (Fig. 2a). A specific set of structure 11-mers stood out, showing enrichment values of up to four fold. Notably, all of these were variants of a small SL with three nucleotides in the loop (Fig. 2a; top 30 are shown in red). To test whether the requirement for three nucleotides in the loop was strict, we inspected all structure 11-mers with larger loops and detected no enrichment for such SLs (Fig. 2a). Thus, our analysis suggested that CeLIN41 recognizes SLs with exactly three nucleotides in the loop (tri-loop SLs).

CeLIN41 has recently been shown to directly bind mRNAs^10^. Among CeLIN41 mRNA targets are *mab-10*, *mab-3*, and *dmd-3*, which associate with CeLIN41 through their 3′-untranslated regions (UTRs), and *lin-29A*, which associates via the first exon of its 5′-UTR, for simplicity *lin-29A* 5′-UTR^10^. Using programs to predict RNA secondary structure, we observed a number of SLs with tri-loops in those UTRs (Supplementary Note 1 shows secondary structure models of *mab-10* and *lin-29A* UTRs). Interestingly, in the *mab-10* 3′-UTR, the tri-loop SLs were present in the regions previously shown to bind CeLIN41 in gel-shift experiments^10^ (parts 2, 3, and 4 in Fig. 2b). A majority of the SLs (9/13) in the *mab-10* 3′-UTR and *lin-29A* 5′-UTR had a “U–A” base pair adjacent to the loop (here the stem position 1), suggesting possible functional similarity. Hence, we tested those SLs as candidate CeLIN41 binding sites (Supplementary Note 1 and Supplementary Fig. 1a-b).

To evaluate their importance for CeLIN41 binding, we modified parts 2 and 4 of the *mab-10* 3′-UTR, and examined their association with a purified CeLIN41 fragment containing the filamin and NHL domains in gel-shift experiments. Specifically, we disrupted the candidate SLs by mutating a single G–C base pair in the stem and introduced compensatory mutations to restore the stem. Although the stem-disrupting mutations abolished the binding, the compensatory mutations (which reestablished the structure) restored the binding (Fig. 2b). We concluded that, in vitro, CeLIN41 recognizes tri-loop SLs. We then asked whether those SLs mediate CeLIN41 regulation in vivo. To answer this, we examined transgenic animals expressing green fluorescent protein (GFP) reporters (from either a hypodermal, *lin-29A*, or ubiquitous, *dpy-30*, promoter) under the control of different 5′ or 3′-UTRs. To test SLs in the 3′-UTR, we created a synthetic “condensed” *mab-10* 3′-UTR, packing closer the five candidate SLs by deleting the intervening sequences. This 3′-UTR variant was sufficient to bind CeLIN41 in vitro and the binding required the SLs (Fig. 2c). In animals, the reporter containing wild-type SLs was repressed by CeLIN41 (Fig. 2d). Consistent with the in vitro binding results, the stem-disrupting mutations alleviated repression and the compensatory stem mutations restored the repression (Fig. 2d). In addition to examining the *mab-10* 3′-UTR, we examined the *lin-29A* 5′-UTR, which contained four candidate CeLIN41-binding SLs (Supplementary Note 1 and Supplementary Fig. 1b). We mutated these four SLs, as for *mab-10*, and tested CeLIN41 binding. In vitro, the mutated RNA displayed reduced binding to CeLIN41 (Supplementary Fig. 1c). For testing the repression in *C. elegans*, we used a reporter line with stem-disrupting mutations in SLs I and III, as we were unable to obtain a line with mutations in all the SLs. Nevertheless, disrupting only two SLs was sufficient to alleviate CeLIN41-mediated repression of the reporter (Supplementary Fig. 1d); in vitro, the corresponding mutations reduced the size of CeLIN41-RNA complexes, suggesting the loss of CeLIN41 binding to some but not all the SLs (Supplementary Fig. 1c). As expected, the compensatory mutations in SLs I and III restored the binding in vitro and the repression in vivo (Supplementary Fig. 1c-d). Taken together, our analysis of *mab-10* 3′-UTR and *lin-29A* 5′-UTR suggested that CeLIN41 represses mRNA by binding specific SL elements.

**Fig. 3:**
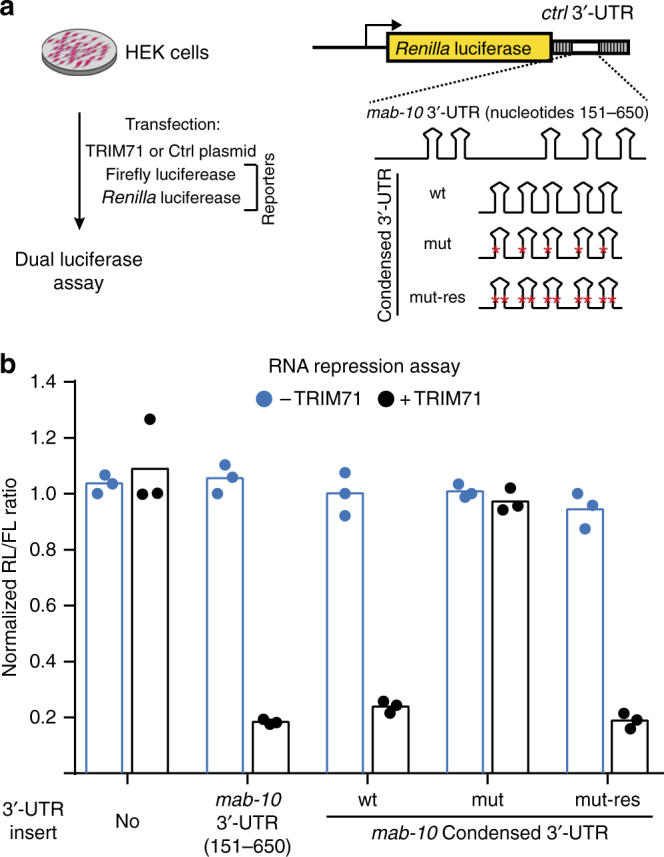
HsLIN41 recognizes *C. elegans* RNA stem-loops. **a** Schematics describing the luciferase assay and reporters used in this assay. 3′-UTR inserts transplanted into the RL (*Renilla* luciferase) construct are shown. **b** HsLIN41 specifically downregulated *Renilla* luciferase (RL) activity when sequences containing the SLs (from either *mab-10* 3′-UTR (151-650 nt) or *mab-10* condensed SL 3′-UTR) were transplanted into a control, unregulated 3′-UTR. The *mab-10* condensed 3′-UTR, with stem-disrupting mutations (mut), was not repressed by HsLIN41. Mutations that restored the SLs (mut-res) also restored the repression. Bars in the graph represent the mean between three biological replicates

Our computational analysis implied that, similar to CeLIN41, HsLIN41 (TRIM71) binds structured RNA (Fig. 1b). Therefore, we asked whether the *C. elegans* SLs could induce mRNA repression by HsLIN41 in human cells. To test this, we created reporter plasmids expressing, on one hand, *Renilla* luciferase (RL) under the control of *mab-10* 3′-UTR variants and, on the other hand, a control plasmid expressing firefly luciferase (FL) (Fig. 3a). Mammalian cells that either did, or did not, express HsLIN41 were co-transfected with reporter plasmids and the amount of luciferase-produced luminescence was measured to estimate HsLIN41-mediated repression. We found that HsLIN41 specifically repressed RL controlled by either the wild-type (nucleotides 151–650) or condensed *mab-10* 3′-UTR (Fig. 3b). This repression depended on the SLs, as it was alleviated by stem-disrupting mutations in the condensed *mab-10* 3′-UTR but restored by compensatory mutations (Fig. 3b). Thus, both *C. elegans* and human LIN41 appear to bind and regulate similarly structured RNA elements, suggesting a conserved mechanism of RNA recognition.

### LIN41 binds RNA stem loops

**Fig. 4:**
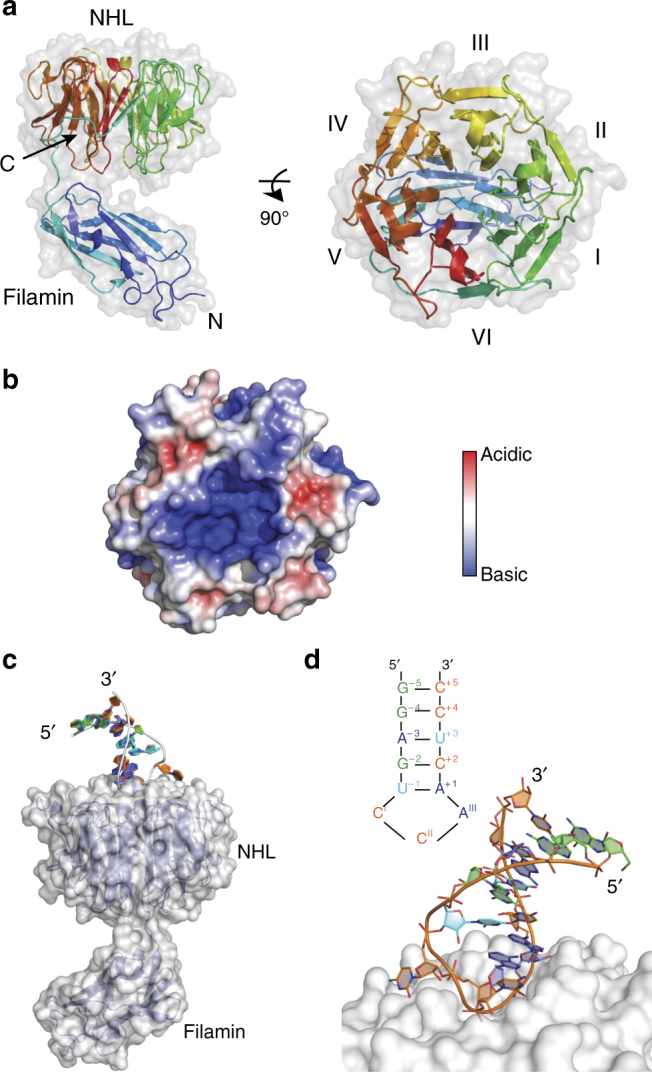
Crystal structure of the C-terminal part of *D. rerio* LIN41. **a** The crystal structure of the DrLIN41 filamin-NHL domains is displayed in a cartoon mode, with a transparent grey surface in two orientations rotated by 90°. The molecule is colored from blue (N terminus) to red (C terminus) to indicate the topology. Protein domains, termini, and β-propeller blades are labeled for better clarity. **b** Top view of the RNA-binding site of the DrLIN41 NHL domain with the electrostatic surface potential mapped onto the molecular surface. Surface potential is computed by using the APBS plugin implemented in PyMOL (www.pymol.org) and is displayed from – 5.0 kT/e (red, acidic) to + 5.0 kT/e (blue, basic). **c** DrLIN41 filamin-NHL domains in complex with the *lin-29A* stem-loop RNA. The filamin and NHL domains are shown in cartoon mode in blue, with a white transparent surface. The *lin-29A* RNA fragment, forming a hairpin, is displayed as a cartoon with nucleotides in different colors (guanine: green, adenine: blue, cytosine: orange, uracil: cyan). **d** Magnified view of the *lin-29A* RNA stem loop bound to the DrLIN41 NHL surface (colors as in **c**). A diagram detailing the nucleotide composition of the stem loop is shown above

To gain more insights into the binding mode of RNA by LIN41, we conducted crystallization trials with LIN41 proteins from several species. We obtained crystals for the *Danio rerio* (zebrafish) protein (DrLIN41), which bound the SL elements of *mab-10* 3′-UTR with similar specificity as the worm (CeLIN41) protein (Supplementary Fig. 2a and Fig. 2c). We then determined, at 2.6 Å resolution, the crystal structure of DrLIN41 protein in its unbound form, composed of the filamin and NHL domains (Uniprot E7FAM5, residues 435–824) (Fig. 4a). The protein crystallized in space group P2_1_2_1_2_1,_ with two molecules per asymmetric unit. Data collection and refinement statistics are summarized in Supplementary Table 2. Clear electron densities were present for both molecules, allowing model building of almost all residues. Only filamin-NHL inter-domain linker residues (538–548) were not resolved well, due to their flexibility. Therefore, Gly539 and Arg540 have not been included in one of the molecules (chain B) in the asymmetric unit. The intra-chain domain arrangement is equal in both molecules, whose structures are highly similar, with an overall root mean square deviation (r.m.s.d.) of 0.33 Å for Cα positions. The filamin domain is placed on the smaller side of the toroidal NHL domain, where it is located below the central axis and is tilted by ~ 70°. The two domains hardly contact each other and the weak electron density in the linker region points to a flexible domain arrangement. This makes it possible that the observed domain orientations originated from crystal packing constraints, which was also corroborated by analyzing the intra-chain filamin-NHL interface using the PISA^25^ and EPPIC^26^ software. As expected from sequence analysis, the NHL domain folds into a six-bladed β-propeller, where the first β-strand, following the filamin-NHL linker, complements the C-terminal sixth β-propeller blade as an outermost strand (Fig. 4a). By calculating the electrostatic surface potential, we observed that the cavity of the DrLIN41 NHL domain β-propeller opposite of the filamin domain (henceforth referred as the “upper” surface) features a large basic patch with a central cavity (Fig. 4b).

Next, to determine how LIN41 binds RNA SLs, we determined structures of the DrLIN41 protein in complex with two 13 nucleotide-long RNAs corresponding to SL-containing fragments of *lin-29A* 5′-UTR and *mab-10* 3′-UTR (at 1.90 Å and 2.35 Å resolution, respectively; data collection and refinement statistics are summarized in Supplementary Table 2). Both complexes crystallized in the same P3_1_21 space group, with one single protein–RNA complex in the asymmetric unit. The RNAs corresponding to the *lin-29A* and *mab-10* fragments form a stem loop and bind the cavity on the upper surface of the DrLIN41 NHL domain in a highly similar way, with an r.m.s.d of 0.43 Å (Fig. 4c-d and Supplementary Fig. 2b-c). The RNA stem starts with four Watson–Crick base pairs having the ribose sugar pucker in C3′-endo conformation, forming a regular A-form helix (positions 2 to 5 and – 2 to – 5). It continues with the + 1/– 1 base pair (both in C2′-endo conformation), leading into the trinucleotide loop, where the sugar rings assume C2′- and C3′-endo conformation for positions I/II and III, respectively. Only the tip of the SL interacts with the DrLIN41 NHL propeller, where the three unpaired nucleotides and the first two nucleotides on the 3′-side of the loop (positions + 1, + 2) bury together ~ 983 Å^2^ of solvent-accessible surface area. Superposition of the unbound structure and RNA complexes did not reveal major conformational changes in the NHL domain upon interaction with the 13-mer RNA, except for a few amino acid side chains that are directly involved in RNA recognition. This is not true for the filamin domain, which is rotated by ~ 90° along the immunoglobulin-fold axis and tilted by ~ 60° in the RNA complexes compared with the unbound structure, owing to the different crystal lattice contacts. This confirms the flexible nature of the filamin-NHL arrangement (Supplementary Fig. 2b). At position I of the RNA SL (C/U(I), C, or U in *lin-29A* and *mab-10* RNAs, respectively), the nucleotide is flipped out, due to the C2′-endo conformation of the preceding – 1 uracil moiety, leading to placement of this base onto the rim outside of the central RNA-binding cavity. Bases at loop positions II and III (C/U(II) and A/A(III) in *lin-29A* and *mab-10*, respectively), on the other hand, stack and complement the continuous base stacking of the helical stem at the 3′-side (Supplementary Fig. 2d-e). This seems critical for hairpin stability, as it enables the A(III) purine base to support the non-ideal A( + 1) – U(– 1) base pair, which is under considerable strain (Fig. 4d and Supplementary Fig. 2d-e). Its angle between base planes deviates by ~ 15° from planarity, which together with the propeller twist leads to an elongated hydrogen bonding distance between the A( + 1) N6 amino group and the U(– 1) O4 carbonyl (3.1 Å and 3.2 Å for *lin-29A* and *mab-10*, respectively). This is considerably longer than hydrogen bonds found in other base pairs of the hairpins, which have a standard length of 2.8–2.9 Å.

**Fig. 5:**
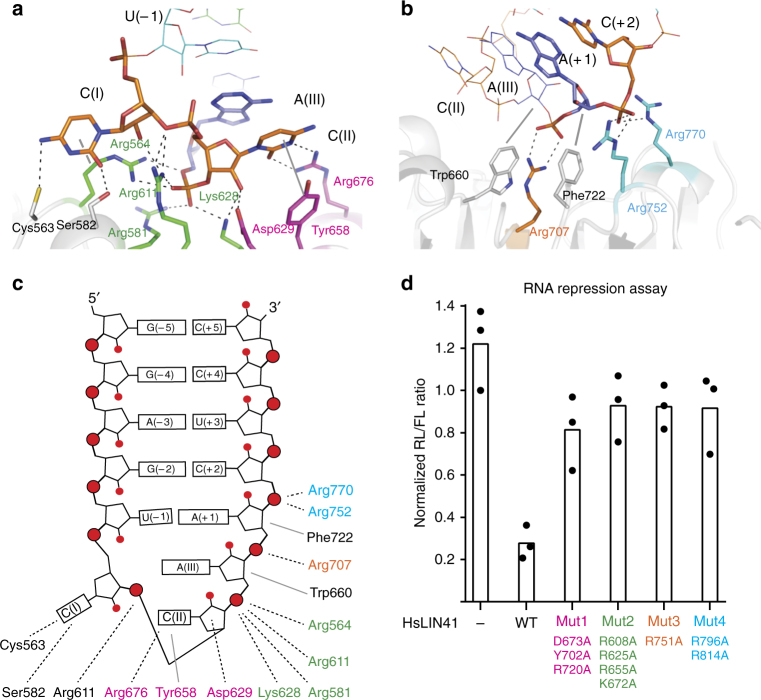
Molecular interactions underlying LIN41 binding to RNA SLs. **a**,** b** Detailed views of interactions between the *lin-29A* RNA and the DrLIN41 NHL propeller. Nucleotides and protein side chains are highlighted and their directly interacting residues are shown as sticks; the remaining parts are shown as lines (RNA) or ribbons (protein). Hydrogen bonds are presented as dotted lines and hydrophobic interactions as solid lines. Nucleotides are colored as in Fig. 4d, whereas protein side chains are colored according to the mutational analysis. **c** Schematic representation of the *lin-29A* RNA hairpin and of its interactions with DrLIN41 NHL residues (type of interaction and color code as in **a**, **b**). **d** Expression of mutant HsLIN41 proteins did not severely down-regulate *Renilla* luciferase (RL) reporter expression unlike the wild-type HsLIN41, when a fragment corresponding to the *mab-10* condensed 3′-UTR was transplanted into an unregulated 3′-UTR of the reporter construct. Bars in the graph represent the mean between three biological replicates

The basic NHL cavity interacts mainly with the sugar-phosphate backbone of the RNA SLs, predominantly via electrostatic interactions (Fig. 5a-b). The cavity’s shape, diameter, and the positioning and spacing of positively charged lysine and arginine residues, specifically recognizes the three-dimensional (3D) structural conformation of the negatively charged SL backbone. Arg564, Arg581, Arg611, and Lys628 interact with phosphate groups of C/U(II) and A(III), whereas Asp629 binds the ribose C2′ hydroxyl of C/U(II). The phenol ring of Tyr658 stacks on the C/U(II) pyrimidine base, which is also stabilized by a weak interaction with the guanidinium group of Arg676, which seems to have considerable rotational freedom, as reflected by weak electron density for two side-chain conformations. The flipped-out C/U(I) bases form hydrogen bonds with Cys563 and Ser582, and a hydrophobic interaction with the aliphatic part of the Arg564 side chain, whereas the C2′ ribose hydroxyl contacts Arg611 (Fig. 5a). On the 3′-side of the loop, the A(+ 1) phosphate oxygen atoms are anchored by interaction with Arg707, whose guanidinium group stacks between aromatic Trp660 and Phe722 side chains, which themselves are in van der Waals contact with ribose rings of A(III) and A(+ 1). Finally, Arg752 and Arg770 bind the C/U(+ 2) phosphate groups (Fig. 5b). These direct interactions between amino acids and bases or phosphate backbone are summarized in Fig. 5c.

Comparing the LIN41 NHL domains from different species, the majority of residues involved in RNA SL binding are identical (Supplementary Fig. 2f), indicating a potentially conserved mechanism of RNA binding. To test comprehensively the functional importance of identified amino-acid/RNA contacts, we expressed different variants of HsLIN41 in HEK293 cells and analyzed their ability to repress mRNA using the luciferase assay. By changing specific amino acid residues of the NHL domain to alanine, we examined the importance of four sets of NHL residues for RNA regulation. The first set of residues, Asp673(629), Tyr702(658), and Arg720(676), corresponding positions in DrLIN41 are in brackets, interact with the pyrimidine nucleotide in the center of the RNA loop (C/U(II)). The second set, Arg608(564), Arg625(581), Arg655(611), and Lys672(628), interact with C/U(II) and A(III). The third and fourth sets, Arg751(707) and Arg796(752)/Arg814(770), interact with A(+ 1) and C/U(+ 2), respectively. As expected from the structural analysis, changing any set of RNA-interacting residues, while mostly not reducing protein levels (Supplementary Figs. 2g and 8), alleviated LIN41-mediated mRNA repression (Fig. 5d). Consistently, specific point mutations in CeLIN41, affecting amino acids corresponding to some of the above residues (Arg959 and Arg1055; residues 676 and 770 in DrLIN41), were previously shown to be detrimental to CeLIN41 function (*lin-41* alleles mg181 and mg182^6^). Similarly, the Tyr941Ala mutation (658 in DrLIN41) failed to rescue the developmental defects in *lin-41* mutant worms^8^.

### The LIN41 response element

**Fig. 6:**
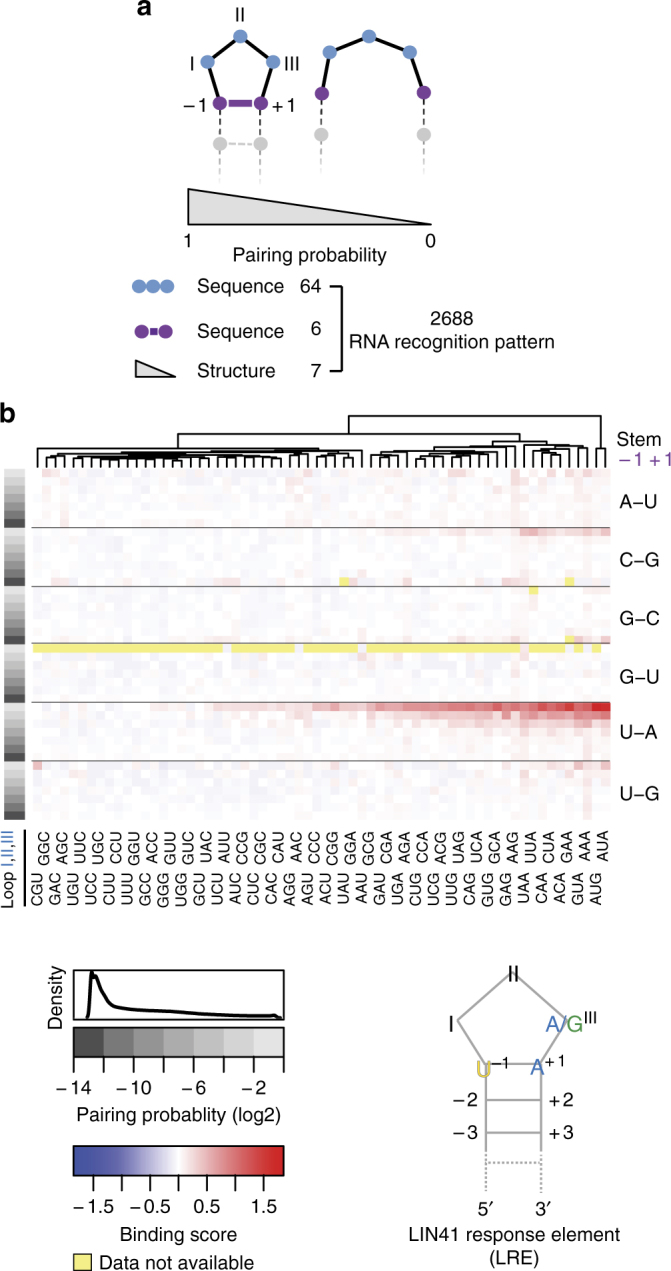
The LIN41 response element. **a** Schematics depicting RNA features used to build the LIN41 Response Element (LRE) model. Considered were all possible bases in the three loop positions (I, II, and III) and all possible base pairs at the stem position 1 (– 1/ + 1). The pairing probability of stem position 1 was determined by the relative occurrence of all possible structures that a particular RNA sequence can acquire. The pairing probabilities were grouped into seven bins on a log2 scale. Combining the sequence and structure features resulted in 2688 (6 × 64 × 7) RNA motif variants. **b** A heat map showing the average CeLIN41-binding scores from the RNAcompete experiment^17^ for all RNAs containing any particular motif variant as described in **a**. Pairing probability and base pairs at stem position 1 are shown on the left and the right of the heat map respectively. The loop (I, II, and III) sequences are shown in two rows, for clarity, at the bottom of the heat map. The data were clustered based on the CeLIN41-binding score. The overall distribution of pairing probabilities is shown on top of the pairing probability scale. Bottom right: the drawing represents a stem-loop motif, based on the model, referred to as the LIN41 Response Element (LRE). Yellow: data not available (RNA motif variants supported by < 20 oligo sequences)

To determine key characteristics of the RNA SLs that facilitate the binding to LIN41, we developed a computational model, which interrogated both RNA sequence and secondary structure. Given the importance of the loop (positions I, II, and III) and the two adjacent positions in the stem (positions – 1 and + 1), revealed by the crystal structure, we decided to examine all possible variations in these positions; all combinations of nucleotides in the positions I, II, and III (64), and all possible base-pairing combinations of nucleotides in the positions – 1 and + 1 (6), leading to a total of 384 RNA variants (Fig. 6a).

Obtaining secondary structure preferences posed a greater challenge, due to the involvement of a large number of residues, including those in positions – 1 and + 1 but also those further down the stem. However, the RNA/protein complex structure showed that LIN41 mostly interacts with the loop and the – 1/ + 1 residues of the stem, arguing that the main function of the stem is to present the 3-mer loop. Given that the pairing between the nucleotides – 1 and + 1 is required for the formation of a 3-mer loop, the tendency to form such a SL can be expressed by a pairing probability between the nucleotides – 1 and + 1. Therefore, using RNAfold^24^, we calculated pairing probabilities and grouped them into seven bins (log2 scale). We then combined structural and sequence features into a total of 64 × 6 × 7 = 2688 unique RNA recognition patterns (Fig. 6a). Using the in vitro binding data from RNAcompete^17^, we quantified the contribution of each of those RNA patterns to CeLIN41 binding, by calculating the average enrichment of all RNAs that contain a particular RNA pattern (see Methods). This revealed that the residues in the – 1/ + 1 stem positions, the pairing probability, as well as the 3-mer loop, were all critical for binding (Fig. 6b). The U–A (– 1/ + 1) pairing had by far the strongest impact on the binding, followed by C–G pairing with a much weaker impact. All other – 1/ + 1 base pairs showed no contribution to the binding. In the case of U–A, the extent of binding scaled with the pairing probability, indicating that our approach is able to capture the formation of SLs quantitatively. Among the 64 3-mer loop sequences, about one-half showed evidence for the binding, with a preferred purine base in position III (Fig. 6b). This is consistent with the structural requirement of a purine base at the position III to support the non-ideal constrained U–A base pair at stem position 1. Importantly, the binding determinants showed interdependence, e.g., G–C at position – 1/ + 1 could not be compensated by any 3-mer in the loop, nor by a high pairing probability. The same holds true for a very low pairing probability and low scoring 3-mer loop sequences, arguing that the high parameter model used here captures the subtleties of CeLIN41 binding. Summarizing, two RNA features, the U–A pair in positions – 1/ + 1 and A/G in position III of the loop, appear to be the key features of the LRE (Fig. 6b). Similar conclusions were reached by modeling binding specificity of DmWech and HsLIN41 (Supplementary Fig. 3a-b), suggesting that these proteins bind similar RNA elements.

**Fig. 7:**
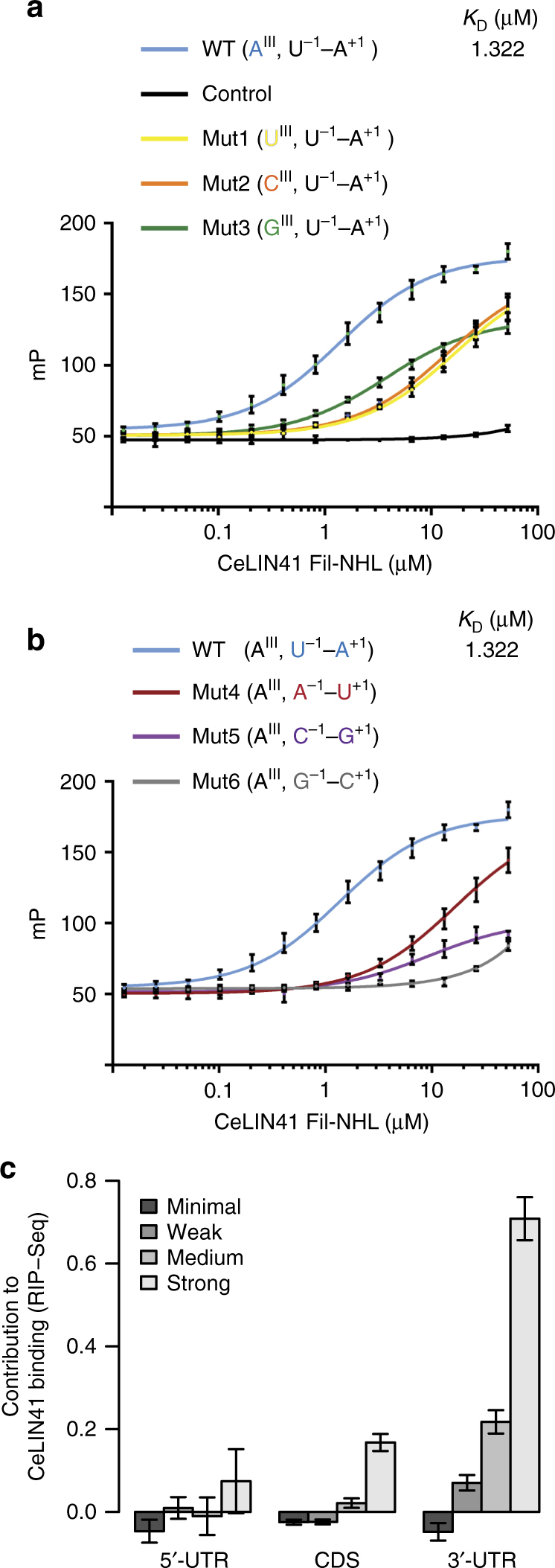
LIN41 binds to LREs both in vitro and in vivo. **a** Fluorescence polarization (FP) assays determining binding constants of CeLIN41 to LRE variants in the position III of the loop. Raw FP data of CeLIN41, interacting with a wild-type LRE (SL I in Supplementary Fig.1b), a control stem-loop RNA with five nucleotides in the loop, and LREs mutated at loop position III, are shown in units of millipolarization (mP). The equilibrium dissociation constant (*K*_D_) is shown for the WT LRE. Each data point is a mean of three experiments and the error bars represent the standard deviation. **b** The FP assays determining binding constants of CeLIN41 to LRE variants in the position 1 of the stem. Raw FP data of CeLIN41, interacting with WT LRE and LREs mutated at stem position 1, are shown in units of millipolarization (mP). Each data point is a mean of three experiments and the error bars represent the standard deviation. The WT LRE data is the same as in **a**. It is replotted for easy comparison with the mutants. **c** Contribution of LREs of varying strengths, predicted by the model in Fig. 6b, present in 5′-UTRs, coding sequences (CDS) and 3′-UTRs, to CeLIN41 binding as determined by linear regression. RNA binding was assayed by co-precipitation with CeLIN41, followed by RNA sequencing (RIP-seq). The error bars represent SEs for the coefficients obtained from the linear regression

To test the LRE model, we examined, by measuring fluorescence polarization, CeLIN41 binding to a 13-mer RNA folding into a single LRE (SL I in Supplementary Fig. 1b). The “wild-type” LRE (A^III^, U^−1^–A^+1^) bound to CeLIN41 with a dissociation constant (*K*_d_) of 1.322 µM, whereas a control 13-mer SL with five nucleotides in the loop did not show any detectable binding (Fig. 7a-b). Mutating residue III in the loop, from A to G, C, or U reduced the affinity, with G having the least effect (Fig. 7a). Similarly, mutating – 1/ + 1 residues from U–A to G–C, A–U, or C–G resulted in reduced complex formation, with G–C having the most severe effect (Fig. 7b). Taken together, the results of the binding assays suggest that the LRE model correctly recapitulates the SL determinants of CeLIN41 binding. As we obtained the crystal structure of the protein–RNA complex using the zebrafish LIN41 protein, we also tested the binding of LRE and its mutant variants to DrLIN41 and found it to be very similar to CeLIN41 (Supplementary Fig. 3c).

Regulatory RBPs typically associate with the 3′-UTRs of mRNAs. However, in addition to binding 3′-UTRs, CeLIN41 associates with at least one mRNA target via its 5′-UTR^10^. Using the LRE model, we were able to examine the general distribution of CeLIN41 binding across all mRNAs. Initially, we immunoprecipitated FLAG and GFP-tagged LIN41 together with associated RNAs from young adult worms (RNA immunoprecipitation, RIP). The RNAs were examined by RNA-seq (RIP-seq) and enrichment for all transcripts was calculated (Supplementary Fig. 4). Using the LRE model, we scanned the *C. elegans* transcriptome to predict LIN41-binding sites genome wide (see Methods). This resulted in a total of 177,179 predicted binding sites in 5′-UTRs (*n* = 6888), CDS (*n* = 153,837), and 3′-UTR (*n* = 16,454) with varying strengths (Supplementary Table 3). We then examined whether transcripts with the predicted LREs also showed binding to CeLIN41 (by RIP), taking into account the position of the binding sites in 5′-UTRs, CDS, and 3′-UTRs, as well as the predicted binding strengths. These features were then used as predictors in a linear regression, considering the RIP enrichment as a response (Fig. 7c). First, the analysis revealed that the association with CeLIN41 depended on the LRE-binding strength: the scaling strongly suggested that the LRE model is relevant for CeLIN41 binding in vivo, despite an overall low predictive power of the model (*r* = 0.158). Second, the predicted binding sites in the 3′-UTR contributed the most to CeLIN41 binding, suggesting that CeLIN41 regulates the majority of its targets via their 3′-UTRs.

### Evolutionary plasticity of RNA binding via the NHL domain

**Fig. 8:**
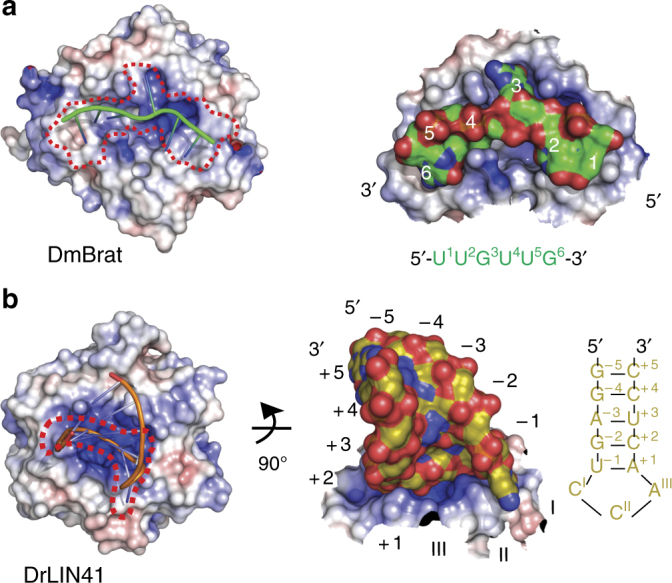
RNA binding preferences of LIN41 and Brat. **a** Left: crystal structure of the DmBrat NHL domain in a complex with a single-stranded linear RNA (PDB 4ZLR^17^). The protein surface is colored by the electrostatic surface potential from – 8 kT/e (red, acidic) to + 8 kT/e (blue, basic) and the RNA is shown as a cartoon. The approximate footprint of the RNA interaction on the protein surface is shown as a dotted red line. Right: magnified view of the RNA-binding site. A fragment of the interacting protein surface is shown and colored as on the left. The RNA is shown in surface mode, with carbon atoms in green and other atoms in standard colors. Nucleotide positions are labeled as in the RNA sequence displayed below. **b** Left: crystal structure of DrLIN41 in complex with the *lin-29A* RNA stem loop. The protein surface is colored as in **a**. Right: magnified view of the RNA-binding site in an orientation rotated by 90°. The RNA stem loop is shown in surface mode, with carbon atoms in gold and other atoms in standard colors. The corresponding RNA sequence is on the right

Structurally, as determined in a DALI search^27^, the DrLIN41 NHL domain is most similar to the NHL domain of DmBrat, with an overall r.m.s.d. of 1.9–2.1 Å, for PDB entries 1Q7F, 4ZLR, and 5EX7, respectively^17,28^. However, the upper (RNA-binding) surfaces of these NHL domains are structurally very different (Fig. 8a-b) and key RNA-binding residues are not conserved^17^. The DmBrat NHL domain binds a ssRNA consensus motif, 5′-UUGUUG-3′, and serves as a prototype for sequence-specific recognition of a short linear RNA motif by an NHL domain (PDB 4ZLR^17^). The key protein determinant of this mode of interaction is a mixed hydrophobic and positively charged surface of high structural complexity, which specifically accommodates individual bases, as well as increases binding energy via electrostatic interactions with at least some parts of the RNA sugar phosphate backbone. The surface of the DmBrat NHL domain features cavities and channels, which “scan” individual bases for sequence specificity. The three bases on the 3′-side of the consensus motif reside in a deep channel and position the fourth flipped out G(3) base in another cleft (Fig. 8a). By contrast, the NHL domain of DrLIN41, although using the same NHL propeller fold, has a very different surface, which evolved to accommodate a specific conformation of a trinucleotide RNA stem loop. It features a highly positively charged, shallow, central cavity, which is surrounded by walls on all sides. Owing to a very compact structure of the SL (only the base at position I is flipped out, whereas all other bases are part of a continuous stack), the specific conformation of the sugar-phosphate backbone is used to achieve specificity, whereas the negative charge of the phosphate groups provide most of the binding energy (Fig. 8b).

**Fig. 9:**
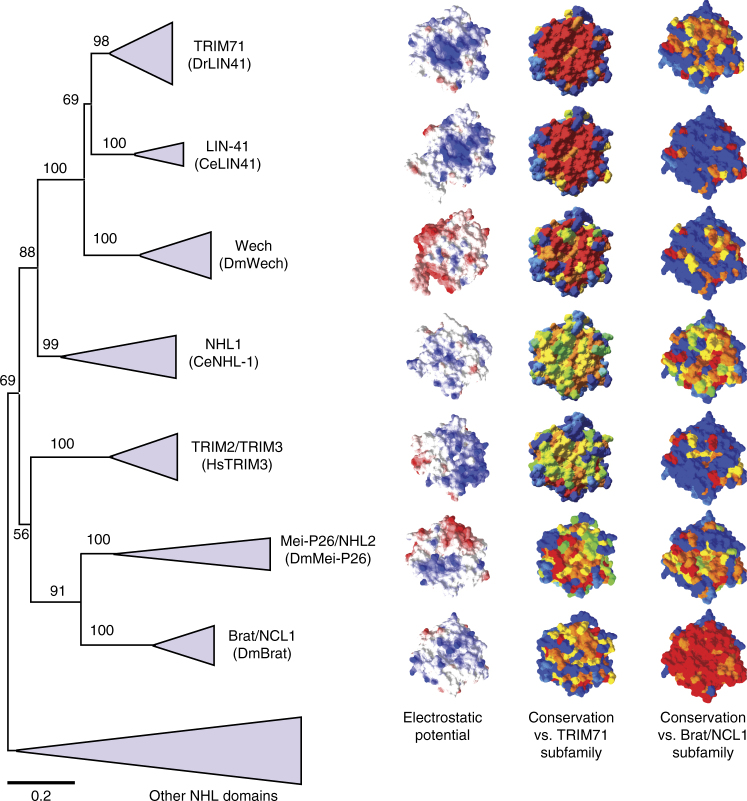
Evolutionary relationships between NHL domains. Left: a simplified phylogenetic tree of NHL domains, with a focus on putative RNA-binding proteins. For details, see Supplementary Fig. 5. Branches comprising multiple sequences from one subfamily (named after a representative protein) have been collapsed and are illustrated as triangles. The apex of a triangle indicates the start-point for an individual branch, whereas the width of the base indicates the number of the members that constitute the branch. The tree is drawn to scale, with branch lengths in the same units as evolutionary distances used to build the phylogenetic tree. Bootstrap values are shown for all nodes. Right: structural characteristics of the selected representatives of the seven subfamilies; from top to bottom: DrLIN41, CeLIN41, DmWech, CeNHL-1, HsTRIM3, DmMei-P26, and DmBrat. All structures are shown in surface representation. The left column depicts either experimentally determined crystal structures (DrLIN41 and DmBrat), or computationally modeled 3D structures of the proteins, with the mapped electrostatic potential on the solvent accessible surface from – 5 kT/e (red, acidic) to + 5 kT/e (blue, basic). The middle column depicts the sequence conservation within the TRIM71 subfamily (top image) and between the TRIM71 subfamily and each of the other subfamilies, mapped on the DrLIN41 crystal structure. The right column depicts the sequence conservation within the Brat subfamily (bottom image) and between the Brat subfamily and each of the other subfamilies, mapped on the DmBrat crystal structure. The middle and right panel use the same coloring scheme: red for invariant residues (within the reference subfamily, i.e., TRIM71 or Brat, respectively, or between the reference family and the compared subfamily), orange and yellow for partially conserved residues, green for weakly conserved residues, and blue for non-conserved residues

To address the etiology of these two very different mechanisms of RNA binding, we performed a phylogenetic analysis of the NHL family (Supplementary Fig. 5 and Note 2). We found that TRIM71, LIN-41, Wech, NHL1, TRIM2/TRIM3, Mei-P26/NHL2, and Brat/NCL1 subfamilies belong to one branch of the NHL family (Fig. 9). Individual members of these subfamilies are shown in Supplementary Fig. 5. Each of these subfamilies forms a distinct group, strongly supported by high bootstrap values. In addition, high bootstrap values imply close phylogenetic relationship between Brat/NCL1 and Mei-P26/NHL2 subfamilies, and between TRIM71, LIN-41, and Wech subfamilies. The exact position of NHL1 and TRIM2/TRIM3 subfamilies is uncertain. However, they show stronger association with the LIN41-related group and the Brat-related group, respectively, rather than the other way around (Fig. 9). Our analysis of the existing/predicted NHL structures from proteins representing the individual subfamilies (DrLIN41, CeLIN41, DmWech, CeNHL-1, HsTRIM3, DmMei-P26, and DmBrat) revealed several differences, despite an overall similarity in their shapes. For example, the RNA-binding surface of the NHL domains of DmWech and DmMei-P26 displays large patches of negatively charged residues. In addition, despite relatively close relationship between the Brat/NCL1 and Mei-P26/NHL2 subfamilies, their NHL domains differ significantly in the distribution of electrostatic charge (Fig. 9, electrostatic potential).

Consistent with the variation in the charge, mapping sequence conservation, in relation to the reference DrLIN41 and DmBrat, revealed a lot of variation (Fig. 9, conservation). Even the relatively closely related Mei-P26/NHL2 and Brat/NCL1 subfamilies displayed a somewhat surprising lack of conservation of residues important for the RNA-binding by DmBrat. Given the paralogous character of relationship between these subfamilies (due to gene duplication), it is likely to be that they have different functions. Furthermore, the TRIM2/TRIM3 and NHL1 subfamilies displayed no significant conservation to either DrLIN41 or DmBrat RNA-binding surfaces, offering no clues to the mechanism of their potential association with RNA. The only two subfamilies that displayed high mutual conservation of the RNA-binding surface were TRIM71 and LIN-41. They shared both the shape and distribution of electrostatic potential, consistent with binding the same structured RNA element. The more remotely related Wech subfamily has diverged with respect to TRIM71 and LIN-41 subfamilies. Nevertheless, the partial conservation of the binding site (Supplementary Fig. 6) suggests that Wech family members bind RNA in a manner similar to TRIM71/LIN41.

## Discussion

Here, employing an integrative approach encompassing computational analysis, structural biology, and in vitro and in vivo studies, we dissected the RNA-binding mechanism of LIN41 proteins. The RNA-binding specificity of LIN41 results from shape-complementarity between a tri-loop LRE and a shallow pocket in the NHL domain. Due to the lack of extensive stacking interactions between bases and amino acids, LIN41 binds to a single LRE with relatively low affinity. One solution to achieve a higher binding strength and, consequently, tighter regulation in the cell may be the concentration of multiple LREs within UTRs of mRNA targets. For instance, the 5′-UTR of *lin-29A* contains four LREs and disrupting two of them was sufficient to alleviate CeLIN41-mediated repression in vivo. Moreover, the coiled-coil and B-box domains can mediate oligomerization of TRIM-NHL proteins, which has been shown to be critical for the E3 ligase activity of TRIM25 and TRIM32^29^. Therefore, it is possible that LIN41 and related proteins form a multimeric complex that binds to several LREs for more effective mRNA regulation.

LIN41 proteins have emerged as important regulators of proliferation vs. differentiation decisions in progenitor cells. In the *C. elegans* soma, in addition to *lin-29A* and *mab-10* studied here, CeLIN41 regulates *mab-3* and *dmd-3*^10^. By scanning their 3′-UTRs, we noticed that they too contain putative LREs, suggesting a common mechanism for CeLIN41 recruitment to these mRNAs. Interestingly, CeLIN41 binding to the 5′-UTR (*lin-29A*) was shown to induce translational repression, whereas the binding to the 3′-UTR (*mab-10*) results in mRNA decay^10^. As in both cases the recruitment of CeLIN41 is mediated by LREs, the mRNA regulation is presumably dependent on co-factors recruited to RNA by CeLIN41. The same may apply to HsLIN41, which, when artificially tethered to reporter RNAs, mediates both translational repression and mRNA degradation^11^. In addition to the somatic function, CeLIN41, and particularly its NHL domain, is also critical in the germline, where putative targets remain to be evaluated^8,30–32^. For example, the regulation of one putative target, *cdc-25.3* mRNA, depends on both CeLIN41 and other RBPs, OMA-1, and OMA-2^30^. However, whether this regulation is direct and mediated via LREs remains to be tested.

Vertebrate LIN41 proteins are essential for different aspects of development, e.g., pluripotency, limb development, and neural tube closure^9,33,34^. However, unlike CeLIN41^8^, HsLIN41 has E3 ubiquitin ligase activity, regulating protein turnover^35–37^. Thus, which HsLIN41 functions are related to mRNA binding remains to be seen, and the LRE model is expected to help evaluating it. Although the model is not sufficiently predictive to determine which mRNAs are LIN41 targets, our data indicate that the targets tend to contain LREs. Thus, scanning candidate mRNAs for LREs should help in selecting best candidates for functional validation. The same applies to DmWech, which functions in muscles, particularly in the regulation of integrin-cytoskeleton connection^38^. Our finding suggest that at least some of these functions may involve mRNA regulation. Finally, although we focus here on mRNAs, it is possible that LRE-like SLs mediate the association of LIN41 also with other types of RNAs. For example, a recent study of proteins involved in miRNA processing showed that HsLIN41 specifically binds the apical loop of specific miRNA precursors, such as pre-miR29a and pri-miR-1-2^39^. Interestingly, when we used structure prediction programs to examine possible secondary structures of these two pre-miRNAs, we noticed that their apical loops could fold into an LRE-like SL (our unpublished observation), potentially explaining why these specific miRNAs, but not others, are regulated by HsLIN41.

The mechanism of RNA recognition by LIN41 is very different from that of DmBrat, cautioning against generalizations about RNA-binding modes derived from few studies. The RRM is another example of a protein fold that can recognize RNA sequence or structure; it binds predominantly ssRNA but, in some proteins such as U1A, RBMY, and TAF15, it recognizes RNA SLs^40–42^. Our phylogenic analysis of proteins carrying the NHL domain suggests that the different RNA-binding strategies of LIN41 and DmBrat reflect a rapid evolution of the RNA-binding surface of the domain. One example is DmWech, whose NHL domain, despite presumably binding LREs, displays a quite different distribution of the electrostatic potential. The NHL domains of other related proteins discussed here have diverged much further, to the point where predicting their RNA-binding preference (sequence versus structure) becomes highly speculative. Thus, whether other proteins containing NHL domains bind RNA, and whether their binding mechanisms are reminiscent of Brat-like recognition of ssRNA or LIN41-like recognition of SLs, are exciting questions for future research.

## Methods

### Experimental model

Supplementary Table 4 lists the *C. elegans* strains used in this study. To obtain synchronous worm populations, embryos were extracted from gravid adults with a bleaching solution (30% (v/v) sodium hypochlorite (5% chlorine) reagent (Thermo Fisher Scientific; 419550010), 750 mM KOH), and were incubated overnight in the absence of food at room temperature in M9 buffer (42 mM Na_2_HPO_4_, 22 mM KH_2_PO_4_, 86 mM NaCl, 1 mM MgSO_4_). Hatched, arrested L1 larvae were plated on food and incubated at 25 °C for the desired number of hours. For RNA interference (RNAi) experiments, arrested L1 larvae were plated on RNAi-inducing NGM agar plates containing *Escherichia coli* HT115 bacteria carrying plasmids targeting genes of interest^43^.

### Calculating sequence and structure preferences of RBPs

We downloaded publicly available data from six studies (PMID:23846655, PMID:26527002, PMID:27502555, PMID:25962635, PMID:28379442, PMID:28697339) that used the RNAcomplete assay based on the platform GPL16119 (www.ncbi.nlm.nih.gov/geo/). The respective accessions were GSE41235, GSE73000, GSE75554, GSE60498, GSE96990, and GSE93949. We downloaded the data for GSE41235 from hugheslab.ccbr.utoronto.ca/supplementary-data/RNAcompete_eukarya/ and the rest from GEO (Series Matrix File). All the data were then combined into a single table containing the binding scores from the original authors. As a precautionary measure, we then removed 11.9% of the 241,357 oligonucleotides that showed a median intensity lower than 6 (log2 space) over the full set of 244 experiments (hugheslab.ccbr.utoronto.ca/supplementary-data/RNAcompete_eukarya/raw_data.txt.gz), arguing that those are probably less reliable (see also Supplementary Fig. 7a). For every experiment, we specified the bound fraction by selecting the top 2% of the RNAs with the highest binding score. To quantify sequence preferences for each RNAcompete experiment, we counted the number of occurrences of all 7-mer sequences in the bound fraction (foreground) and compared that with a size-matched background obtained from counting the number of occurrences in all the RNAs divided by 1/0.02 = 50. We then added a pseudo-count of 8 (to avoid high enrichment values caused by low count numbers), log2 transformed the data, and subtracted the background from the foreground to calculate log2 enrichment values for each 7-mer. We then wanted to determine if there were outliers in that distribution which would suggest that the profiled RBP prefers a particular 7-mer over all the others. For this, we converted the enrichments to *Z*-scores and calculated the average *Z*-score of the top ten 7-mers. RBPs with a high specificity for a small set of 7-mers should thus produce high top ten *Z*-scores. To quantify structure features, we performed the same procedure as above, replacing the RNA sequence strings by dot-bracket strings obtained from in-silico folding all the RNAs with RNAfold (2.1.5)^24^. In this case, we used a motif length of 11 to roughly match the number of structure combinations (*n* = 9020) to the number of 7-mer sequence combinations (*n* = 16,384). Theoretically, there are 3^11^ = 177,147 dot-bracket strings of length 11 but most of those do not represent valid secondary structures.

### Preprocessing and normalization of the RNAcompete data

We downloaded the raw data for LIN-41, TRIM71, and Wech from GEO (GSM1876411, GSM1876409, GSM1876410), and extracted the raw intensity values from the respective channels on the two color arrays. Unlike in most setups using two color array, where samples of interest and control samples are run on the same array, in the RNAcompete setup each channel contained a different RNAcompete experiment. The original authors calculated enrichment values for a particular RBP by comparing a particular sample with a compendium of other RBPs^20^. We thus downloaded a large compendium of such experiments (hugheslab.ccbr.utoronto.ca/supplementary-data/RNAcompete_eukarya/raw_data.txt.gz) and created an input sample by calculating the median intensity for every oligo on the array. When comparing this input with LIN-41, TRIM71, and Wech (after quantile normalization), we noticed sample-specific shifts for oligonucleotides with very low intensity levels likely to be caused by technical limitations: in the case of LIN-41, oligonucleotides with a low intensity in the input showed no substantial enrichment in the pulldown. This could be explained by low efficiency oligonucleotides that do not measure the intended target but cross hybridize to produce some low level of intensity. We thus removed those oligonucleotides using an intensity cutoff (Supplementary Fig. 7a). In addition, we found oligonucleotides in this experiment that were depleted in the input and formed a small subpopulation. As it is unlikely for an RBP to specifically deplete a small subset of RNAs, we removed such unreliable oligonucleotides (Supplementary Fig. 7a). Unlike in the case of LIN-41, for TRIM71 and particularly so for Wech, we noticed a large fraction of oligonucleotides that were of very low intensity in the input but showed substantial enrichment in the pull-down, suggesting that this was likely a technical artifact. We thus conservatively removed those oligonucleotides (Supplementary Fig. 7a). We calculated binding enrichment values (log2) for the retained oligonucleotides by performing a loess fit between the input and the respective pulldown and calculating the vertical distance between a given oligonucleotide and the fit (Supplementary Fig. 7a).

### RNA-binding model for LIN41 proteins

Based on the observation that LIN41 binds an RNA stem loop with a loop length of exactly three nucleotides, we devised a model to characterize the detailed binding specificity. We incorporated two sequence features, namely the loop region (positions I, II, III) and the two nucleotides surrounding the loop (positions – 1 and 1). To capture the tendency of a given RNA to form a SL in the thermodynamic ensemble, we calculated pairing probabilities between positions – 1 and 1 based on RNAfold (2.1.5)^24^ using the option -p to calculate the partition function. From the resulting base pairing matrix, we extracted all the values representing nucleotide pairs with a distance of exactly four. We then log2 transformed the pairing probabilities (after adding a pseudo count of 0.0001) and grouped the values into seven bins. We then combined the structure preferences with the sequence preferences by enumerating all possible binding patterns based on the two sequence features I,II,II (64 combination) and – 1,1 (6 combinations: AU,UA,GC,GC,GU,UG), and the single structure feature (7 combinations). This resulted in 2688 (64 × 6 x 7) binding patterns. We inferred the contribution of each binding pattern to LIN41 binding by calculating the mean LIN41 binding enrichment of all the oligonucleotides on the array that contained the respective binding pattern. Due to the large number of RNAs with low enrichment values, any systematic sequence bias can potentially dominate the model output. Indeed, when inspecting the binding enrichment heatmap (Supplementary Fig. 7b) we noticed that almost one half of the features showed slightly negative contributions to binding while the other half showed slightly positive values. Most prominently, this was the case for the loop sequences AAA and CCC where we could detect a pervasive positive or negative signal, respectively. To gain more insight into this issue we visualized the RNAcomplete enrichments for all RNAs containing an AAA or a CCC and compared this with the enrichments from all the RNAs (Supplementary Fig. 7c). RNAs containing an AAA showed a slight shift in the distribution present at even low enrichment values ( < 1). CCC showed the opposite trend. Such an effect could be caused by a sequence bias in the pulldown experiment that is not perfectly controlled for by the input (obtained from a large compendium of other RNAcompete experiments). To avoid a dominating impact of the large number of RNAs with low enrichment, we simply set all RNAcompete enrichment values between – 2 and 2 to zero and recalculated the contributions for each binding pattern (Fig. 6b). This effectively reduced the aforementioned artifact (Supplementary Fig. 7b). As an alternative approach, we could have divided the oligonucleotides into a bound and unbound fraction using a cutoff of two, but by doing so we would have lost information about the extent of binding. We thus chose the former solution by setting a threshold to exclude the small enrichment values (between – 2 and 2). Based on the corrected contributions for the 2688 binding patterns (Fig. 6b), we then selected the ones with the most convincing binding signals. For U–A at position – 1/ + 1, we selected all the entries that showed pairing probabilities of at least level 4–7 and a binding score of at least 0.1. For C–G at position – 1/ + 1, we observed substantially lower contribution to the binding, therefore we only selected the entries that showed pairing probabilities of at least level 7 and a score of at least 0.1 (Fig. 6b). For downstream analysis of the predicted sites, we grouped them into four bins (minimal, weak, medium, strong) according the score from the *C. elegans* LRE model using twofold threshold steps (0.225, 0.45, 0.9, 1.8). For downstream analysis, we considered the group “minimal” as a control, for which we would expect close to no binding.

### Scanning the ***C. elegans*** transcriptome for predicted LREs

We used the same scanning procedure as for the RNAs in the RNAcompete assay but replaced the RNA folding program RNAfold (2.1.5) by RNAplfold (2.1.5), using the parameter -c 0 to report also very low pairing probabilities. The change to RNAplfold allowed us to efficiently fold not only short RNAs but also the full-length transcripts. For *C. elegans*, we used transcript annotations from WormBase (WS259) and extracted 5′-UTR, CDS, and 3′-UTR information from the file c_elegans.PRJNA13758.WS259.annotations.gff2.gz.

### RNA co-immunoprecipitation

RNA co-immunoprecipitation (RIP) was performed on total lysates from *lin-41(rrr3)* animals expressing a single copy rescuing FLAG-GFP-LIN-41 transgene. Worm pellets were prepared by harvesting worms (grown in two different weeks on enriched peptone plates and fed *E. coli* OP50 bacteria) and freezing in liquid nitrogen. Lysates from two biological replicates were prepared by grinding the frozen worm pellets using mortar and pestle, and dissolving in lysis buffer (50 mM Hepes pH 7.5, 150 mM KCl, 5 mM MgCl_2_, 0.1 % Triton X-100, and 10% glycerol w/vol), supplemented with Complete EDTA-free protease inhibitors (Roche, 11836153001) and 200 U/ml RNase inhibitor (RNaseIN, Promega N2111). Lysates were cleared by centrifugation at 20,000 × *g* for 30 min at 4 °C. IPs were performed by incubating lysates equivalent to 9 mg total protein with 20 μl of anti-FLAG M2 magnetic beads (Sigma–Aldrich; M8823) for 3 h at 4 °C while rotating. Beads were washed with lysis buffer supplemented with 300 mM NaCl and then bound RNP complexes were extracted by adding 100 µl of freshly prepared elution buffer (50 mM Tris-Cl pH 7, 5 mM EDTA, 10 mM dithiothreitol (DTT), 1% SDS). RNA was extracted from the eluate using picopure RNA isolation kit (Thermofischer Scientific KIT0204) according to the manufacturer’s instructions. Library preparation and sequencing was performed as described^44^.

### Processing the RIP-seq data

Gene intensities were quantified as described previously^45^ using the *C. elegans* genome assembly ce10 and gene annotation from WormBase (WS220). After normalization for library size, log2 expression levels were calculated after adding a pseudocount of 8 (*y* = log2[*x* + 8]). To account for nonlinear scaling between the samples, we quantile-normalized the data using the function normalize.quantiles from the R package preprocessCore. IP enrichments were then calculated by subtracting the input samples from the respective IP samples. For the comparison with the predicted CeLIN41 sites, non-expressed genes (expression < 5 in the input) were removed.

### RNA secondary structure predictions for UTRs

The RNA secondary structures were predicted using RNA Structure software version 5.8.1^46^. All of the secondary structures that were predicted within 10% of free energy of the most stable variant were considered.

### Electrophoretic mobility or gel-shift assays

Radioactively labeled probes for electrophoretic mobility shift assays (EMSAs) were transcribed from PCR products with T3 RNA polymerase. Templates for probe synthesis were generated by PCR with an extended phage T3 RNA polymerase promoter (5′-AATTAACCCTCACTAAAGGGAGAA-2′), appended to the 5′-end of the forward primer, and gel-purified. EMSA assay was performed as described earlier^10^. Each binding experiment was performed two to three times with freshly trasncribed RNA. Briefly, 1 µl of 5 µM protein was pre-incubated with 4 µl of 2 × gel-shift buffer (20 mM Hepes pH 8, 100 mM KCl, 200 mM NaCl, 0.2 mM EDTA, 20 mM DTT, 2 mM MgCl_2_, 2 mM CaCl_2_, 0.2 mM ZnSO_4_, 60 % glycerol, 125 µg/ml heparin, 50 µg/ml *E. coli* transfer RNA). The reaction was made up to 7 µl with sterile water, incubated for 10 min at room temperature, following which 1 µl of RNA probe (~ 2 nM, ~ 10^5^ cpm) was added. The reaction was incubated for 30 min and loaded onto the gel, electrophoresed at 25 mA, dried, and auto-radiographed.

### Fluorescence polarization

To measure the binding affinity of the LIN41–LRE complex, a series of binding reactions were set up with varying concentrations of protein and a fixed amount of 5′-Cy5 conjugated RNA (100 nM) in the assay buffer (20 mM HEPES, 200 mM NaCl, 0.05% Tween20) (Supplementary Table 5). After equilibration for 30 min at room temperature, polarization was determined using a fluorescence plate reader equipped with polarizers. Three technical replicates were measured for each binding reaction. Polarization is expressed in the units of millipolarization (mP) and the baseline was set to 50 mP for unbound RNA. Binding to protein did not change the total intensity of RNA probe and the ratio of total intensity emitted by the RNA in bound state to free state was close to one. The raw polarization values were plotted and fitted to Bdg Mueller equation [*Y* = *B* + *A* × (*K* + *L* + *X* – ((*K* + *L* + *X*)^2^ – 4 × *L* × *X*)^0.5^)/(2 × *L*)] to determine the equilibrium dissociation constant (K) using GraphPad Prism7.

### Construction of GFP reporters

The GFP reporter plasmids were cloned with the MultiSite Gateway Technology (Thermo Fisher Scientific) and the destination vector pCFJ150^47^. Modified 5′-UTRs and 3′-UTR fragments were ordered as gBlocks® Gene Fragments (Integrated DNA Technologies) and cloned into Entry clones using Gibson assembly^48^. Supplementary Data 1 lists gBlock sequences and resulting entry plasmids. For every final reporter plasmid, three entry plasmids and the pCFJ150 vector backbone were recombined (Gateway LR Clonase II Enzyme mix, Thermo Fisher Scientific; 11791020) resulting in a plasmid containing a promoter, 5′-UTR, GFP(PEST)-H2B coding sequence and a 3′-UTR. Transgenic animals were obtained by single-copy integration into the *ttTi5605* locus on chromosome II, using the protocol for injection with low DNA concentration^49^.

### Confocal imaging

Synchronized L1 larvae were grown at 25 °C on RNAi-inducing plates with HT115 bacteria for 20 h and then subjected to confocal imaging. The HT115 bacteria either contained the insert-less L4440 parental RNAi vector (denoted “mock RNAi”) or an RNAi vector with an insert targeting *lin-41*^50^. Worms were mounted on a 2% (w/v) agarose pad with a drop of 10 mM levamisole solution and imaged on a Zeiss LSM 700 confocal microscope driven by Zen 2012 Software. Before acquiring images of representative worms, the GFP signals for at least ten worms were observed to verify that they were comparable among different worms in each worm line and for each condition. Fluorescent and differential interference contrast images were acquired with a × 40/1.3 oil-immersion objective (1024 × 1024 pixels, pixel size 156 nm). Selections of representative regions and processing of images was performed with Fiji^51^. Identical worm lines grown on mock or *lin-41* RNAi bacteria were imaged and processed with identical settings.

### Luciferase assay

pCl-neo-TRIM71 (HsLIN41) plasmid is a kind gift from Loedige et al.^11^. The 3′-UTRs were cloned into pRLTK (RL) plasmid using the XbaI and NotI sites. Primers are listed in Supplementary Data 1. HEK-293 (gift from Yoshikuni Nagamine's laboratory) cells were grown in Dulbecco’s modified Eagle’s medium supplemented with 10% heat-inactivated fetal calf serum and were cultured as described^52^. Five hundred nanograms of pCl-neo-TRIM71 and 100 ng each of pRLTK and pFLTK (FL) plasmids were transfected using Effectene transfection reagent (Qiagen, 301425), following the manufacturer’s instructions. Transfection was done in three independent biological replicates of cells. Luciferase assay was done using dual-luciferase reporter assay system (Promega, E1910), following the manufacturer’s instructions. For each measurement, average of three technical replicates was used for further analysis.

### Western blotting

HEK293 cell lysates previously used for the dual luciferase assay were thawed on ice and cleared by centrifugation at 4 °C for 10 min at 16,000 × *g*. The supernatants were transferred to fresh Eppendorf tubes and protein concentrations determined by nanodrop. Two hundred micrograms of protein was mixed with SDS lysis buffer (63 mM Tris-HCl pH 6.8, 5 mM DTT, 2% SDS, 5% sucrose) and boiled for 5 min at 95 °C. Proteins were separated by SDS-PAGE (loading: 50 µg protein extract per well) and transferred to polyvinylidene difluoride membranes by semi-dry blotting. The following antibodies were used: monoclonal rat anti-HA clone 3F10 (Sigma-Aldrich; catalog number 11867423001, dilution: 1:2,000) and monoclonal rat anti-Tubulin [YL1/2] (abcam; catalog number ab6160, dilution 1:10,000). Detection was performed with a horseradish peroxidase-conjugated secondary antibody (Jackson ImmunoResearch; catalog number 712-035-153, dilution 1:15,000), ECL Western Blotting Detection Reagents (GE Healthcare), and an Amersham 600 chemiluminescence imager (GE Healthcare). Western blotting was performed for all the three biological replicates. One representative blot is shown in the results.

### Expression and purification of the LIN41 proteins

The DNA sequence encoding *D.* *rerio* LIN41 filamin and NHL domains was taken from the NCBI reference sequence NM_001301331 (nucleotides 2040–3209) and synthesized (GeneArt) without any codon optimization. This DNA template was cloned into pOPINF using In-Fusion cloning^53^ to yield pOPINF_DrLIN41 (435–824). The translated sequence corresponds to Uniprot E7FAM5 (amino acids 435–824) with an N-terminal 3C cleavable histidine tag. Protein expression was carried out in Sf9 insect cells using pOPINF_DrLIN41 (435–824) in the FlashBAC baculovirus system. His-tagged DrLIN41 filamin-NHL protein was extracted from a baculovirus-infected Sf9 cell pellet by thoroughly re-suspending the cells in ice-cold nickel lysis buffer (50 mM Tris pH 8.0, 150 mM NaCl, 10 mM imidazole, 1 mM TCEP, 2.5 mM MgCl_2_, 0.2% Tween-20), freshly supplemented with Complete EDTA-free protease inhibitors (Roche, 11836153001) and Benzonase (Sigma, E1014). After 30 min on ice, the lysate was centrifuged at 30,000 × *g* for 30 min at 4 °C. The clarified soluble lysate was incubated in batch mode with Ni-NTA Superflow (Qiagen, 30430) and then transferred into a 10 ml Econo-Pac column (Bio-Rad, 7321010) for washing with nickel wash buffer (50 mM Tris pH 8.0, 500 mM NaCl, 20 mM imidazole, 1 mM TCEP). Elution was performed with nickel wash buffer containing 125 mM imidazole. The eluate was concentrated and separated on a HiLoad 16/600 Superdex 200 (GE Healthcare) gel filtration column equilibrated in 20 mM Tris pH 8.0, 200 mM NaCl, 2 mM TCEP, and 0.02 % NaN_3_. His-tagged DrLIN41 protein fractions were pooled, concentrated to 4.6 mg/ml, frozen in liquid nitrogen, and stored at – 80 °C.

For the CeLIN41 filamin and NHL domains (isoform B of Q9U489), a DNA insert encoding amino acids 691–1147 was cloned into pAc8^54^ to incorporate an N-terminal Strep tag followed by a TEV cleavage site. Protein expression and lysis was done as described above except the lysis buffer used was 100 mM Tris pH 8.0, 150 mM NaCl, 2 mM TCEP, 2 mM MgCl_2_, 0.2% Tween-20, 0.02% NaN_3_, freshly supplemented with Complete EDTA-free protease inhibitors (Roche, 11836153001) and Benzonase (Sigma, E1014). Clarified lysate was loaded onto a 5 ml StrepTrap HP column (GE Healthcare). The column was washed in three 10 ml steps using binding buffer (100 mM Tris, pH 8.0, 150 mM NaCl, 2 mM TCEP, 0.02% NaN_3_), high-salt wash buffer (binding buffer containing 1 M NaCl) followed by binding buffer. Elution was done using 10 ml binding buffer containing 2.5 mM desthiobiotin. The subsequent gel filtration, concentration, and storage steps were as described above.

### Crystallization and structure solution

His-tagged *D. rerio* LIN41 protein consisting of the filamin and NHL domains (residues 435–824) was crystallized using the sitting-drop vapor diffusion method at 20 °C with a Phoenix nano-liter dispensing robot (Art Robbins). One hundred nanoliters of DrLIN41 protein at 4.6 mg/ml in protein buffer (0.02 M Tris pH 8.0, 0.2 M NaCl, 0.002 M TCEP, 0.02% NaN_3_) was mixed with 100 nl of crystallization buffer (0.2 M MgCl_2_, 0.1 M Tris pH 7.0, 10% (w/v) PEG 8000, 0.1 M Na_3_ Citrate). Long rectangular plate-type crystals measuring ca. 300 × 35 μm were obtained after 10 days. Crystals were cryo-protected in crystallization buffer containing 30% ethylene glycol followed by cryo-cooling in liquid nitrogen. For the DrLIN41–RNA complexes, it was necessary to screen different RNA targets and sequence lengths to obtain good diffracting crystals. RNA was chemically synthesized, purified by PAGE, deprotected to the 2′-hydroxyl form, desalted, and lyophilized (Dharmacon). Stock concentrations of RNA (30 mM) were made in 0.1 M Tris pH 8.0. To promote the formation of SLs the RNA was heated to 90 °C for 5 min and then rapidly cooled on ice for 30 min. The RNA was then added in a tenfold molar excess to the freshly purified His-tagged *D. rerio* LIN41 filamin-NHL protein (107 µM) and incubated for 1 h on ice. The RNA–protein complex was passed through a 0.1 µm Ultrafree-MC filter (Millipore) before setting up crystallization experiments as described above. *D. rerio* LIN41 filamin-NHL with *lin-29A* RNA (5′-GGAGUCCAACUCC-3′) crystallized overnight in Morpheus HT-96 (Molecular Dimensions Ltd, UK) condition G10 (10% w/v PEG 8000, 20% v/v ethylene glycol, 0.1 M carboxylic acids, 0.1 M bicine/Trizma base pH 8.5). *D. rerio* LIN41 filamin-NHL with *mab-10* RNA (5′-UGCAUUUAAUGCA-3′) crystallized after 13 days in similar conditions from the same crystallization screen, condition G2 (10% w/v PEG 8000, 20 % v/v ethylene glycol, 0.1 M carboxylic acids, 0.1 M MES/imidazole pH 6.5). Crystals were harvested after 27 days growth at 20 °C and cryo-cooled in liquid nitrogen.

X-ray data collection was carried out at SLS PX-II/III beamlines in Villigen, Switzerland, at 100 K. DrLIN41 filamin-NHL protein crystals diffracted to 2.6 Å and belonged to space group P2_1_2_1_2_1_ with two molecules per a.u. Crystals of DrLIN41 (filamin-NHL) in complex with *lin-29A*/*mab-10* RNAs belonged to space group P3_1_21 with one complex per a.u. and diffracted to 1.9 Å and 2.35 Å, respectively. Diffraction data were integrated and scaled using the XDS program package^55^ and the DrLIN41 filamin-NHL unbound structure was solved by the molecular replacement method with PHASER^56^ using truncated homology models of the filamin and NHL domain as search models. DrLIN41 (filamin-NHL) RNA complex structures were solved by molecular replacement using filamin and NHL domains of the unbound structure as search models. Clear mFo-DFc electron density for the missing RNA part allowed unambiguous building of the hairpin. This was further supported by a phased anomalous difference Fourier map calculated from a separately collected highly redundant data set (overall redundancy = 27, overall *I*/*σ*(*I*) = 23.9) at longer wavelength (1.5 Å, *f*” = 0.414 for phosphorus) for the lin-29A complex which featured clear peaks for all phosphorus atoms of the RNA sugar phosphate backbone. All structures were then manually completed and further improved by the crystallographic simulated annealing routine followed by individual B-factor refinement in PHENIX^57^. Structures were then finalized by several rounds of manual rebuilding in COOT^58^ and refinement in BUSTER^59^ (unbound) and PHENIX (RNA complexes). Final structures were validated using tools implemented in COOT. Structural images for figures were prepared with PyMOL (http://pymol.sourceforge.net/).

### Bioinformatics analysis of NHL domains

To address the question of differential RNA-binding properties of the NHL domain-containing proteins, a comprehensive bioinformatics analysis of Brat, LIN41, and other known NHL domain containing RBPs was carried out. Sequence database searches were carried out with the DmBrat and the LIN41 protein from *D. rerio* (DrLIN41) sequences as queries, against the non-redundant protein sequence database. This led to the identification of a large group of 3762 closely related sequences. Partial sequences, highly identical sequences from the same organism and smaller isoforms were removed. Based on a preliminary multiple sequence alignment, the NHL domain was extracted. Eventually, 71 representative sequences of NHL domains were selected from NHL-domain-containing protein in higher eukaryotes (mostly model organisms with fully sequenced genomes) and a few prokaryotes for further analyses.

A multiple sequence alignment of selected NHL domains was calculated using PROMALS3D^60^, which takes into account both sequence and structural information. We performed the analysis iteratively, first by calculating the alignment of NHL domain sequences with a support of experimentally determined structures of DmBrat and DrLIN41, and later refined it using also predicted 3D structures of other subfamily members identified by phylogenetic analysis. To elucidate the phylogenetic relationships of NHL domains, the Minimum Evolution phylogenetic tree was calculated on the basis of alignment of the NHL domain sequences of the 71 selected proteins, including DmBrat and DrLIN41. The robustness of branches was assessed by calculating 1000 bootstrap replicates.

To provide insight into functional properties of NHL domains and to aid in the determination of their evolutionary relationships, we generated 3D structural models of representatives from five so far structurally uncharacterized subfamilies: LIN-41 from *C. elegans* (CeLIN41), DmWech, CeNHL1, and TRIM3 from *Homo sapiens* (HsTRIM3) and DmMei-P26 proteins. To this end, a series of alignments to proteins of known structure were obtained through the GeneSilico meta-server^61^ using various protein fold recognition methods. The meta-server consistently reported the structure of DmBrat as the potentially best template for modeling of the NHL domains, and we used the variant in complex with RNA (PDB code: 5EX7) for the modeling of the CeNHL1, HsTRIM3, and DmMei-P26 proteins. The DrLIN41 structure is obtained during this study and was not available in the database for the Fold Recognition analysis. However, it was used as the template for the modeling of CeLIN41 and the DmWech proteins, due to their closer phylogenetic relationship. Comparative modeling as well as structure analysis was performed using SwissPDBViewer and SwissModel^62^. Evolutionary sequence conservation among the various subgroups of the NHL domain-containing proteins were analyzed using ConSurf^63^ and were mapped on the NHL domain structure of DrLIN41 (Fig. 7, right panel, second column) and DmBrat (Fig. 7, right panel, third column).

### Data availability

Atomic coordinates and structure factors have been deposited in the Protein Data Bank under accession codes 6FPT (DrLin41 unbound), 6FQ3 (DrLin41-lin-29A complex), and 6FQL (DrLin41-mab-10 complex). All RNA-sequencing data generated in this study have been deposited in the NCBI Gene Expression Omnibus^64^ under GEO Series accession number GSE106814. Other datasets generated during and/or analyzed during the current study and computer codes are available from the corresponding authors on reasonable request.

## Electronic supplementary material

Supplementary Information

Peer Review Report

Description of Additional Supplementary Info

Supplementary Data 1

## References

[1] Insco ML (2012). A self-limiting switch based on translational control regulates the transition from proliferation to differentiation in an adult stem cell lineage. Cell Stem Cell.

[2] Bowman SK (2008). The tumor suppressors Brat and Numb regulate transit-amplifying neuroblast lineages in *Drosophila*. Dev. Cell.

[3] Schwamborn JC, Berezikov E, Knoblich JA (2009). The TRIM-NHL protein TRIM32 activates microRNAs and prevents self-renewal in mouse neural progenitors. Cell.

[4] Tocchini C, Ciosk R (2015). TRIM-NHL proteins in development and disease. Semin. Cell. Dev. Biol..

[5] Wulczyn FG, Cuevas E, Franzoni E, Rybak A (2010). MiRNA need a TRIM regulation of miRNA activity by Trim-NHL proteins. Adv. Exp. Med. Biol..

[6] Slack FJ (2000). The lin-41 RBCC gene acts in the C. elegans heterochronic pathway between the let-7 regulatory RNA and the LIN-29 transcription factor. Mol. Cell.

[7] Ecsedi M, Rausch M, Grosshans H (2015). The let-7 microRNA directs vulval development through a single target. Dev. Cell.

[8] Tocchini C (2014). The TRIM-NHL protein LIN-41 controls the onset of developmental plasticity in *Caenorhabditis elegans*. PLoS Genet..

[9] Worringer KA (2014). The let-7/LIN-41 pathway regulates reprogramming to human induced pluripotent stem cells by controlling expression of prodifferentiation genes. Cell Stem Cell.

[10] Aeschimann F (2017). LIN41 Post-transcriptionally Silences mRNAs by Two Distinct and Position-Dependent Mechanisms. Mol. Cell..

[11] Loedige I, Gaidatzis D, Sack R, Meister G, Filipowicz W (2013). The mammalian TRIM-NHL protein TRIM71/LIN-41 is a repressor of mRNA function. Nucleic Acids Res..

[12] Castello A (2012). Insights into RNA biology from an atlas of mammalian mRNA-binding proteins. Cell.

[13] Ghosh P, Sowdhamini R (2016). Genome-wide survey of putative RNA-binding proteins encoded in the human proteome. Mol. Biosyst..

[14] Baltz AG (2012). The mRNA-bound proteome and its global occupancy profile on protein-coding transcripts. Mol. Cell.

[15] Loedige I (2014). The NHL domain of BRAT is an RNA-binding domain that directly contacts the hunchback mRNA for regulation. Genes Dev..

[16] Kwon SC (2013). The RNA-binding protein repertoire of embryonic stem cells. Nat. Struct. Mol. Biol..

[17] Loedige I (2015). The crystal structure of the NHL domain in complex with RNA reveals the molecular basis of *Drosophila* brain-tumor-mediated gene regulation. Cell Rep..

[18] Laver JD (2015). Brain tumor is a sequence-specific RNA-binding protein that directs maternal mRNA clearance during the Drosophila maternal-to-zygotic transition. Genome Biol..

[19] Ray D (2009). Rapid and systematic analysis of the RNA recognition specificities of RNA-binding proteins. Nat. Biotechnol..

[20] Ray D (2013). A compendium of RNA-binding motifs for decoding gene regulation. Nature.

[21] Solana, J. et al. Conserved functional antagonism of CELF and MBNL proteins controls stem cell-specific alternative splicing in planarians. *eLife* **5**, pii: e16797 (2016). 10.7554/eLife.16797 PMC4978528 27502555

[22] Dang H (2017). Oncogenic activation of the RNA binding protein NELFE and MYC signaling in hepatocellular carcinoma. Cancer Cell.

[23] Collins KM (2017). An RRM-ZnF RNA recognition module targets RBM10 to exonic sequences to promote exon exclusion. Nucleic Acids Res..

[24] Lorenz R (2011). ViennaRNA Package 2.0. Algorithms Mol. Biol.: AMB.

[25] Krissinel E, Henrick K (2007). Inference of macromolecular assemblies from crystalline state. J. Mol. Biol..

[26] Duarte JM, Srebniak A, Scharer MA, Capitani G (2012). Protein interface classification by evolutionary analysis. BMC Bioinformatics.

[27] Holm L, Rosenstrom P (2010). Dali server: conservation mapping in 3D. Nucleic Acids Res..

[28] Edwards TA, Wilkinson BD, Wharton RP, Aggarwal AK (2003). Model of the brain tumor-Pumilio translation repressor complex. Genes Dev..

[29] Koliopoulos MG, Esposito D, Christodoulou E, Taylor IA, Rittinger K (2016). Functional role of TRIM E3 ligase oligomerization and regulation of catalytic activity. EMBO J..

[30] Spike CA (2014). The TRIM-NHL protein LIN-41 and the OMA RNA-binding proteins antagonistically control the prophase-to-metaphase transition and growth of *Caenorhabditis elegans* oocytes. Genetics.

[31] Tsukamoto T (2017). LIN-41 and OMA ribonucleoprotein complexes mediate a translational repression-to-activation switch controlling oocyte meiotic maturation and the oocyte-to-embryo transition in *Caenorhabditis elegans*. Genetics.

[32] Matsuura R, Ashikawa T, Nozaki Y, Kitagawa D (2016). LIN-41 inactivation leads to delayed centrosome elimination and abnormal chromosome behavior during female meiosis in *Caenorhabditis elegans*. Mol. Biol. Cell.

[33] Lancman JJ (2005). Analysis of the regulation of lin-41 during chick and mouse limb development. Dev. Dyn..

[34] Maller Schulman BR (2008). The let-7 microRNA target gene, Mlin41/Trim71 is required for mouse embryonic survival and neural tube closure. Cell Cycle.

[35] Lee SH (2014). The ubiquitin ligase human TRIM71 regulates let-7 microRNA biogenesis via modulation of Lin28B protein. Biochim. Biophys. Acta.

[36] Nguyen DTT (2017). The ubiquitin ligase LIN41/TRIM71 targets p53 to antagonize cell death and differentiation pathways during stem cell differentiation. Cell Death Differ..

[37] Chen J, Lai F, Niswander L (2012). The ubiquitin ligase mLin41 temporally promotes neural progenitor cell maintenance through FGF signaling. Genes Dev..

[38] Loer B (2008). The NHL-domain protein Wech is crucial for the integrin-cytoskeleton link. Nat. Cell. Biol..

[39] Treiber T (2017). A compendium of RNA-binding proteins that regulate microRNA biogenesis. Mol. Cell.

[40] Kashyap M, Ganguly AK, Bhavesh NS (2015). Structural delineation of stem-loop RNA binding by human TAF15 protein. Sci. Rep..

[41] Skrisovska L (2007). The testis-specific human protein RBMY recognizes RNA through a novel mode of interaction. EMBO Rep..

[42] Law MJ, Rice AJ, Lin P, Laird-Offringa IA (2006). The role of RNA structure in the interaction of U1A protein with U1 hairpin II RNA. RNA.

[43] Ahringer, J. (ed.) *WormBook* 1–43 (The Gurdon Institute, Univ. Cambridge: Cambridge, UK, 2006).

[44] Arnold A (2014). Functional characterization of *C. elegans* Y-box-binding proteins reveals tissue-specific functions and a critical role in the formation of polysomes. Nucleic Acids Res..

[45] Hendriks GJ, Gaidatzis D, Aeschimann F, Grosshans H (2014). Extensive oscillatory gene expression during *C. elegans* larval development. Mol. Cell.

[46] Deigan KE, Li TW, Mathews DH, Weeks KM (2009). Accurate SHAPE-directed RNA structure determination. Proc. Natl Acad. Sci. USA.

[47] Frokjaer-Jensen C (2008). Single-copy insertion of transgenes in *Caenorhabditis elegans*. Nat. Genet..

[48] Gibson DG (2009). Enzymatic assembly of DNA molecules up to several hundred kilobases. Nat. Methods.

[49] Frokjaer-Jensen C, Davis MW, Ailion M, Jorgensen EM (2012). Improved Mos1-mediated transgenesis in *C. elegans*. Nat. Methods.

[50] Fraser AG (2000). Functional genomic analysis of *C. elegans* chromosome I by systematic RNA interference. Nature.

[51] Schindelin J (2012). Fiji: an open-source platform for biological-image analysis. Nat. Methods.

[52] Sinkkonen L (2008). MicroRNAs control de novo DNA methylation through regulation of transcriptional repressors in mouse embryonic stem cells. Nat. Struct. Mol. Biol..

[53] Berrow NS, Alderton D, Owens RJ (2009). The precise engineering of expression vectors using high-throughput In-Fusion PCR cloning. Methods Mol. Biol..

[54] Abdulrahman W (2009). A set of baculovirus transfer vectors for screening of affinity tags and parallel expression strategies. Anal. Biochem..

[55] Kabsch W (2010). XDS. Acta Crystallogr. D Biol. Crystallogr..

[56] McCoy AJ (2007). Phaser crystallographic software. J. Appl. Crystallogr..

[57] Afonine PV (2010). Joint X-ray and neutron refinement with phenix.refine. Acta Crystallogr. D Biol. Crystallogr..

[58] Emsley P, Lohkamp B, Scott WG, Cowtan K (2010). Features and development of Coot. Acta Crystallogr. D Biol. Crystallogr..

[59] Bricogne G (2017). T.O..

[60] Pei J, Tang M, Grishin NV (2008). PROMALS3D web server for accurate multiple protein sequence and structure alignments. Nucleic Acids Res..

[61] Kurowski MA, Bujnicki JM (2003). GeneSilico protein structure prediction meta-server. Nucleic Acids Res..

[62] Guex N, Peitsch MC (1997). SWISS-MODEL and the Swiss-PdbViewer: an environment for comparative protein modeling. Electrophoresis.

[63] Ashkenazy H (2016). ConSurf 2016: an improved methodology to estimate and visualize evolutionary conservation in macromolecules. Nucleic Acids Res..

[64] Edgar R, Domrachev M, Lash AE (2002). Gene Expression Omnibus: NCBI gene expression and hybridization array data repository. Nucleic Acids Res..

