# Supplementary material for: Evolutionary plasticity of the NHL domain underlies distinct solutions to RNA recognition
Source: Nat Commun. 2018 Apr 19;9:1549. doi: 10.1038/s41467-018-03920-7 (PMC5908797; doi:10.1038/s41467-018-03920-7)
Supplement: Supplementary file 1 — Supplementary Information [file 41467_2018_3920_MOESM1_ESM.pdf]

Supplementary Information

Evolutionary plasticity of the NHL domain underlies distinct solutions to RNA recognition

Kumari et al.

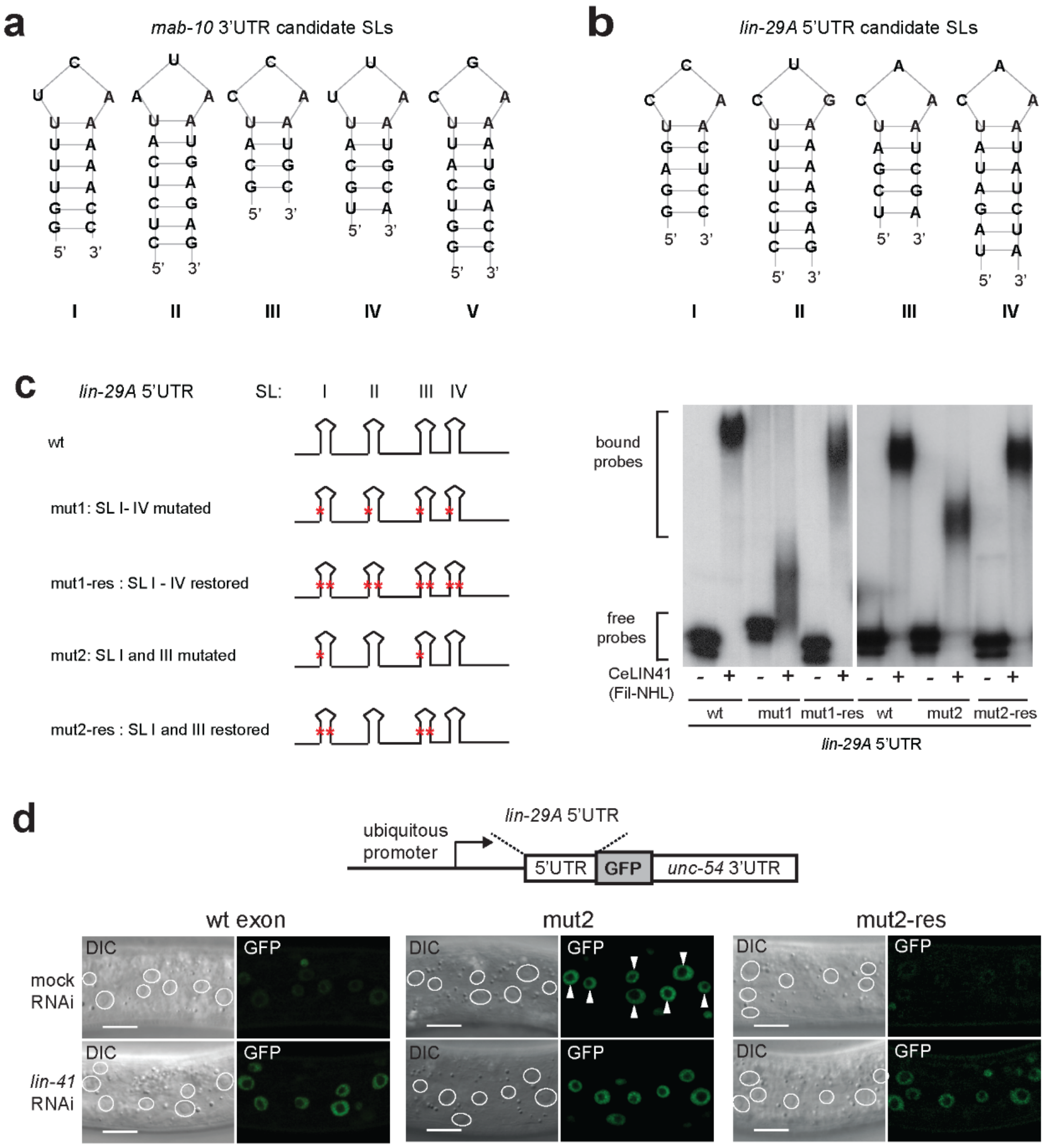
a
mab-10 3'UTR candidate SLs
I
II
III
IV
V
b
lin-29A 5'UTR candidate SLs
I
II
III
IV
c
lin-29A 5'UTR
SL: I II III IV
wt
mut1: SL I- IV mutated
mut1-res : SL I - IV restored
mut2: SL I and III mutated
mut2-res : SL I and III restored
bound probes
free probes
CeLIN41 (Fil-NHL)
wt
mut1
mut1-res
wt
mut2
mut2-res
lin-29A 5'UTR
d
ubiquitous promoter
lin-29A 5'UTR
5'UTR
GFP
unc-54 3'UTR
wt exon
mut2
mut2-res
mock RNAi
lin-41 RNAi
DIC
GFP

Supplementary Figure 1: CeLIN41 binds to SL motifs of *mab-10* 3'UTR and *lin-29A* 5'UTR. Related to Fig. 2

(**a**) Sequences of candidate CeLIN41 binding SLs in the *mab-10* 3'UTR.

(**b**) Sequences of candidate CeLIN41 binding SLs in the *lin-29A* 5'UTR exon-1.

(**c**) CeLIN41 binding to *lin-29A* 5'UTR. Left: Schematics representing wild-type and modified *lin-29A* RNA probes. Respective sequences are listed in Supplementary Table 1. Right: Gel-shift experiments showing binding of CeLIN41 to the *lin-29A* RNA probes described in the schematics on the left. Whereas CeLIN41 bound to the *lin-29A* 5'UTR, mutations disrupting all the four SLs (mut1) significantly reduced the binding to CeLIN41. Mutations disrupting SL I and III (mut2) led to the formation of a smaller protein-RNA complex as compared to the wild-type. Compensatory mutations in the stem (mut1-res and mut2-res) restored the protein-RNA complex to wild-type levels.

(**d**) Micrographs of early L3-stage *C. elegans* larvae, treated with either *lin-41* or mock RNAi expressing nuclear-localized GFP (to concentrate the signal; white circles demarcate nuclei) from the *dpy-30* promoter and under an unregulated 3'UTR, *unc-54*. The 5'UTR corresponds to the constructs in **c**. GFP was imaged under identical microscope settings for all animals. The wild-type but not the mutant (mut2) *lin-29A* 5'UTR imposed CeLIN41-mediated repression on the GFP (white arrowheads point to GFP-expressing nuclei). The restored 5'UTR (mut2-res) reinstated CeLIN41-mediated regulation comparable to the wild-type levels. Scale bars - 10 µm.

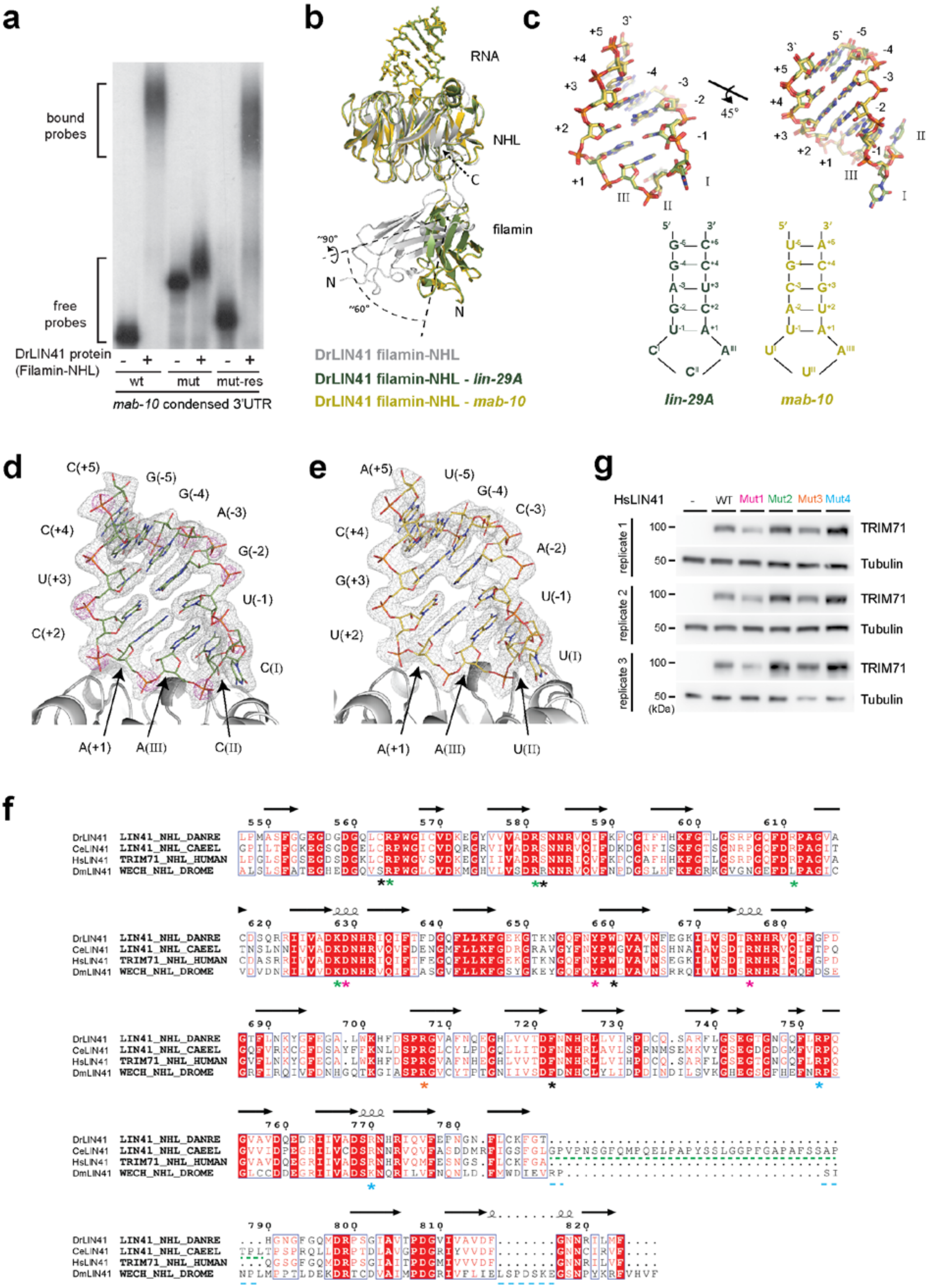
a
bound probes
free probes
DrLIN41 protein (Filamin-NHL)
- + - + - +
wt mut mut-res
mab-10 condensed 3'UTR
b
RNA
NHL
C
filamin
~90°
~60°
N
N
DrLIN41 filamin-NHL
DrLIN41 filamin-NHL - lin-29A
DrLIN41 filamin-NHL - mab-10
c
45°
lin-29A
mab-10
d
C(+5) G(-5) G(-4) A(-3) C(+4) G(-2) U(+3) U(-1) C(+2) C(I)
A(+1) A(III) C(II)
e
A(+5) U(-5) G(-4) C(-3) C(+4) A(-2) G(+3) U(-1) U(+2) U(I)
A(+1) A(III) U(II)
g
HsLIN41
- WT Mut1 Mut2 Mut3 Mut4
replicate 1
replicate 2
replicate 3
100
50
(kDa)
TRIM71
Tubulin
f
DrLIN41 LIN41_NHL_DANRE
CeLIN41 LIN41_NHL_CAEEL
HsLIN41 TRIM71_NHL_HUMAN
DmLIN41 WECH_NHL_DROME

Supplementary Figure 2: Crystal structures of DrLIN41 filamin-NHL domain and conformation of RNA stem loops. Related to Fig. 4 and 5

(**a**) Gel-shift experiment showing the binding of DrLIN41 to the *mab-10* condensed 3'UTR probes described in the schematics in Fig. 2c. Whereas DrLIN41 bound the *mab-10* condensed 3'UTR, mutations disrupting the stem (mut) abolished the binding, whereas compensatory mutations (mut-res) restored the binding.

(**b**) Superposition of DrLIN41 filamin-NHL crystal structures (unbound: grey, LIN41-*lin-29A* complex: olive, LIN41-*mab-10* complex: gold). The protein is displayed as cartoon model and RNA is shown as sticks. Differences in the position of the filamin domain between unbound and complex structures are highlighted; protein domains and RNA are labeled.

(**c**) Detailed presentation of superimposed *lin-29A* (olive, sticks in atom colors) and *mab-10* (gold, sticks in atom colors) stem loop RNAs as present in the LIN41 RNA complex structures. Two views are shown where the RNA is rotated by ~45 degrees along the indicated axis. RNA positions in the stem loop are labeled and schematic diagrams depicting RNA sequences are inserted below.

(**d, e**) Electron density maps for *lin-29A* and *mab-10* stem loop RNAs as present in the corresponding crystal structures of the DrLIN41 complexes. RNA molecules are displayed as in **b**, while the protein is shown as cartoon in grey. $2mF_o$-DF_c simulated annealing composite omit maps are shown in grey (1σ). An anomalous difference Fourier electron density map featuring peaks at phosphorus positions of the RNA backbone for *lin29-A* is displayed in magenta ($5\ \sigma$) in **d**. Nucleotide sequences are labeled.

(**f**) A ClustalO multiple sequence alignment between LIN41 NHL domains from *D. rerio* (LIN41_NHL_DANRE, Uniprot E7FAM5), *C. elegans* (LIN41_NHL_CAEEL, Uniprot Q9U489) *H. sapiens* (TRIM71_NHL_HUMAN, Uniprot Q2Q1W2), and *D. melanogaster* (WECH_NHL_DROME, Uniprot Q9V4M2), showing a high degree of sequence conservation. Secondary structure elements present in the DrLIN41 filamin-NHL crystal structure are displayed as arrows (β-strands) and spirals (3.10 helices) above the alignment. The *C. elegans* protein contains a 37 amino acid-long insert in the loop connecting propeller blades V and VI, which is highlighted with a dotted line in green. Shorter inserts are also found in the *Drosophila* protein and are highlighted by dotted lines in cyan. Residues involved in RNA SL binding in the DrLIN41-RNA complex crystal structures are marked with an asterisk under the alignment. Residues that were mutated to investigate their contribution to SL binding are colored as in Fig. 5c.

(**g**) Western blot showing expression levels of mutant HsLIN41 proteins used in Fig. 5d. Uncropped gels with markers are shown in Supplementary Fig. 8.

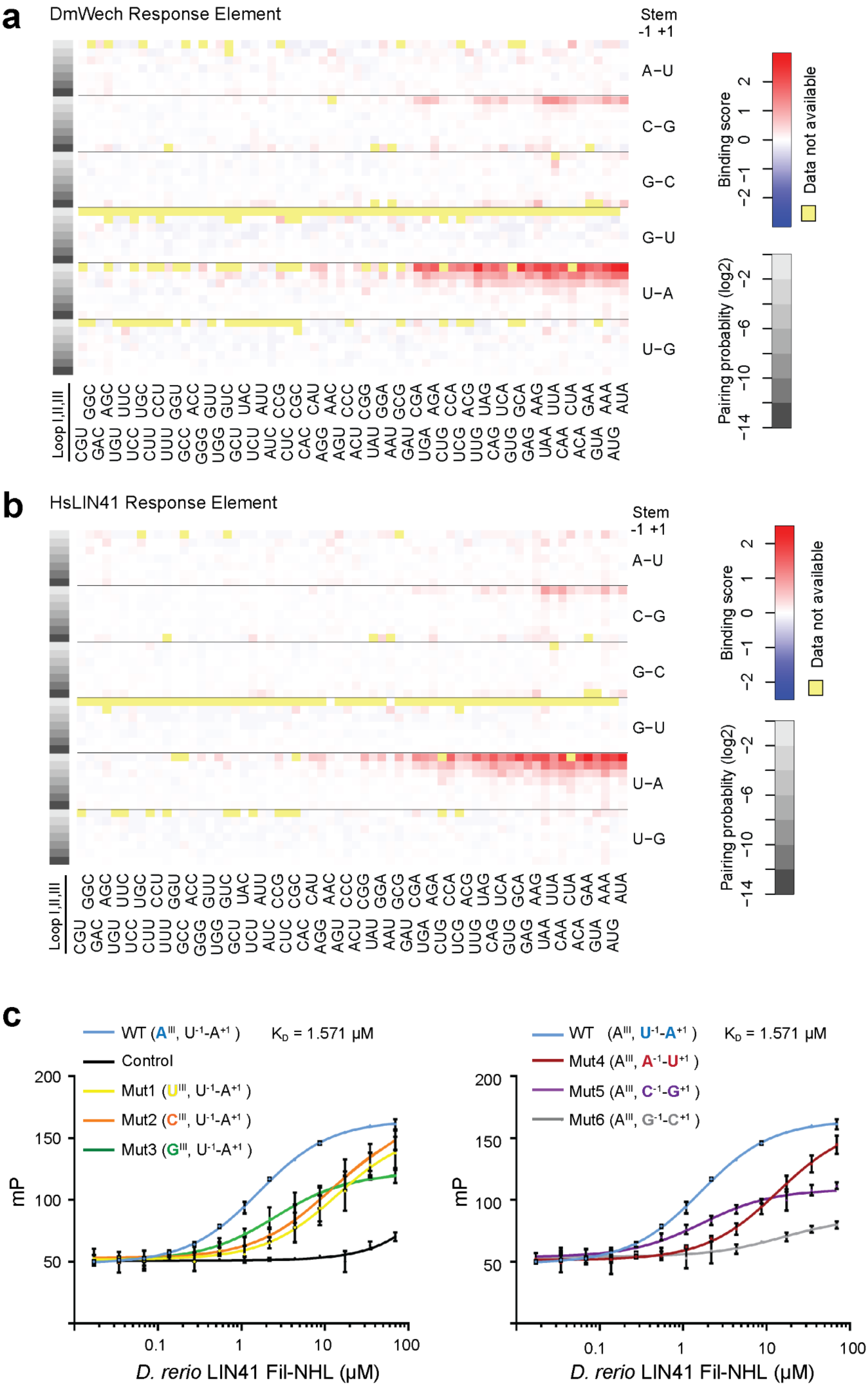
a
DmWech Response Element
Stem
-1 +1
A−U
C−G
G−C
G−U
U−A
U−G
Loop I,II,III
CGU GGC GAC AGC UGU UUC UCC UGC CUU CCU UUU GGU GCC ACC GGG GUU UGG GUC GCU UAC UCU AUU AUC CCG CUC CGC CAC CAU AGG AAC AGU CCC ACU CGG UAU GGA AAU GCG GAU CGA UGA AGA CUG CCA UCG ACG UUG UAG CAG UCA GUG GCA GAG AAG UAA UUA CAA CUA ACA GAA GUA AAA AUG AUA
Binding score
−2 −1 0 1 2
Data not available
Pairing probability (log2)
−14 −10 −6 −2
b
HsLIN41 Response Element
Stem
-1 +1
A−U
C−G
G−C
G−U
U−A
U−G
Loop I,II,III
CGU GGC GAC AGC UGU UUC UCC UGC CUU CCU UUU GGU GCC ACC GGG GUU UGG GUC GCU UAC UCU AUU AUC CCG CUC CGC CAC CAU AGG AAC AGU CCC ACU CGG UAU GGA AAU GCG GAU CGA UGA AGA CUG CCA UCG ACG UUG UAG CAG UCA GUG GCA GAG AAG UAA UUA CAA CUA ACA GAA GUA AAA AUG AUA
Binding score
−2 −1 0 1 2
Data not available
Pairing probability (log2)
−14 −10 −6 −2
c
WT (AIII, U-1-A+1)
KD = 1.571 μM
Control
Mut1 (UIII, U-1-A+1)
Mut2 (CIII, U-1-A+1)
Mut3 (GIII, U-1-A+1)
mP
200
150
100
50
0.1
1
10
100
D. rerio LIN41 Fil-NHL (μM)
WT (AIII, U-1-A+1)
KD = 1.571 μM
Mut4 (AIII, A-1-U+1)
Mut5 (AIII, C-1-G+1)
Mut6 (AIII, G-1-C+1)
mP
200
150
100
50
0.1
1
10
100
D. rerio LIN41 Fil-NHL (μM)

Supplementary Figure 3: LIN41 Response Element - LRE. Related to Fig. 6-7

(**a, b**) A heat map showing the average DmWech and HsLIN41 binding scores from the RNAcompete experiment for all RNAs containing any particular motif variant as described in Fig. 6a. The data was clustered based on the CeLIN41 binding score. Yellow: data not available (RNA motif variants supported by less than 20 oligonucleotide sequences).

(**c**) The FP assay to determine binding constants - Raw FP data of DrLIN41 interacting with a WT LRE (SL I in Supplementary Fig. 1b), a control stem-loop RNA with five nucleotides in the loop and mutant LREs are shown in units of millipolarization (mP). The equilibrium dissociation constant (K_D) is shown for the WT LRE. Each data point is a mean of three experiments and the error bars represent the standard deviation. The WT LRE data plotted in both graphs is the same.

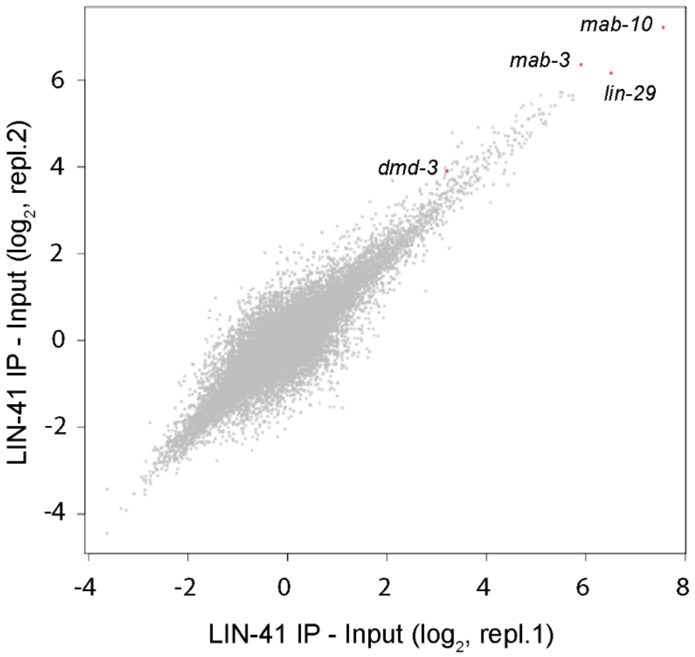

Supplementary Figure 4: CeLIN41 associated RNAs. Related to Fig. 7

The plot shows enrichment of transcripts in CeLIN41 immunoprecipitates (IP) versus inputs in two biologically independent experiments. Known somatic mRNA targets of CeLIN41 are marked in red and labeled.

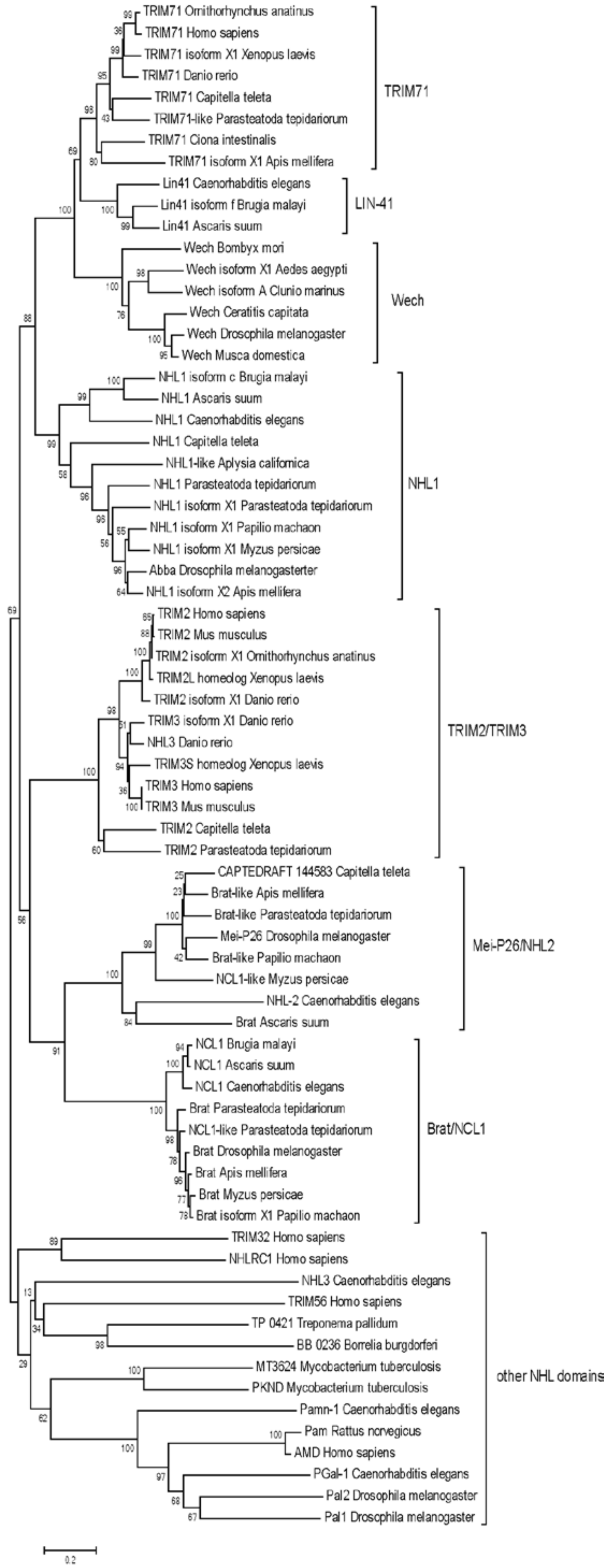
TRIM71 Ornithorhynchus anatinus
TRIM71 Homo sapiens
TRIM71 isoform X1 Xenopus laevis
TRIM71 Danio rerio
TRIM71 Capitella teleta
TRIM71-like Parasteatoda tepidariorum
TRIM71 Ciona intestinalis
TRIM71 isoform X1 Apis mellifera
TRIM71
Lin41 Caenorhabditis elegans
Lin41 isoform f Brugia malayi
Lin41 Ascaris suum
LIN-41
Wech Bombyx mori
Wech isoform X1 Aedes aegypti
Wech isoform A Clunio marinus
Wech Ceratitis capitata
Wech Drosophila melanogaster
Wech Musca domestica
Wech
NHL1 isoform c Brugia malayi
NHL1 Ascaris suum
NHL1 Caenorhabditis elegans
NHL1 Capitella teleta
NHL1-like Aplysia californica
NHL1 Parasteatoda tepidariorum
NHL1 isoform X1 Parasteatoda tepidariorum
NHL1 isoform X1 Papilio machaon
NHL1 isoform X1 Myzus persicae
Abba Drosophila melanogasterter
NHL1 isoform X2 Apis mellifera
NHL1
TRIM2 Homo sapiens
TRIM2 Mus musculus
TRIM2 isoform X1 Ornithorhynchus anatinus
TRIM2L homeolog Xenopus laevis
TRIM2 isoform X1 Danio rerio
TRIM3 isoform X1 Danio rerio
NHL3 Danio rerio
TRIM3S homeolog Xenopus laevis
TRIM3 Homo sapiens
TRIM3 Mus musculus
TRIM2 Capitella teleta
TRIM2 Parasteatoda tepidariorum
TRIM2/TRIM3
CAPTEDRAFT 144583 Capitella teleta
Brat-like Apis mellifera
Brat-like Parasteatoda tepidariorum
Mei-P26 Drosophila melanogaster
Brat-like Papilio machaon
NCL1-like Myzus persicae
NHL-2 Caenorhabditis elegans
Brat Ascaris suum
Mei-P26/NHL2
NCL1 Brugia malayi
NCL1 Ascaris suum
NCL1 Caenorhabditis elegans
Brat Parasteatoda tepidariorum
NCL1-like Parasteatoda tepidariorum
Brat Drosophila melanogaster
Brat Apis mellifera
Brat Myzus persicae
Brat isoform X1 Papilio machaon
Brat/NCL1
TRIM32 Homo sapiens
NHLRC1 Homo sapiens
NHL3 Caenorhabditis elegans
TRIM56 Homo sapiens
TP 0421 Treponema pallidum
BB 0236 Borrelia burgdorferi
MT3624 Mycobacterium tuberculosis
PKND Mycobacterium tuberculosis
Pamn-1 Caenorhabditis elegans
Pam Rattus norvegicus
AMD Homo sapiens
PGal-1 Caenorhabditis elegans
Pal2 Drosophila melanogaster
Pal1 Drosophila melanogaster
other NHL domains
0.2

Supplementary Figure 5: Molecular phylogeny analysis of NHL domains of various NHL domain-containing proteins. Related to Fig. 9

The evolutionary history was inferred using the Minimum Evolution method and the optimal tree is shown in Fig. 9. The percentage of replicate trees in which the associated taxa clustered together in the bootstrap test (1000 replicates) are shown next to the branches. The tree is drawn to scale, with branch lengths in the same units as evolutionary distances used to build the phylogenetic tree. The evolutionary distances were computed using the Poisson correction method and are in the units of the number of amino acid substitutions per site. Each subgroup has been named as shown in the compressed tree in Fig. 9.

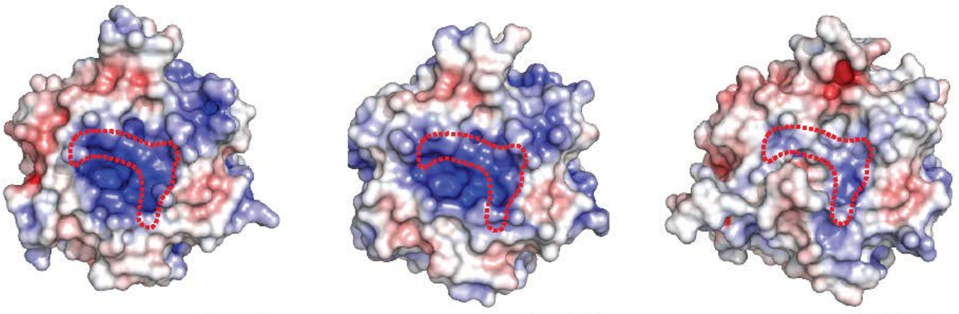

Supplementary Figure 6: Structural basis of RNA recognition by LIN41 orthologs. Related to Fig. 9

Homology models of CeLIN41, HsLIN41, and DmWech are shown in a surface mode, with their respective electrostatic surface potentials (scale as in Fig. 8). The approximate footprint of the RNA stem loop on the protein surface is shown as a dotted red line.

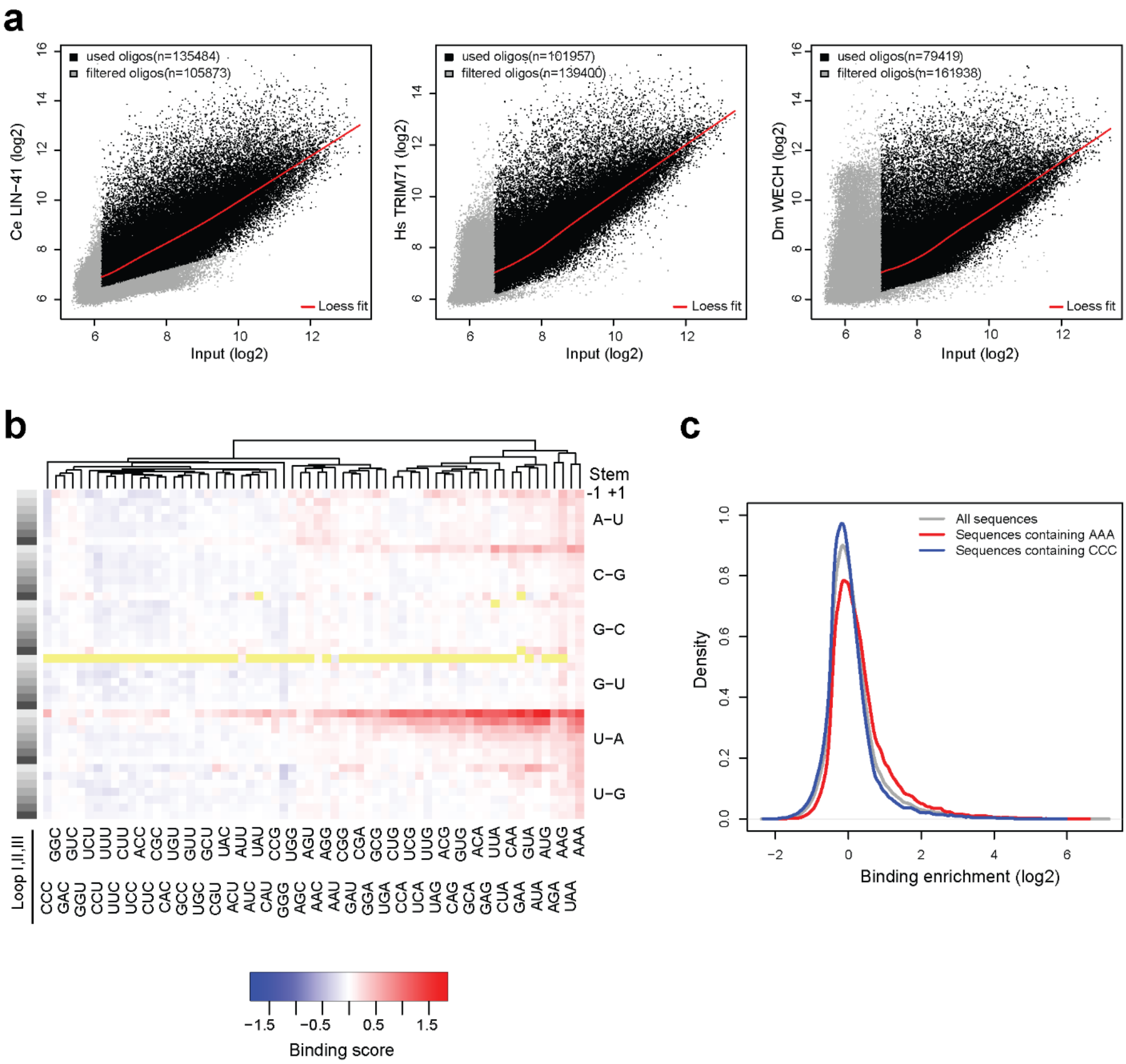
a
used oligos(n=135484)
filtered oligos(n=105873)
Ce LIN-41 (log2)
Loess fit
Input (log2)
used oligos(n=101957)
filtered oligos(n=139400)
Hs TRIM71 (log2)
used oligos(n=79419)
filtered oligos(n=161938)
Dm WECH (log2)
b
Stem
-1 +1
A–U
C–G
G–C
G–U
U–A
U–G
Loop I,II,III
Binding score
c
All sequences
Sequences containing AAA
Sequences containing CCC
Density
Binding enrichment (log2)

Supplementary Figure 7: Analysis of RNAcompete data for CeLIN41, HsLIN41 and DmWech. Related to methods.

(**a**) Grey dots indicate the oligonucleotide sequences that were removed from the analysis due to possible technical artifacts. These include: 1. The oligonucleotide sequences that showed a median intensity lower than 6 (log2 space) over the full set of 244 experiments (hugheslab.ccbr.utoronto.ca/supplementary-data/RNAcompete_eukarya/raw_data.txt.gz) as their intensity is less reliable. 2. The oligonucleotide sequences that were depleted in input. 3. For HsLIN41 and DmWech, oligonucleotides that were enriched in pull-down but showed very low intensity in input.

(**b**) CeLIN41 response element before setting a cut-off for enrichment values.

(**c**) RNAcompete enrichments for all sequences and sequences containing an AAA or a CCC. Sequences containing an AAA showed a slight shift in the distribution present at even low enrichment values (<1). CCC showed the opposite trend.

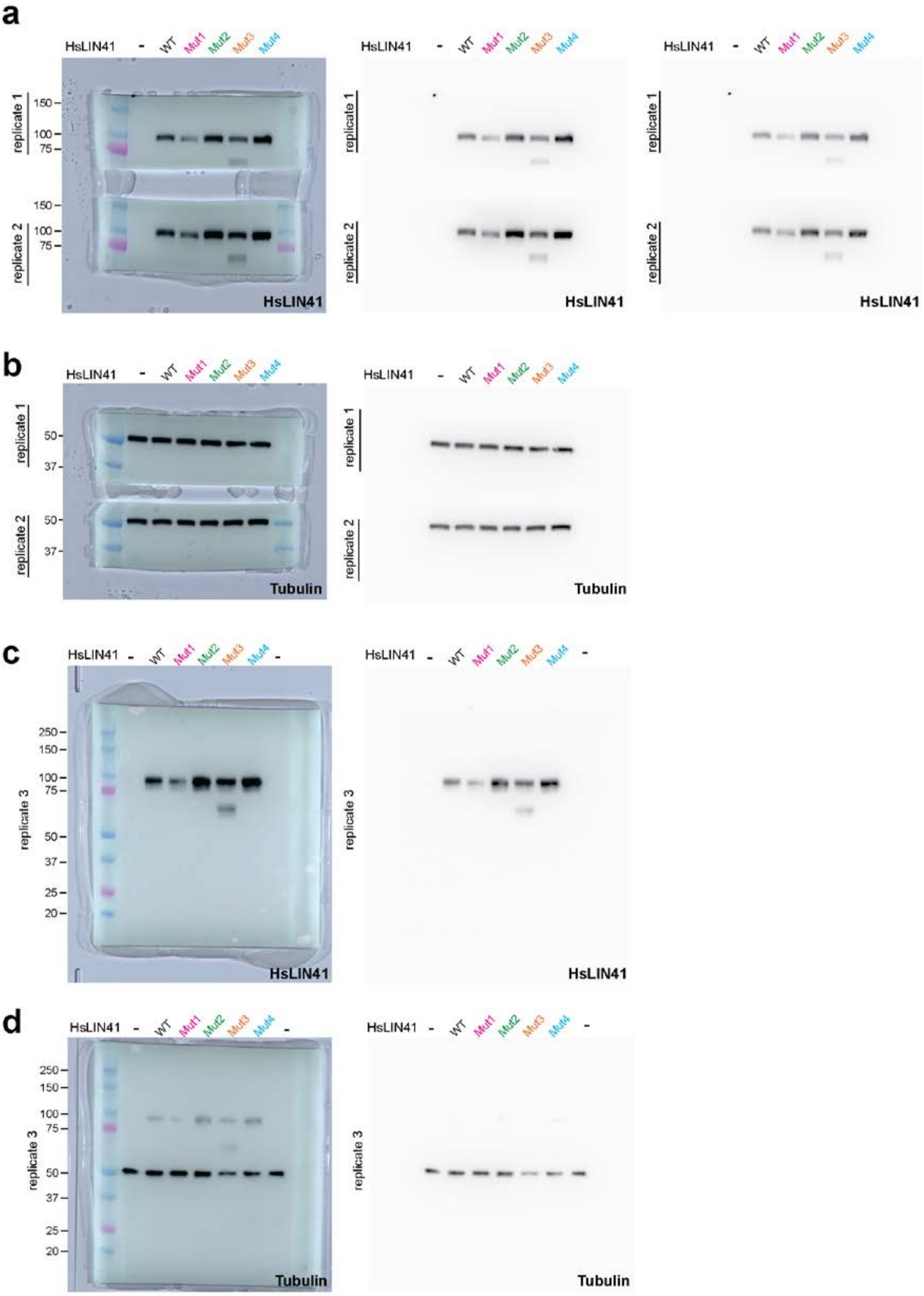
a
HsLIN41
-
WT
Mut1
Mut2
Mut3
Mut4
replicate 1
replicate 2
150
100
75
HsLIN41
b
50
37
Tubulin
c
replicate 3
250
150
100
75
50
37
25
20
HsLIN41
d
Tubulin

Supplementary Figure 8: Uncropped western blots with markers. Related to Supplementary Fig. 2g.

(**a**) Left: Two gels showing HsLIN41 levels, from independent experiments (replicates 1 and 2), with different exposures to the right. The middle exposure is shown for replicate 1, and the right exposure for replicate 2 in Supplementary Fig. 2g.

(**b**) Tubulin levels from replicates 1 and 2, corresponding to **a**, shown in Supplementary Fig. 2g.

(**c**) Left: The gel from replicate 3, showing HsLIN41 levels. The exposure on the right is shown in Supplementary Fig. 2g.

(**d**) Tubulin levels from replicate 3, corresponding to **c**, shown in Supplementary Fig. 2g.

Supplementary Table 1: Probes for gel-shifts. Related to Fig. 2 and Supplementary Fig. 1.

mab-10 part 2	uaaauucccgcccccuucgcucggagucauauaauuuccuucucuuccuaucacuuuuuugucuaguuuucaaaaaacccc<br>ccauuuuuccggcgguuuuucaaaaaccaaugccaagcucucauauaaugggagucuaaacacaguucgguuccaaaacca<br>acgcccacggccccgcccccaauuagauaauuuaaaua
mab-10 part 2 mut	uaaauucccgcccccuucgcucggagucauauaauuuccuucucuuccuaucacuuuuuugucuaguuuucaaaaaacccc<br>ccauuuuuccggcgCuuuuucaaaaaccaaugccaagcucuGauauaaugggagucuaaacacaguucgguuccaaaacca<br>acgcccacggccccgcccccaauuagauaauuuaaaua
mab-10 part 2 mut-res	uaaauucccgcccccuucgcucggagucauauaauuuccuucucuuccuaucacuuuuuugucuaguuuucaaaaaacccc<br>ccauuuuuccggcgCuuuuucaaaaaGcaaugccaagcucuGauauaauCggagucuaaacacaguucgguuccaaaacca<br>acgcccacggccccgcccccaauuagauaauuuaaaua
mab-10 part 4	Ucgcauccaaugcauugcaaguugauugcuuuuauaaauuuuugggucucgaagcgaaaagcccauaggauuuucacaaaa<br>auuuugcauuuaaugcagugaaaauucaaaaaauucaaagauuuuucugaauagaucaaauuuucaaauuuuccggucauu<br>cgaaauggccuaauuugcuccaaaaauuugaauaaucc
mab-10 part 4 mut	ucgGauccaaugcauugcaaguugauugcuuuuauaaauuuuugggucucgaagcgaaaagcccauaggauuuucacaaaa<br>auuuugGauuuaaugcagugaaaauucaaaaaauucaaagauuuuucugaauagaucaaauuuucaaauuuuccgguGauu<br>cgaaauggccuaauuugcuccaaaaauuugaauaaucc
mab-10 part 4 mut-res	ucgGauccaauCcauugcaaguugauugcuuuuauaaauuuuugggucucgaagcgaaaagcccauaggauuuucacaaaa<br>auuuugGauuuaauCcagugaaaauucaaaaaauucaaagauuuuucugaauagaucaaauuuucaaauuuuccgguGauu<br>cgaaauCgccuaauuugcuccaaaaauuugaauaaucc
mab-10 condensed 3’UTR	ucuaguuuucaaaaaaccccccauuuuuccggcgguuuuucaaaaaccaaugccaagcucucauauaaugggagucuaaac<br>acaguucgguuccaaaacugaaaaacuucaauuucgcauccaaugcauugcaaguugauugcuuuucacaaaaauuuugca<br>uuuaaugcagugaaaauuuuccggucauucgaaauggccuaauuugcuccaaaaa
mab-10 condensed 3’UTR mut	ucuaguuuucaaaaaaccccccauuuuuccggcgCuuuuucaaaaaccaaugccaagcucuGauauaaugggagucuaaac<br>acaguucgguuccaaaacugaaaaacuucaauuucgGauccaaugcauugcaaguugauugcuuuucacaaaaauuuugGa<br>uuuaaugcagugaaaauuuuccgguGauucgaaauggccuaauuugcuccaaaaa
mab-10 condensed 3’UTR mut-res	ucuaguuuucaaaaaaccccccauuuuuccggcgCuuuuucaaaaaGcaaugccaagcucuGauauaauCggagucuaaac<br>acaguucgguuccaaaacugaaaaacuucaauuucgGauccaauCcauugcaaguugauugcuuuucacaaaaauuuugGa<br>uuuaauCcagugaaaauuuuccgguGauucgaaauCgccuaauuugcuccaaaaa
lin-29A	guuccgaaccaauucgccuagcgaaccuuggaguccaacuccguuuuuacucuuuucugaaaagagccuacuaaauauugg<br>aacuauaaucaaacuauucgaucaaaucgacacaccuacuagauaucaaauaucuaaauacuggaaccaaggcuuggguaac<br>cugaaacugugcgaauuuggagcagaaauauu
lin-29A mut2	guuccgaaccaauucgccuagcgaaccuuggaCuccaacuccguuuuuacucuuuucugaaaagagccuacuaaauauugg<br>aacuauaaucaaacuauucCaucaaaucgacacaccuacuagauaucaaauaucuaaauacuggaaccaaggcuuggguaac<br>cugaaacugugcgaauuuggagcagaaauauu
lin-29A mut2-res	guuccgaaccaauucgccuagcgaaccuuggaCuccaaGuccguuuuuacucuuuucugaaaagagccuacuaaauauugg<br>aacuauaaucaaacuauucCaucaaauGgacacaccuacuagauaucaaauaucuaaauacuggaaccaaggcuuggguaac<br>cugaaacugugcgaauuuggagcagaaauauu
lin-29A mut1	guuccgaaccaauucgccuagcgaaccuuggaCuccaacuccguuuuuacuGuuuucugaaaagagccuacuaaauauugg<br>aacuauaaucaaacuauuGGaucaaaucgacacaccuacuaCauaucaaauaucuaaauacuggaaccaaggcuuggguaac<br>cugaaacugugcgaauuuggagcagaaauauu
lin-29A mut1-res	guuccgaaccaauucgccuagcgaaccuuggaCuccaaGuccguuuuuacuGuuuucugaaaaCagccuacuaaauauugg<br>aacuauaaucaaacuauuGGaucaaauCCacacaccuacuaCauaucaaauauGuaauacuggaaccaaggcuuggguaac<br>cugaaacugugcgaauuuggagcagaaauauu

Mutations are marked in red.

Supplementary Table 2: Crystallographic data collection and refinement statistics. Related to Fig. 4-5 and Supplementary Fig. 2

	D. rerio LIN41 filamin-NHL unbound[a]	*D. rerio* LIN41 filamin-NHL *lin-29A* complex	*D. rerio* LIN41 filamin-NHL *mab-10* complex
Data collection			
Space group	$P2_12_12_1$	$P3_121$	$P3_121$
Unit cell dimensions			
a, *b*, *c* (Å)	65.80, 90.59, 131.05	101.85, 101.85, 109.89	101.49, 101.49, 108.94
α, β, γ (°)	90.0, 90.0, 90.0	90.0, 90.0, 120.0	90.0, 90.0, 120.0
Resolution range (Å)[b]	50.00-2.60 (2.67-2.60)	50.00-1.90 (1.95-1.90)	50.00-2.35 (2.41-2.35)
Wavelength (Å)	1.260	1.000	1.000
Completeness (%)[b]	91.6 (86.8)	99.4 (92.1)	99.4 (92.2)
Redundancy[b]	1.9 (1.8)	7.7 (7.7)	16.5 (15.4)
R_{sym}[b]	0.124 (0.491)	0.042 (2.172)	0.124 (2.468)
$I/\sigma(I)$[b]	5.28 (1.48)	20.24 (0.81)	16.02 (0.99)
CC (1/2) (%)[b]	98.7 (77.9)	99.9 (38.1)	99.9 (42.9)
Unique reflections	42647	51941	27367
Refinement			
R_{work}	0.182	0.158	0.172
R_{free}	0.244	0.180	0.211
Resolution range (Å)	46.43 - 2.60	46.64 – 1.90	46.30 – 2.35
Reflections (all)	23630	51933	27359
Reflections (test set)	1182 (5%)	2596 (5%)	1368 (5%)
**Number of atoms**			
Overall	6217	3541	3384
Protein	6002	3024	3013
RNA	-	272	271
Solvent	215	245	100
***B*-Factors** (Å^2)			
Overall	30.6	61.4	67.1
Protein	31.0	61.3	67.1
RNA	-	58.9	68.3
Solvent	19.2	63.7	66.7
**RMS Deviations**			
Bond lengths (Å)	0.01	0.007	0.007
Bond angles (°)	1.20	0.792	0.842
**Ramachandran plot**			
Allowed (%)	98.3	100	100
Outliers (%)	1.7	0	0

[a]Data collection statistics is reported for unmerged Friedel pairs

[b]Values in parentheses refer to the highest resolution shell

Supplementary Table 3: LRE categories. Related to Fig. 7.

RNA motif Variant	1/-1	I,II,III	Pairing Probability Bin	Score	Level
UAUGA:7	U-A	AUG	7	1.84441	Strong
UAUAA:7	U-A	AUA	7	1.63461	Strong
UGAAA:7	U-A	GAA	7	1.45818	Strong
UCAAA:7	U-A	CAA	7	1.2545	Strong
UACAA:7	U-A	ACA	7	1.22135	Strong
UAAAA:7	U-A	AAA	7	1.16811	Strong
UGCAA:7	U-A	GCA	7	1.11358	Strong
UGUAA:7	U-A	GUA	7	1.06096	Strong
UCUAA:7	U-A	CUA	7	1.05241	Strong
UAAGA:7	U-A	AAG	7	0.9707	Strong
UUUAA:7	U-A	UUA	7	0.96786	Strong
UCAGA:7	U-A	CAG	7	0.94788	Strong
UAUAA:6	U-A	AUA	6	0.9321	Strong
UGUGA:7	U-A	GUG	7	0.8991	Medium
UUUGA:7	U-A	UUG	7	0.89742	Medium
UUAGA:7	U-A	UAG	7	0.87829	Medium
UGAGA:7	U-A	GAG	7	0.86413	Medium
UGUAA:6	U-A	GUA	6	0.83564	Medium
UUAAA:7	U-A	UAA	7	0.83551	Medium
UUCAA:7	U-A	UCA	7	0.83404	Medium
UAUGA:6	U-A	AUG	6	0.81587	Medium
UACGA:7	U-A	ACG	7	0.80056	Medium
UCCAA:7	U-A	CCA	7	0.77021	Medium
UAAAA:6	U-A	AAA	6	0.76223	Medium
UCUGA:7	U-A	CUG	7	0.74896	Medium
UAGAA:7	U-A	AGA	7	0.73576	Medium
UUCGA:7	U-A	UCG	7	0.73005	Medium
UUUAA:6	U-A	UUA	6	0.72904	Medium
UGAGA:6	U-A	GAG	6	0.71494	Medium
UCUAA:6	U-A	CUA	6	0.68443	Medium
UGAUA:7	U-A	GAU	7	0.6844	Medium
UACAA:6	U-A	ACA	6	0.67449	Medium
UUAAA:6	U-A	UAA	6	0.61226	Medium
UUGAA:7	U-A	UGA	7	0.60406	Medium
UCGAA:7	U-A	CGA	7	0.5824	Medium
UCGGA:7	U-A	CGG	7	0.57692	Medium
UGAAA:6	U-A	GAA	6	0.56925	Medium
UAAAA:5	U-A	AAA	5	0.54223	Medium
UCAAA:6	U-A	CAA	6	0.52941	Medium
CUUAG:7	C-G	UUA	7	0.49976	Medium
UGCGA:7	U-A	GCG	7	0.4898	Medium
UGUAA:5	U-A	GUA	5	0.45918	Medium

UGUGA:6	U-A	GUG	6	0.45877	Medium
UCCCA:7	U-A	CCC	7	0.44002	Weak
UUCAA:6	U-A	UCA	6	0.43203	Weak
CUAAG:7	C-G	UAA	7	0.42949	Weak
UUCGA:6	U-A	UCG	6	0.4135	Weak
CAUAG:7	C-G	AUA	7	0.39446	Weak
UACUA:7	U-A	ACU	7	0.39307	Weak
UUAAA:5	U-A	UAA	5	0.39234	Weak
UCAGA:6	U-A	CAG	6	0.36546	Weak
UUAGA:6	U-A	UAG	6	0.35639	Weak
UUAUA:7	U-A	UAU	7	0.35141	Weak
UACGA:6	U-A	ACG	6	0.35003	Weak
CAAAG:7	C-G	AAA	7	0.34865	Weak
UGGAA:7	U-A	GGA	7	0.34131	Weak
UCCAA:6	U-A	CCA	6	0.32522	Weak
UAUAA:5	U-A	AUA	5	0.32497	Weak
UUUGA:6	U-A	UUG	6	0.3208	Weak
UACAA:5	U-A	ACA	5	0.3126	Weak
UUCAA:5	U-A	UCA	5	0.31073	Weak
CCAAG:7	C-G	CAA	7	0.30293	Weak
UAAUA:7	U-A	AAU	7	0.29526	Weak
UUUAA:5	U-A	UUA	5	0.28141	Weak
UGAAA:5	U-A	GAA	5	0.27765	Weak
UCUAA:5	U-A	CUA	5	0.27139	Weak
UGCGA:6	U-A	GCG	6	0.26833	Weak
UUAAA:4	U-A	UAA	4	0.25887	Weak
CGUAG:7	C-G	GUA	7	0.25801	Weak
UCAUA:7	U-A	CAU	7	0.25633	Weak
UUUGA:5	U-A	UUG	5	0.2535	Weak
UUGAA:6	U-A	UGA	6	0.25261	Weak
CCUAG:7	C-G	CUA	7	0.24647	Weak
UCAAA:5	U-A	CAA	5	0.24372	Weak
UAACA:6	U-A	AAC	6	0.2429	Weak
UUCUA:7	U-A	UCU	7	0.241	Weak
UCUGA:6	U-A	CUG	6	0.23796	Weak
UCGCA:7	U-A	CGC	7	0.23787	Weak
UUAGA:5	U-A	UAG	5	0.23348	Weak
CGAAG:7	C-G	GAA	7	0.23094	Weak
UGUGA:5	U-A	GUG	5	0.23041	Weak
UAAGA:6	U-A	AAG	6	0.22696	Weak
CAUGG:7	C-G	AUG	7	0.2242	Minimal
UAAGA:5	U-A	AAG	5	0.22194	Minimal
UGCAA:6	U-A	GCA	6	0.2187	Minimal
UAGAA:6	U-A	AGA	6	0.2184	Minimal

UAUGA:5	U-A	AUG	5	0.213	Minimal
UCUCA:7	U-A	CUC	7	0.20311	Minimal
UGCAA:5	U-A	GCA	5	0.20054	Minimal
UAACA:7	U-A	AAC	7	0.19932	Minimal
UCCGA:7	U-A	CCG	7	0.19771	Minimal
UAUCA:7	U-A	AUC	7	0.19687	Minimal
UCUAA:4	U-A	CUA	4	0.19199	Minimal
UAUUA:7	U-A	AUU	7	0.18267	Minimal
UAUAA:4	U-A	AUA	4	0.17849	Minimal
UAGUA:7	U-A	AGU	7	0.17612	Minimal
UCAGA:5	U-A	CAG	5	0.17534	Minimal
CUAGG:7	C-G	UAG	7	0.17524	Minimal
UGUAA:4	U-A	GUA	4	0.17498	Minimal
UCUGA:5	U-A	CUG	5	0.17405	Minimal
UAGUA:5	U-A	AGU	5	0.16971	Minimal
UCACA:7	U-A	CAC	7	0.16886	Minimal
UAGUA:6	U-A	AGU	6	0.16481	Minimal
UGUGA:4	U-A	GUG	4	0.16412	Minimal
UAGGA:7	U-A	AGG	7	0.16237	Minimal
CUUGG:7	C-G	UUG	7	0.1543	Minimal
UCGAA:6	U-A	CGA	6	0.1542	Minimal
UGAGA:5	U-A	GAG	5	0.14935	Minimal
CACAG:7	C-G	ACA	7	0.14653	Minimal
UUGAA:5	U-A	UGA	5	0.14441	Minimal
UGCGA:5	U-A	GCG	5	0.1441	Minimal
UGGGA:7	U-A	GGG	7	0.14338	Minimal
UAGGA:6	U-A	AGG	6	0.13911	Minimal
UGGAA:6	U-A	GGA	6	0.13808	Minimal
CAAGG:7	C-G	AAG	7	0.13713	Minimal
CAGAG:7	C-G	AGA	7	0.13602	Minimal
CGUGG:7	C-G	GUG	7	0.12768	Minimal
UGAGA:4	U-A	GAG	4	0.12282	Minimal
CUCAG:7	C-G	UCA	7	0.11913	Minimal
UCUCA:6	U-A	CUC	6	0.1165	Minimal
CCGAG:7	C-G	CGA	7	0.11584	Minimal
UCACA:5	U-A	CAC	5	0.11195	Minimal
UACCA:7	U-A	ACC	7	0.10635	Minimal
UAAAA:4	U-A	AAA	4	0.10354	Minimal
UACAA:4	U-A	ACA	4	0.10163	Minimal
UUCGA:5	U-A	UCG	5	0.10112	Minimal
UGAUA:6	U-A	GAU	6	0.10001	Minimal

Supplementary Table 4: ***C. elegans*** **strains. Related to Methods**

Experimental Models: *C. elegans* Strains		
Strain	Source	Identifier
Wild-type	CGC	N2
EG6699, *xeSi147[Pdpy-30::lin-29A 5'UTR exon 1::gfp(pest)/h2b::unc-54 3'UTR, unc-119(+)]* II	Aeschimann et al., 2017[1]	HW1619
EG6699, *xeSi313[Pdpy-30::lin-29A 5'UTR exon 1 mut2::gfp(pest)/h2b::unc-54 3'UTR, unc-119(+)]* II	This study	HW1994
EG6699, *xeSi314[Pdpy-30::lin-29A 5'UTR exon 1 mut2-res::gfp(pest)/h2b::unc-54 3'UTR, unc-119(+)]* II	This study	HW1995
EG6699, *xeSi318[Plin-29A(act-1 5'UTR exon)::gfp(pest)/h2b::unc-54 3'UTR(mab-10 condensed 3'UTR), unc-119(+)]* II	This study	HW1999
EG6699, *rrrSi473[Plin-29A(act-1 5'UTR exon)::gfp(pest)/h2b::unc-54 3'UTR(mab-10 condensed 3'UTR mut), unc-119(+)]* II	This study	RAF2004
EG6699, *rrrSi474[Plin-29A(act-1 5'UTR exon)::gfp(pest)/h2b::unc-54 3'UTR(mab-10 condensed 3'UTR mut-res), unc-119(+)]* II	This study	RAF2005
lin-41(rrr3) (I); rrrSi367 [Plin-41::FLAG-GFP-TEV-LIN-41::lin-41 full-length 3'UTR; unc-119(+)] (II)	Tocchini et al., 2014[2]	RAF1587

Supplementary Table 5: RNA sequences used for fluorescence polarization. Related to Methods

Name	Length	Sequence	Description
L1	13	GGAGUCCAACUCC	lin-29A 5'UTR SL I
Ctrl	13	GCUGACCAACUCC	a stem-loop with 5 nucleotides in the loop
L1.1 (Mut1)	13	GGAGUCCUACUCC	Loop III (A->U) mutation in L1
L1.2 (Mut2)	13	GGAGUCCCACUCC	Loop III (A->C) mutation in L1
L1.3 (Mut3)	13	GGAGUCCGACUCC	Loop III (A->G) mutation in L1
L1.4 (Mut4)	13	GGAGACCAUCUCC	Stem -1/+1 (U-A->A-U) mutation in L1
L1.5 (Mut5)	13	GGAGCCCAGCUCC	Stem -1/+1 (U-A->C-G) mutation in L1
L1.6 (Mut6)	13	GGAGGCCACCUCC	Stem -1/+1 (U-A->G-C) mutation in L1

Mutations are marked in red.

Supplementary Note 1: RNA Secondary structure models.

The predicted secondary structures for *lin-29A* 5'UTR, parts of *mab-10* 3'UTR and the condensed *mab-10* 3'UTR are shown in dot-bracket notation. Several models are shown for each sequence and their minimum free energies are indicated. Sequence regions that fold into tri-loop SLs with a U-A base pair at stem position 1 are highlighted in green. Other tri-loop SLs are highlighted in yellow. The loop nucleotides are shown in red. Alternative secondary structures of these regions are highlighted in gray. The yellow SLs in part 2 and part 3 of the *mab-10* 3'UTR arise from overlapping sequences and hence will never form simultaneously in the context of whole *mab-10* 3'UTR.

C. elegans mab-10 3'UTR

Part 2 - nucleotides 151 – 350

```
                                                                                                                SLI                    SLII
UAAAUUCCCGCCCCCUUCGCUCGGAGUCAUAUAAUUUCCUUCUCUUCCUAUCACUUUUUUGUCUAGUUUUCAAAAAACCCCCCAUUUUUCCGGCGGUUUUUCAAAAACCAAUGCCAAGCUCUCAUAUAAUGGGAGUCUAAACACAGUUCGGUUCCAAAACCAACGCCCACGGCCCCGCCCCAAUUAGAUAAUUUAAAUA
1. >ENERGY = -32.6
...(((((.((.......))..)))))...............................((((((((((((..(((((((.((.........)).)))))))..)))))........((((((((...)))))))).........(((.((((....)))))))(((...)))............))))))).........
2. >ENERGY = -32.4
...(((((.((.......))..)))))...............................((((((((((.......................(((((((((...))))))...))).(((((((((...))))))))...........(((.((((....)))))))(((...))).........))))))))))........
3. >ENERGY = -30.0
......................(((((.(((.................))).))))).((((((((((((..(((((((.((.........)).)))))))..)))))........((((((((...))))))))).........(((.((((....)))))))(((...)))............))))))).........
4. >ENERGY = -29.6
...(((((.((.......))..))))).....................((((..(((((((.........)))))))..............(((((((((...))))))...))).(((((((((...)))))))..)).......(((.((((....)))))))(((...)))..............)))).........
```

Part 3 - nucleotides 301 – 500

```
                                                                                                                                                      SLIII
GUUCCAAAACCAACGCCCACGGCCCCGCCCCCAAUUAGAUAAUUUAAAUAUAAUUUUUUUUUCCAUUUUCCAAUUUUUUAAAAAUAUUUAAUAUGUUUGUCUUGCUCUAUUGAGAAAGAAAUUUGAAUUCAAAACUGAAAAACUUCAAUUUCGCAUCCAAUGCAUUGCAAGUUGAUUGCUUUUAUAAAUUUUUGGGUCUC
>   ENERGY = -27.3
..............(((((.(((...))).......(((((..((((((((................................))))))))..))))).((((.(((....))).))))((.(((((((((....))))....))))).)).((((...))))...((((.....)))).............)))))...
>   ENERGY = -26.9
..............(((((.(((...))).......(((((..((((((((................................))))))))..))))).((((.(((....))).))))((.(((((((((....))))....))))).)).(((..((((((...)))..)))..))).............)))))...
>   ENERGY = -26.8
..............(((((.(((...)))......((((((..((((((((................................))))))))..)))))).(((((..(((((.((..((((((.(((((((....))))....)))))))))...)))))))....))))).....................)))))...
>   ENERGY = -26.4
..............(((((.(((...))).......(((((..((((((((................................))))))))..))))).((((.(((....))).))))........((((....)))).(((((((((...((((...)))))))).))))).................)))))...
>   ENERGY = -26.3
..............(((...)))......(((((.((((((..((((((((................................))))))))..))))))((((.(((....))).)))).(.(((((((((....))))....))))).)..((((...))))...((((.....))))............)))))....
>   ENERGY = -26.2
..............(((((.(((...))).......(((((..((((((((................................))))))))..))))).((((.(((....))).))))..((((....))))..(((((....(((((((.(((..........))))))))))....))))).........)))))...
>   ENERGY = -25.8
..............(((((.(((...))).......(((((..((((((((................................))))))))..))))).((((.(((....))).))))............(((.((((....)))).))).((((...))))...((((.....)))).............)))))...
>   ENERGY = -25.7
..(((((((.....(((...))).............(((((..((((((((................................))))))))..))))).((((.(((....))).))))((.(((((((((....))))....))))).)).((((...))))...((((.....))))..........)))))))....
>   ENERGY = -25.5
..............(((((.(((...)))....................................................((((((.....)))))).((((.(((....))).))))((.(((((((((....))))....))))).)).((((...))))...((((.....)))).............)))))...
>   ENERGY = -24.7
..............(((((.(((...)))...((((.((.(((..(((...........)))..))).)).))))......((((((.....)))))).((((.(((....))).))))((.(((((((((....))))....))))).)).((((...))))...((((.....)))).............)))))...
>   ENERGY = -24.6
..............(((...)))......(((((..(((((..((((((((................................))))))))..))))).((((.(((....))).))))(((((...........(((((....(((((((.(((..........))))))))))....)))))))))).)))))....
```

C. elegans mab-10 3'UTR

Part 4 - nucleotides 451 – 650

```
  SLIII                                                                                     SLIV                                                                        SLV
UCGCAUCCAAUGCAUUGCAAGUUGAUUGCUUUUAUAAAUUUUUGGGUCUCGAAGCGAAAAGCCCAUAGGAUUUUCACAAAAAUUUUGCAUUUAAUGCAGUGAAAAUUCAAAAAAUUCAAAGAUUUUUCUGAAUAGAUCAAAUUUUCAAAUUUUCCGGUCAUUCGAAAUGGCCUAAUUUGCUCCAAAAAUUUGAAUAAUCC

1.   >ENERGY = -41.5
..((((...))))...((((.....)))).....(((((((((((.......(((((((.((.((((.((((((((((........(((((...)))))))))))))))..((((((..(((((((.(((....)))..)))))))..)))))).(((....)))..)))).))..)))))))))))))))))........
2.   >ENERGY = -41.5
..(((..((((((...)))..)))..))).....(((((((((((.......(((((((.((.((((.((((((((((........(((((...)))))))))))))))..((((((..(((((((.(((....)))..)))))))..)))))).(((....)))..)))).))..)))))))))))))))))........
3.   >ENERGY = -41.2
..(((..((((((...)))..)))..))).....(((((((((((.......(((((((.((.((((.((((((((((........(((((...))))))))))))))).(((((((....))))))).(((((.(((((((((....)))))...)))))))))..)))).))..)))))))))))))))))........
4.   >ENERGY = -40.2
..(((..((((((...)))..)))..)))..................(((((.(((...)))...)))))..((((((((((........(((((...))))))))))))))).(((((((....)))))))..........((((((((((((((((....(((((((...))))))).)))))).....))))))))).......
5.   >ENERGY = -39.9
..((((...))))...(((((((...................(((((.(((...)))...)))))..((((((((((........(((((...)))))))))))))))..((((((..(((((((.(((....)))..)))))))..))))))..(((((((...))))))).)))))))....................
6.   >ENERGY = -38.6
..((((..(((((...((((.....)))).............(((((.(((...)))...))))).((((((((....))))))))))))))..))))........((((((((((((....))))))).)))))...(((((((((((((((....(((((((...))))))).)))))).....)))))))))).......
7.   >ENERGY = -38.6
..((((...))))...((((.....)))).((((.(((((((.((((.(((...)))...))))...((((((((((........(((((...)))))))))))))))..........)))))))...))))....(((((((((((((((....(((((((...))))))).)))))).....)))))))))).......
8.   >ENERGY = -37.8
............((((((..((((.(((......(((((((((((((.(((...)))...))))).))))))).............))).))))))))))))...((((((((((((....))))))).)))))...(((((((((((((((....(((((((...))))))).)))))).....)))))))))).......
9.   >ENERGY = -37.7
..((((...)))).(((.(((((((((..(((...(((((((.((((.(((...)))...))))...((((((((((........(((((...)))))))))))))))..........)))))))....)))..)))).))))).))).......(((((((...))))))).(((((.......)))))..........
10.  >ENERGY = -37.6
..(((..((((((...)))..)))..))).....(((((((((((..........(((((.((....)).))))).........(((((...))))).(((..((((((((((((....))))))).)))))...)))......((((((....(((((((...))))))).)))))).))))))))))).........
11.  >ENERGY = -37.5
(((.((((((......((((.....))))............)))))).))).((.....)).....((((((((((........(((((...))))))))))))))).(((((((....)))))))..........((((((((((((((((....(((((((...))))))).)))))).....)))))))))).......
12.  >ENERGY = -37.4
..(((..((((((...)))..)))..))).....((((((..(((((.(((...)))...)))))..((((((((((........(((((...)))))))))))))))..((((((..(((((((.(((....)))..)))))))..))))))..(((((((...))))))).))))))..................
```

C. elegans mab-10 condensed 3’UTR (217 nucleotides)

SLI SLII SLIII SLIV SLV

```
UCUAGUUUUCAAAAAACCCCCAUUUUUCCGGCGGUUUUUCAAAAACCAAUGCCAAGCUCUCAUAUAAUGGGAGUCUAAACACAGUUCGGUUCCAAAACUGAAAAACUUCAAUUUCGCAUCCAAUGCAUUGCAAGUUGAUUGCUUUUCACAAAAAUUUUGCAUUUAAUGCAGUGAAAAUUUUCCGGUCAUUCGAAAUGGC
CUAAUUUGCUCCAAAAA
>   ENERGY = -46.9
....(((((..(((((((.((.........)).)))))))..)))))....((((.((((((((...)))))))).........(((((((....))))))).(((((((((...((((...)))))))).)))))......(((((((........(((((...)))))))))))).((((.((....))))))))))
.................
>   ENERGY = -45.5
....(((((..(((((((.((.........)).)))))))..))))).(((((.(.((((((((...)))))))).........(((((((....)))))))....................)))))))(((((((......(((((((........(((((...))))))))))))).......(((((((...))))))
).)))))))........
>   ENERGY = -44.4
...((((((((..................(((((((((...))))))...))).((((((((...)))))))).)).......(((((((....))))))).(((((((((...((((...)))))))).)))))...................(((((...))))).))))))))....(((((((...))))))
)................
>   ENERGY = -44.3
....(((((..(((((((.((.........)).)))))))..)))))....(.((.((((((((...)))))))).........(((((((....)))))))............(((..((((((...)))..)))..))).((((((........(((((...)))))))))))).......(((((((...))))))
).....)))........
>   ENERGY = -43.5
....(((((..(((((((.((.........)).)))))))..))))).((((((.(((((((((...)))))))).)).......(((((((....))))))).(((((((((...((((...)))))))).)))))......(((((((........(((((...))))))))))))......)).)))).....(((
.......)))........
>   ENERGY = -43.4
....(((((..(((((((.((.........)).)))))))..)))))....((((.((((((((...)))))))).........(((((((....))))))).(((((((((...((((...)))))))).)))))..............((((((((.((((......)))))))))))).(((....))...))))
.................
>   ENERGY = -43.3
....(((((..(((((((.((.........)).)))))))..)))))....(.((.((((((((...)))))))).........(((((((....)))))))............((((..((((((..((((.....))))...............))))))..))))...(((....))).(((((((...))))))
).....)))........
```

C. elegans lin-29A 5’UTR exon 1 (194 nucleotides)

SL1 SLII SLIII SLIV

```
GUUCCGAACCAAUUCGCCUAGCGAACCUUGGAGUCCAACUCCGUUUUUACUCUUUUCUGAAAAGAGCCUACUAAAUAUUGGAACUAUAAUCAAACUAUUCGAUCAAAUCGACACACCUACUAGAUAUCAAAUAUCUAAUACUGGAACCAAGGCUUGGGUAACCUGAAACUGUGCGAAUUUGGAGCAGAAAUAUU
1. >   ENERGY = -41.5
(((((((....((((((.(((....((((((..((((............(((((((...)))))))((...........))................(((((...))))).......((.(((((((...))))))).)).)))).)))))).(((((...)))))..))).))))))))))))).........
2. >   ENERGY = -41.5
(((((((....((((((.(((....((((((..((((..((((..((((((((((...))))))).....))))...)))).................(((((...))))).......((.(((((((...))))))).)).)))).)))))).(((((...)))))..))).))))))))))))).........
3. >   ENERGY = -40.1
(((((((....((((((.(((.(((...(((((...))))))))))...(((((((...)))))))((...........)).................(((((...))))).........(((((((...))))))).((((.((.........)).))))........))).))))))))))))).........
4. >   ENERGY = -38.2
((((((((.....((((...)))).((((((..((((............(((((((...)))))))((...........)).................(((((...))))).......((.(((((((...))))))).)).)))).)))))).(((.(.............).))).))))))))).........
5. >   ENERGY = -38.1
(((((((....((((((.(((.(((...(((((...))))))))))...(((((((...)))))))((((.......((((..(((.............(((((...))))).......((.(((((((...))))))).)).)))..))))....))))..........))).))))))))))))).........
6. >   ENERGY = -37.9
((((((((((((((((...))))))..))))(((((((............(((((((...)))))))...........))))).)))..............................(((.((((.....(((((((...))))))).((((.((.........)).)))).........))))))).))))))))).........
```

Supplementary Note 2: Multiple sequence alignment of NHL domains of various NHL domain-containing proteins.

Alignment positions with a conserved residue in 60% or more of the proteins analyzed are highlighted in cyan.

```
BratDrosophilamelanogaster                    KMIYHCKF-GEFGVM-----EGQFTEPSGVAVNA-QNDIIVADTNN-HRIQIF-DKEGRFKFQFGECGK--------------------RDSQLLYPNRVAVVR-----NSGDIIVTERS
BratisoformX1Papiliomachaon                   KMIYHCKF-GEFGVM-----EGQFTEPSGVAVNA-QNDIIVADTNN-HRIQIF-DKEGRFKFQFGECGK--------------------RDGQLLYPNRVAVVR-----TSGDIIVTERS
BratApismellifera                             KMIYHCKF-GEFGVM-----EGQFTEPSGVAVNA-QNDIIVADTNN-HRIQIF-DKEGRFKFQFGECGK--------------------RDGQLLYPNRVAVVK-----TSGDIIVTERS
BratMyzuspersicae                             KMIYHCKF-GEFGAM-----EGQFTEPSGVAVNA-QNDIIVADTNN-HRIQIF-DKEGRFKFQFGECGK--------------------RDGQLLYPNRVAVVK-----PSGDIIVTERS
BratParasteatodatepidariorum                  KMIYHCKF-GEFGVM-----EGQFTEPSGVAVNA-QNDIIVADTNN-HRIQIF-DKEGRFKFQFGECGK--------------------RDGQLLYPNRVAVVK-----NSGDIIVTERS
NCL1-likeParasteatodatepidariorum             KMIYHCKF-GEFGVM-----EGQFTEPSGVAVNA-QNDIIVADTNN-HRIQIF-DKEGRFKFQFGECGK--------------------RDGQLLYPNRVAVVK-----TSGDIIVTERS
NCL1Ascarissuum                               KMIYHCKF-GEFGVM-----EGQFTEPSGVAVNA-QNDIVVADTNN-HRIQVF-DKEGRFKFQFGECGK--------------------RDGQLLYPNRVAVNR-----VTGDFIVTERS
NCL1Brugiamalayi                              KMIYHCKF-GEFGVM-----EGQFTEPSGVAVNA-QNDIIVADTNN-HRIQVF-DKEGRFKFQFGECGK--------------------RDGQLLYPNRVAVNR-----LTGDFIVTERS
NCL1Caenorhabditiselegans                     KMIYHCKF-GEFGVM-----EGQFTEPSGVAVNG-QGDIVVADTNN-HRIQVF-DKEGRFKFQFGECGK--------------------RDGQLLYPNRVAVNR-----TTGDFVVTERS
BratAscarissuum                               DLILRWHI-GGIGVG-----PGQFNSPHGFCLGC-DDEILVADTNN-HRIQIL-SKQGKMVMQFGIAGT--------------------DDGHLFYPKKVVALRPRASLPDGGYIVVDKG
NHL2Caenorhabditiselegans                     KVHSVFGT-SQQGST-----IRELHCPSGFCLSD-TDDILIADTNN-HRVVVC-GPPHP--WKIGRPGT--------------------DDGQLCFPRKVIALR----GEAVRYVVLDKG
Brat-likeApismellifera                        PMHIRCKF-GQLGPS-----KGQFSSPHGFCLSA-DEDIIVADTNN-HRIQIF-EKTGIFKFQFGVPGK--------------------EEGQLWYPRKVAVMR-----NSGKFVVCDRG
NCL1-likeMyzuspersicae                        SMDIVAKF-GQMGSG-----LGQFHSPHGFCLSP-TEDIVVADTHN-HRIQVF-EKDGTYKTEFGREGK--------------------EDGYLFYPRKVVFLN------HNQIVVCDRG
Brat-likePapiliomachaon                       PMHIRFKF-GQLGGG-----KGQFNSPHGFCLGN-DEDIIVADTNN-HRITVF-DKSGNHKFNFGVAGK--------------------EEGQLWYPRKVAVVR-----ATGKFVVCDRG
Brat-likeCapitellateleta                      AMQIRCKF-GQLGPG-----KSQFNSPHGFCLGM-DEDIVVADTNN-HRIQVF-EKSGEFKYQFGIPGK--------------------EEGQLWYPRKVAVMR-----HCGKYVVCDRG
Brat-likeParasteatodatepidariorum             TMQIRCKF-GQLGPG-----KSQFSSPHGFCLGM-DEEIIVADTNN-HRIQVF-EKTGDFKYQFGVAGK--------------------EEGQLWYPRKVAVIR-----SSGKFVICDRG
Mei-P26Drosophilamelanogaster                 PMQIRCKF-GSLGTA-----KGQFNSPHGFCLGV-DEEIIVADTNN-HRIEVF-DKMGALKFQFGVAGK--------------------EEGQLWYPRKVAVMH-----NNGKFVVCDRG
NHL1Ascarissuum                               KSKPRLVF-GKKGSK-----EGDLNWPRGVTAIC-GTEFAVCDSSN-HRVCIF-NTQGRLLRVFGKYGT--------------------GEGQLDSAAGICCN------RFRQLIVSDRY
NHL1isoformcBrugiamalayi                      KGKPKLVF-GKKGSK-----EGELNWPRGLAILG-GNEFAVCDSSN-HRVCIF-NTGGRLLKMFGKYGT--------------------GDAQLDSAAGICYS------RYKHLIVSDRY
NHL1Caenorhabditiselegans                     RGRPRAVF-GRKGAK-----DGELNWPRGICALS-GGLVATCDSSN-HRVCVF-DKDGKFVRQFGGYGA--------------------GAGQLDSAAGLASS-------KLRVIVSDRY
NHL1Capitellateleta                           KKNPVMKIGGERGSA-----EGQFKLPRGVAISPQDGTIVIADSSN-HRAQVF-DQHGQYVRTFGSYGD--------------------SDGEFDCLAGVAISQ-----SGDVIIADRY
NHL1isoformX2Apismellifera                    KRRQLFKF-GMRGSE-----PGCFTWPRGLAVGP-DNSIVVADSSN-HRVQVF-DCNGNFMKEFGSYGS--------------------GEGEFDCLAGVAVNR------IGQYIIADRY
NHL1-likeAplysiacalifornica                   KGTAVVRF-GDRGSE-----PKSFTWPRGVAVSPMDDHIFVSDSSN-HRVQVF-DNSGKYLKSFGTYGQ--------------------GNGEFDCLAGIAING------LGQIIIADRY
NHL1Parasteatodatepidariorum                  KGKSCFKF-GSRGTE-----HGCFTWPRGVSVGT-DNTIVVADSNN-HRVQVF-SASGKYIQEFGTYGS--------------------ADGEFDCLAGVTVNR------IGQFIVSDRY
NHL1isoformX1Myzuspersicae                    KRQLLFKI-GGRGSE-----PGYFTWPRGVAVGP-DNLIVVADSSN-HRIQVF-DNSGKIFKDFGSYGN--------------------SEGEFDCLAGVAVNR------IGQYIIADRY
NHL1isoformX1Papiliomachaon                   KRRLQFSV-GSRGSE-----PGYFTWPRGIAVGP-DNTMVVADSSN-HRVQVF-DMNGIFIKEFGQYGS--------------------GEGEFDCLAGVAVNR------IGQYIIADRY
AbbaDrosophilamelanogaster                    KRQQLFQL-GGRGSE-----PGSFTWPRGLAVGP-DNSIVVADSSN-HRVQVF-DSNGIFVKEFGEYGN--------------------GEGEFDCLAGVAVNR------IGQYIIADRY
NHL1isoformX1Parasteatodatepidariorum         KRRMLMKI-GSRGSD-----SSNFTWPRGVAVGP-DNSIVVADSSN-HRIQVF-DSIGKFSFEFGTYGS--------------------GDGEFDCPAGVSVNR------IGQFIVSDRY
WechCeratitiscapitata                         MLTPTISF-ATEGHD-----DGQVSRPWGLCVDK-FGNVYISDRRN-NRVQVF-SLEGMLKFKFGRKGV--------------------GNGEFDLPAGICVDM------DNRIIVVDKD
WechBombyxmori                                PGVPMFSF-GREGQD-----EGQVSRPWGLCVDK-EGNIIVADRRN-NRIQIF-NSRGEFKMMFGSKGT--------------------GSGEFDLPAGITTDT------YGRIIVIDKD
WechisoformACluniomarinus                     ALVPGLTF-CFDGHE-----DGEVSRPWGICVNK-NNEIIVADRRN-NRIQVF-FADGGFKFKFGVKGT--------------------GSGQFDLPAGVATDQ------NNRIIVVDKD
WechisoformX1Aedesaegypti                     AVGPSTTF-GFDGHE-----DGQVSRPWGIAVDK-DGHVLVADRRN-NRVQVF-YPDGTFKLKFGSKGT--------------------ANGQFDLPAGICTDG------QNRIIVVDKD
WechMuscadomestica                            SNALSLSF-ATEGHE-----DGQVSRPWGLCVDK-MGHILVSDRRN-NRVQVF-NPDGSLKFKFGRKGV--------------------GNGEFDLPAGICVDI------DNRIIVVDKD
WechDrosophilamelanogaster                    SNALSLSF-ATEGHE-----DGQVSRPWGLCVDK-MGHVLVSDRRN-NRVQVF-NPDGSLKFKFGRKGV--------------------GNGEFDLPAGICVDV------DNRIIVVDKD
TRIM71Daniorerio                              VGLPMASF-GGEGDG-----DGQLCRPWGICVDK-EGYVVVADRSN-NRVQIF-KPCGTFHHKFGTLGS--------------------RPGQFDRPAGVACDS------QRRIIVADKD
TRIM71isoformX1Apismellifera                  SVTPLRII-GNDGNP-----QDNLCRPWGIACDR-EGHIIIADRSN-NRIQIY-FQDGSFLRRFGTHGT--------------------APGQFDRPAGVAVDA------RRRIVVADKD
TRIM71Cionaintestinalis                       AGTPVLTF-GKEGSK-----DGELCRPWGICCDS-QGHLIVADRSN-NRLQIF-NYDGSFHHKFGTSGS--------------------RPGQFDRPAGVTVNK------QGQIVVADKD
TRIM71-likeParasteatodatepidariorum           VGKVLLSF-GGEGDS-----DGKLCRPWGVCTDK-SGNIIVADRSN-NRVQIF-NPDGTFKFKFGSPGQ--------------------RPGQFDRPAGVTTDH------LNRIIVADKD
TRIM71Capitellateleta                         VGSVSLQF-GVEGSA-----DGELCRPWGICTHR-DGYIVIADRSN-NRIQIF-TQEGKFHHKFGAAGS--------------------RNGQFDRPAGVACNA------QGQIIVADKD
TRIM71isoformXenopuslaevis                    IGLPTLSF-GGEGDH-----DGKLCRPWGVCVDR-EGYIVVADRSN-NRVQIF-KPCGTFHHKFGSLGS--------------------RPGQFDRPAGVACDS------SRRIVVADKD
TRIM71Homosapiens                             IGLPGLSF-GSEGDS-----DGKLCRPWGVSVDK-EGYIIVADRSN-NRIQVF-KPCGAFHHKFGTLGS--------------------RPGQFDRPAGVACDA------SRRIVVADKD
TRIM71Ornithorhynchusanatinus                 VGLPGLSF-GSEGDN-----DGKLCRPWGVSVDK-EGYIIVADRSN-NRIQVF-KPCGAFHHKFGTLGS--------------------RPGQFDRPAGVACDA------SRRIVVADKD
Lin41Caenorhabditiselegans                    AKGPILTF-GKEGSG-----DGELCRPWGICVDQ-RGRVIVADRSN-NRVQIF-DKDGNFISKFGTSGN--------------------RPGQFDRPAGITTNS------LNNIVVADKD
Lin41Ascarissuum                              RTGPVFSF-GSEGTA-----DGQVCRPWGICCDN-KGRILVADRSN-NRIQVF-DKDGKFLMKFGSPGV--------------------RSGQFDRPAGIAVNS------MNEIVVADKD
Lin41isoformfBrugiamalayi                     RTGPLFSF-GSEGSA-----DGQFCRPWGICCDN-KGRILVADRSN-NRIQIF-DKEGNFLHKFGSPGT--------------------RPGQFDRPAGIAVNS------MNEIVVADKD
NHL3Daniorerio                                EDELIYRV-GTRGRE-----KGEFTNLQGISASI-SGRVIVADSNN-QCIQVF-TNDGQFKARFGVRGR--------------------SPGQLQRPTGVAVDT------NGDVIVADYD
TRIM2Parasteatodatepidariorum                 EDDLILKI-GHRGRG-----RGDFSNPQGVCCTT-DGHIIIADSNN-QCVQVFDSTTGEFHLRFGSRGR--------------------APGEMQRPTGVTTLP------NGNYAVADYE
TRIM2Capitellateleta                          EDDLVLSV-GMKGRA-----KGEFTNPQGVCCYK--DRVLVADSNN-QCVQVF-TCGGDFKLRFGTRGR--------------------QVGQLQRPTGVAVTL------NGNYLVADYD
TRIM2isoformX1Daniorerio                      EDDLIFRI-GTKGRN-----KGEFTNLQGVAASS-VGKVLIADSNN-QCVQIF-SNDGQFKSRFGIRGR--------------------SPGQLQRPTGVAVHP------SGDIIIADYD
TRIM2isoformX1Ornithorhynchusanatinus         EDDLIFRV-GTKGRN-----KGEFTNLQGVAAST-NGKILIADSNN-QCVQIF-SNDGQFKSRFGIRGR--------------------SPGQLQRPTGVAVHP------SGDIIIADYD
TRIM2Musmusculus                              EDDLIFRV-GTKGRN-----KGEFTNLQGVAAST-SGKILIADSNN-QCVQIF-SNDGQFKSRFGIRGR--------------------SPGQLQRPTGVAVHP------SGDIIIADYD
TRIM2Homosapiens                              EDDLIFRV-GTKGRN-----KGEFTNLQGVAAST-NGKILIADSNN-QCVQIF-SNDGQFKSRFGIRGR--------------------SPGQLQRPTGVAVHP------SGDIIIADYD
```

```
TRIM2LhomeologXenopuslaevis                  EDDLIFRV-GTKGRN-----KGEFTNLQGVAAST-NGKILIADSNN-QCVQIF-SNDGQFKSRFGIRGR--------------------SPGQLQRPTGVAVHP------SGDIIIADYD
TRIM3ShomeologXenopuslaevis                  EDEMILRV-GSRGRD-----KGEFSNLQGISTSC-NNRIVVADSNN-QCIQVF-TNDGLFKLRFGVRGR--------------------SPGQLQRPTGVAVDM------NGDIVVADYD
TRIM3isoformX1Daniorerio                     EDELIFRV-GTRGRE-----RGEFSNLQGISTTS-SGRIVVADSNN-QCIQVF-SNDGQFKLKFGVRGR--------------------SPGQLQRPTGVAVDM------NGDIIVADYD
TRIM3Musmusculus                             EDELVFRV-GSRGRE-----KGEFTNLQGVSAAS-SGRIVVADSNN-QCIQVF-SNEGQFKFRFGVRGR--------------------SPGQLQRPTGVAVDT------NGDIIVADYD
TRIM3Homosapiens                             EDELVFRV-GSRGRE-----KGEFTNLQGVSAAS-SGRIVVADSNN-QCIQVF-SNEGQFKFRFGVRGR--------------------SPGQLQRPTGVAVDT------NGDIIVADYD
TRIM32Homosapiens                            QCLFLKKM-GAKGST-----PGMFNLPVSLYVTS-QGEVLVADRGN-YRIQVF-TRKGFLKEIRRSPSGIDSFV----------LSFLGADLPNLTPLSVAMNC------QGLIGVTDSY
TP0421Treponemapallidum                      HFSESESF-HAVRRG-----TVLFRRPLSLCALA-DGTFWMSAYGS-NELLRF-DVNGRVIARTRGPV-----------------------EGFDRPFDVIQTR------SGDLLVSEFA
BB0236Borreliaburgdorferi                    ELNFKKLV-KVASLDNSIYKRPHGYQITSLRADK-YGGYYAANFVG-NEILYF-DVNNNVNALVKDGF-----------------------SYLKSPYDVIEA-------NNLLYVTLYS
NHL3Caenorhabditiselegans                    AFRIDSFH-AYDWQM-----PVTFKYPTKCWWDEPTKRMFVLDSGN-KRVRVL-RDNGDVITDVRN------------------------DEFKSGTVGMVYLG------NEELVVLSWQ
PGal-1Caenorhabditiselegans                  VFEQDREL-IGLFNP-----SKEIGQVSGLAVNK-NGHIVAFHRSG-RVWDEK-SFNDHETFNKDLGVINNKTIAIIS--REKKVIDEFGAGLFYMPHGLTIDN------NGDYWVTDVG
Pal2Drosophilamelanogaster                   SVPTPVLV-ENWPTE-----QHSFGQVTAVAVDP-QGSPVVFHRAE-RYWDVN-TFNESNIYYLIEYGPIKENTIYVLDAKTGAIKSGWGSNMFYMPHGLTIDL------HGNYWITDVA
Pal1Drosophilamelanogaster                   LNNTYVYQ-NAWPAN-----NVKLGAVTAVSFDK-AGNVVIFHRVN-RVWGQT-TFDNRNQYQEKYRGPIRESTILALEPATGKVQYDWGKNFFYMPHGLTVDP------EDNVWLTDVA
PamRattusnorvegicus                          GDFHVEEE-LDWPGV-----YLLPGQVSGVALDS-KNNLVIFHRGD-HVWDGN-SFDSKFVYQQRGLGPIEEDTILVIDPNNAEILQSSGKNLFYLPHGLSIDT------DGNYWVTDVA
AMDHomosapiens                               GDFHMEEA-LDWPGV-----YLLPGQVSGVALDP-KNNLVIFHRGD-HVWDGN-SFDSKFVYQQIGLGPIEEDTILVIDPNNAAVLQSSGKNLFYLPHGLSIDK------DGNYWVTDVA
Pamn-1Caenorhabditiselegans                  PFGIVQEA-IHENLG-----GVKLGQVAGLAFNN-EQQLLVFQRAG-RVWDAS-TFDNYNILLDKKPIADPVILVISYSGNQTKLERKLGGGQFYLPHGIYVDK------DGFVYTTDVG
MT3624Mycobacteriumtuberculosis              TSRQAASP-TTPSPPSQVVVPIDLWNPDGVTVDL-ADAVYVADSGH-KRLLKL-PAGSNTPTTLPF-------------------------TDTIGPGGVAVNS------NRDVYVIDED
PKNDMycobacteriumtuberculosis                SGQTVLPF-TG---------IDFRLSPSGVAVDS-AGNVYVTSEGMYGRVVKL-ATGSTGTTVLPF-------------------------NGLYQPQGLAVDG------AGTVYVTDFN
NHLRC1Homosapiens                            SAPGALTC-HHTFGG-----WGTLVNPTGLALCPKTGRVVVVHDGR-RRVKIF-DSGGGCAHQFGEKGD--------------------AAQDIRYPVDVTITN------DCHVVVTDAG
TRIM56Homosapiens                            RPIFYCSF-PTRMPG-----DKRSPRITGLCPFG-PREILVADEQN-RALKRF-SLNGDYKGTVPVP-------------------------EGCSPCSVAAL---------QSAVAFSA

BratDrosophilamelanogaster                   P--THQIQIYN-QYGQ-FVRKFGAT-----------ILQHPRGVTVDNK--GRIIVVECK-VMRVII--FDQNG---NVLHKFGCSK------------HLE------------------
BratisoformX1Papiliomachaon                  P--THQIQIYN-QYGQ-FVRKFGAN-----------ILQHPRGVTVDNK--GRIVVVECK-VMRVII--FDQVG---NVLQKFGCSK------------HLE------------------
BratApismellifera                            P--THQIQIYN-QYGQ-FVRKFGAN-----------ILQHPRGVTVDSK--GRIVVVECK-VMRVII--FDQAG---IVLQKFGCSK------------HLE------------------
BratMyzuspersicae                            P--THQIQIYN-QYGQ-FVRKFGAN-----------ILQHPRGITVDNK--GRIVVVECK-VMRVII--FDQAG---NVLVKFGCSK------------HLE------------------
BratParasteatodatepidariorum                 P--THQIQIYN-QYGQ-FVRKFGAN-----------ILQHPRGVTVDAK--GLIIVAECK-VMRVII--FDQEG---NVMHKFGCSK------------HLE------------------
NCL1-likeParasteatodatepidariorum            P--THQIQIYN-QYGQ-FVRKFGAN-----------ILQHPRGVTVDIK--GRIIVVECK-VMRVII--FDQLG---NVLQKFGCSK------------HLE------------------
NCL1Ascarissuum                              P--THQIQIYN-QYGQ-FLRKFGAN-----------ILQHPRGVCVDNK--GRIIVIECK-VMRVII--FDMFG---NILQKFSCSR------------YLE------------------
NCL1Brugiamalayi                             P--THQIQIYN-QYGQ-FLRKFGAN-----------ILQHPRGVCVDSK--GRIIVIECK-VMRVII--FDMFG---NILQKFSCSR------------YLE------------------
NCL1Caenorhabditiselegans                    P--THQIQVYN-QYGQ-FLRKFGAN-----------ILQHPRGVCVDSK--GRIIVVECK-VMRVII--FDMFG---NILQKFSCSR------------YLE------------------
BratAscarissuum                              D-NKARLQLFS-KVGE-FIHKIVA-----------SYIEYVSALTVNDA--GHVVVFNST-SAMFVL--DVDQGPIAKILKWADCGK------------MLC------------------
NHL2Caenorhabditiselegans                    GDGKTRAQIFE-ARGE-FVKRLNMMA------LVPRGGIEVSAAAATPN--GQLLLVDTA-GFVYSI--DVDAP---RVTFWFDAST------------QLG------------------
Brat-likeApismellifera                       N-ERSRMQIFT-KSGH-FIKKIAI-----------RYIDIVAGLAVTSE--GHILAVDSV-SPTVFV--ISDTG---DLLRWFDCSE------------YMR------------------
NCL1-likeMyzuspersicae                       S-DRSRVQIFEYPTGK-FIKKIQV-----------QFIEIVAGVAVTAN--GHLALVDSV-HPTVFVLNVNEDN---GIMKWFDCAS------------YME------------------
Brat-likePapiliomachaon                      N-ERSRMQIFT-KNGH-FLKKIAV-----------RFIDIVAGLAVTAE--GLIVAVDSV-TPTVFI--LSEEG---DLMSWFDCSE------------CMR------------------
Brat-likeCapitellateleta                     N-ERSRMQIFT-KNGH-FIKKIAI-----------RYIDIVAGLAITEQGERNIVAVDSV-SPTVFC--IGETG---ELVKWFDCSD------------YMR------------------
Brat-likeParasteatodatepidariorum            S-ERSRMQIFT-KTGH-FLKKIAI-----------RYIDIVAGLAITSE--GRIVAVDSV-APTVFI--ISETG---DLLHWFDCSE------------YMR------------------
Mei-P26Drosophilamelanogaster                N-ERSRMQIFS-KCGH-FMRKIAI-----------RYIDIVAGLAVTAK--GHIVAVDSV-SPTVFV--ISEEG---ELVRWFDCSD------------YMR------------------
NHL1Ascarissuum                              N---HRIVIFD-QLGQ-FIKAFGGHG------PSSGRFNNPWGVCVDET--GIIYAVDKD-NHRVQV--FDSNG---QFVSKFGSMGP--------GPGQLH------------------
NHL1isoformcBrugiamalayi                     N---HRIMIFD-QDGH-CVRKFGGHG------PSNGRFNNPWGVAVDDM--GMIYVVDKD-NHRVQM--FDSKG---QFAGKFGTMGP--------GISQFH------------------
NHL1Caenorhabditiselegans                    N---HRISVFG-LEGD-HLFSFGGHG------QGNAKFNNPWGVAVDDL--GSIYVADKD-NHRVQV--FDKNG---QFIAKFGSFGH--------LPGQLN------------------
NHL1Capitellateleta                          N---HRIQIFD-SNFN-FKLKFGSEG------SSDGKFKYPWGVTVDHE--GLVYVCDKE-NHRVQV--FGADG---TFLRRFGSTGN--------QPGKLE------------------
NHL1isoformX2Apismellifera                   N---HRIQVLD-PSGR-FLRAFGSQG------TADGRFNYPWGITTDAL--GFIYVCDKE-NHRVQV--FQSDG---TFVGKFGSCGS--------GRGQLE------------------
NHL1-likeAplysiacalifornica                  N---HRVQVLD-RNGR-FQCSFGSEG------SSEGQLNYPWGVACDNM--GFIYVCDKE-NHRIQV--FQSNG---AFVRTFGHYGN--------RSGQFE------------------
NHL1Parasteatodatepidariorum                 N---HRLQVFD-PNGR-FIRAFGHEG------RSDGKFNYPWGVATDNL--GFIYVCDKE-NHRIQV--FQGDG---TYVTKFGGIGQ--------RPGELE------------------
NHL1isoformX1Myzuspersicae                   N---HRIQVLD-PSGR-FLRSFGSQG------SSDGRFNYPWGITTDAL--GFIYVCDKE-NHRIQV--FQSDG---TFVGKFGSNGN--------KIGQLE------------------
NHL1isoformX1Papiliomachaon                  N---HRIQVFD-PAGR-FLRSFGSQG------TGDGKFNYPWGITTDAL--GFIYVCDKE-NHRVQV--FQSDG---TFVGKFGSFGS--------KLGQLE------------------
AbbaDrosophilamelanogaster                   N---HRIQVLD-PQGR-FLRAFGSQG------TADGKFNYPWGVTTDAL--GFIYVCDKE-NHRVQV--FQSDG---SFVGKFGSCGR--------GEGQLE------------------
NHL1isoformX1Parasteatodatepidariorum        N---YRVQLFD-PTGR-FLRAFGCEG------RTDGRFNYPWGIATDSL--GFIYVCDKE-NHRVQV--FQSDG---TFVGKFGSLGS--------RPGQLE------------------
WechCeratitiscapitata                        N---HRVQIFT-ANGL-FLLKFGSYG------KEYGQFQYPWDVAVNSR--RQIVVTDSR-NHRIQQ--FDSEG---RFIRQVIFDNH-------GLNKGIA------------------
WechBombyxmori                               N---HRVQIFT-SSGN-FILKFGSFG------KECGQFQYPWDVAVNSL--GNIVVTDTR-NHRIQL--FTSDG---TYITKFVFEGANP----AKMLKGPT------------------
WechisoformACluniomarinus                    N---HRVQVFS-ASGA-FILKFGSYG------KELNQFMYPWACAVNSK--SQILVTDSR-NHRIQL--FSPDG---NFISRFSFDGVNH----SRYLKGLT------------------
WechisoformX1Aedesaegypti                    N---HRVQVFS-TNGL-FLLKFGSYG------KDCGQFQYPWDVAVNIK--GEILVTDSR-NHRIQL--FNSEG---QFISRFSFDGVNH----SRYLKGLT------------------
WechMuscadomestica                           N---HRVQIFT-STGA-FLLKFGSYG------KEYGQFQYPWDVAVNSR--RQIVVTDSR-NHRVQQ--FDSEG---RFIRQIVFDNHG-------SNKGIA------------------
WechDrosophilamelanogaster                   N---HRVQIFT-ASGV-FLLKFGSYG------KEYGQFQYPWDVAVNSR--RQIVVTDSR-NHRIQQ--FDSEG---RFIRQIVFDNHG-------QTKGIA------------------
TRIM71Daniorerio                             N---HRIQIFT-FDGQ-FLLKFGEKG------TKNGQFNYPWDVAVNFE--GKILVSDTR-NHRVQL--FGPDG---TFLNKYGFEGA--------LWKHFD------------------
TRIM71isoformApismellifera                   N---HRIQVLT-MEGL-FLLCFGEKG------CRAGQFNYPWDVAANSE--CQIVVSDTR-NHRVQL--FSPEG---VFLRKYGYESSP------NMWKHFD------------------
TRIM71Cionaintestinalis                      N---HRIQIFC-FDGT-FVGMFGERG------SKSGQFNYPWDVAVNSV--GMIAVTDTR-NHRIQL--FSPDG---TFLHKYGFEGA--------LWKHFD------------------
TRIM71-likeParasteatodatepidariorum          N---HRIQIFD-GDGN-FILKFGERG------SKNGQFNYPWDVAVNAE--GHILVSDTR-NHRLQL--FGSDG---SFINKYGFDGG--------LWKQFD------------------
TRIM71Capitellateleta                        N---HRIQIFA-FDGT-FLLKFGEKG------SKNGQFNYPWDVAVSHE--GRVLVSDTR-NHRVQL--FSSDG---QFINKYGFEGS--------LWKHFD------------------
TRIM71isoformXenopuslaevis                   N---HRVQIFT-FEGQ-FLLKFGEKG------TKNGQFNYPWDVAVNAE--GKILVSDTR-NHRVQL--FGPDG---TFLNKYGFEGA--------LWKHFD------------------
TRIM71Homosapiens                            N---HRIQIFT-FEGQ-FLLKFGEKG------TKNGQFNYPWDVAVNSE--GKILVSDTR-NHRIQL--FGPDG---VFLNKYGFEGA--------LWKHFD------------------
```

```
TRIM71Ornithorhynchusanatinus                N---HRIQIFT-FDGQ-FLLKFGEKG------TKNGQFNYPWDVAVNPE--GKILVSDTR-NHRIQL--FGPDG---VFLNKYGFEGA--------LWKHFD-----------------
Lin41Caenorhabditiselegans                   N---HRVQVFD-ENGM-FLLKFGDRG------RAVGYFNYPWGVATNSH--NAIAVSDTR-NHRVQI--FTPQG---QFVRKCGFDSAY-------FFKNLD-----------------
Lin41Ascarissuum                             N---HRIQVFN-EKGE-FLLKFGERG------RTPGMFNYPWGVAVNAY--NQIAVSDTR-NHRVQI--FSPQG---HYIRKCGFDSSL-------YYKNLD-----------------
Lin41isoformfBrugiamalayi                    N---HRVQVFS-ERGD-FLLKFGERG------RTPGLFNYPWGVAVNSF--NQIAVSDTR-NHRVQM--FSPQG---HFIRKFGFDSSL------YNYKNFD-----------------
NHL3Daniorerio                               N---RWISIFS-PDGK-FKNKIGAG-----------RLMGPKGVAVDRN--GHIIAVDNK-ACCVFI--FQSNG---KLVTKFGARGT--------SDRQFA-----------------
TRIM2Parasteatodatepidariorum                N---KWISIHD-PKGK-YVSRIGIG-----------KLLGPKGVTVNKN--GHIIVVDNK-GNNVLV--FRENG---KVLHKFGSRPS--------DPGRFT-----------------
TRIM2Capitellateleta                         N---KWVSVFN-PDGK-YLNKIGTG-----------KLLGPKGICVDHN--GHIIVVDNK-ASSVFV--FQSNG---KLLHKFGARGN--------EPHQFA-----------------
TRIM2isoformX1Daniorerio                     N---KWVSIFS-SDGK-FKSKIGSG-----------KLMGPKGVSVDRN--GHIIVVDNK-SCCVFI--FQPNG---KLVSKFGNRGN--------SDKQFA-----------------
TRIM2isoformX1Ornithorhynchusanatinus        N---KWVSIFS-SDGK-FKTKIGSG-----------KLMGPKGVSVDRN--GHIIVVDNK-ACCVFI--FQPNG---KIVTRFGSRGN--------GDRQFAGT---------------
TRIM2Musmusculus                             N---KWVSIFS-NDGK-FKTKIGSG-----------KLMGPKGVSVDRN--GHIIVVDNK-ACCVFI--FQPNG---KIVTRFGSRGN--------GDRQFA-----------------
TRIM2Homosapiens                             N---KWVSIFS-SDGK-FKTKIGSG-----------KLMGPKGVSVDRN--GHIIVVDNK-ACCVFI--FQPNG---KIVTRFGSRGN--------GDRQFA-----------------
TRIM2LhomeologXenopuslaevis                  N---KWVSIFS-ADGK-FKTKIGSG-----------KLMGPKGVSVDRN--GHIIVVDNK-ACCVFI--FQPNG---KIVTRFGSRGN--------GDKQFA-----------------
TRIM3ShomeologXenopuslaevis                  N---RWVSIFS-PEGK-FKNKIGVG-----------RLMGPKGVAVDRN--GHIIVVDNK-SCCIFI--FQSNG---KLVGRFGGRGP--------GDKQQPGA---------------
TRIM3isoformX1Daniorerio                     N---RWLSIFS-PDGK-FKNKIGAG-----------RLMGPKGVAVDKN--GHIITADNK-ACCVFI--FQSNG---KLVTKFGAKGT--------SERQFADKSAPNTPTEPKQSKSGP
TRIM3Musmusculus                             N---RWVSIFS-PEGK-FKTKIGAG-----------RLMGPKGVAVDRN--GHIIVVDNK-SCCVFT--FQPNG---KLVGRFGGRGA--------TDRHFA-----------------
TRIM3Homosapiens                             N---RWVSIFS-PEGK-FKTKIGAG-----------RLMGPKGVAVDRN--GHIIVVDNK-SCCVFT--FQPNG---KLVGRFGGRGA--------TDRHFA-----------------
TRIM32Homosapiens                            D---NSLKVYT-LDGH-CVACHRS------------QLSKPWGITALPS--GQFVVTDVE-GGKLWC--FTVDR---GSGVVKYSC-----------LCSAV----------------
TP0421Treponemapallidum                      S---DRICRLT-KEGR-FLKSFGGKG------RGVGQLIGPQFLATDRY--DNIYVTDFG-NARVAV--FAPDG---APLFHFGQKSA--------RFFGFS-----------------
BB0236Borreliaburgdorferi                    S---DEIGVYD-KVLGVKRKSIGNKG------TKDGELLAPQYMAIDKR--NYIYVSEWG-NKRVSK--FGLEG---DFILHFGSRTS--------GYKGLL-----------------
NHL3Caenorhabditiselegans                    R---KMLSKLN-TRGE-ILKAVQF-----------SEFYQPTDVAVDSR--GRFVIADK---TKIFI--FDSNF---KPLLSFPVRE-----------TKGA-----------------
PGal-1Caenorhabditiselegans                  S---HQVHKID-AKTQ-KIVMSLGEKMV--PGEDQAHFCKPTDVAVAKN--GHIFVADGYCNSRILK--FDAKG---NLMAQINAATEE------NQPSEFV----------------
Pal2Drosophilamelanogaster                   M---HQAFKFK-PFSN-KPLLTIGKRFR--PGSSVKHLCKPTSIAVATT--GEFFIADGYCNSRILK--FNAAG---KLLRTIPQPPE---------FLSLQ-----------------
Pal1Drosophilamelanogaster                   M---HQVFKFP-PRGG-DGKPALTLGDA-FQPGSGRKFCKPTSVAVLDN--GDFFVADGYCNARILK--YSRKG---ELILFWGQNTFSGISYDVAPQNFFA-----------------
PamRattusnorvegicus                          L---HQVFKLD-PHSK-EGPLLILGRSM-QPGSDQNHFCQPTDVAVEPST-GAVFVSDGYCNSRIVQ--FSPSG---KFVTQWGEESSGS----SPRPGQFS-----------------
AMDHomosapiens                               L---HQVFKLD-PNNK-EGPVLILGRSM-QPGSDQNHFCQPTDVAVDPGT-GAIYVSDGYCNSRIVQ--FSPSG---KFITQWGEESSGS----SPLPGQFT-----------------
Pamn-1Caenorhabditiselegans                  S---HTVAKWK-IEGN-ELKNIWTSGELLMPGSDQHHYCKPTGITRVED---QLYVTDGYCNSRVVV--LDLNG---KRIRQFGLPGE--------DAGQFN-----------------
MT3624Mycobacteriumtuberculosis              S---HHVLKLA-AGIE-PPVELPFG-----------SLGDAHGLAVDRS--DSVYVVDYD-NAKVLK--LPPGA---DTPTELPFV-------------GLD-----------------
PKNDMycobacteriumtuberculosis                N---RVVTLAA-GSNN-QTVLPFD------------GLNYPEGLAVDTQ--GAVYVADRG-NNRVVK--LAAGS---KTQTVLPFT-------------GLN-----------------
NHLRC1Homosapiens                            D---RSIKVFD-FFGQ-IKLVIGG------------QFSLPWGVETTPQ--NGIVVTDAE-AGSLHL--LDVDF---AEGVLRRTERL---------QAHLC-----------------
TRIM56Homosapiens                            S---ARLYLIN-PNGE-VQWRRALS-----------LSQASHAVAALPS--GDRVAVSVA--GHVEV--YNMEG---SLATRFIPGGK--------ASRGLR-----------------


BratDrosophilamelanogaster                   --FPNGVVVNDK-QEIFISDNR---------------AHCVKVFNYEGQ-YLRQIGGE----------GITNYPIGVGINSNGEILIADNHNN---FNLTIFTQDG-QLISALESK----
BratisoformX1Papiliomachaon                  --FPNGVVVNDK-QEIFISDNR---------------AHCVKVFNYEGI-YLRQIGGE----------GVTNYPIGVGINASGEILIADNHNN---FNLTIFTQEG-QLVSALESK----
BratApismellifera                            --FPNGVVVNDK-QEIFISDNR---------------AHCVKVFNYEGA-YLRQIGGE----------GITNYPIGVGINAVGEILIADNHNN---FNLTIFTQDG-QLVSALESK----
BratMyzuspersicae                            --FPNGVVVNDK-QEIFISDNR---------------AHCVKVFNYEGQ-YLRQIGSQ----------GITNYPIGVGINASGEILIADNHNN---FNLTIFTQEG-QLVSALESK----
BratParasteatodatepidariorum                 --FPNGVVVNDK-QEIFISDNR---------------AHCVKVFDYEGH-FLRQIGGE----------GLTNYPIGVCINTHGEILVADNHNN---FNITVFTQDG-QLVSASESK----
NCL1-likeParasteatodatepidariorum            --FPNGVVVNDK-QEIFISDNR---------------AHCVKVFNYDGV-FLRQIGGE----------GLTNYPIGVCINQNGEILVADNHNN---FNITIFTQDG-QLVNASESK----
NCL1Ascarissuum                              --FPNGVCTNDK-QEILISDNR---------------AHCIKVFNYDGQ-FARQIGGE----------GITNYPIGVGINSAGEVVVADNHNN---FNLTVFSQDG-TMLSALESK----
NCL1Brugiamalayi                             --FPNGVCTNHK-QEILISDNR---------------AHCIKVFNYDGQ-FVRQIGGE----------GITNYPIGVGINYAGDVVVADNHNN---FNLTVFSQDG-TMISALESK----
NCL1Caenorhabditiselegans                    --FPNGVCTNDK-NEILISDNR---------------AHCIKVFSYEGQ-YLRQIGGE----------GVTNYPIGVGINSLGEVVVADNHNN---FNLTVFSQDG-TMIGALESR----
BratAscarissuum                              --EASDVSVFE--GLYYVTDYK---------------QHCVVVLTIDGD-LVRRFGAF----------EHTPYPIGVDVSKTGDVLVADSHGNH--FHILVCSKTG-QRLQDFECT----
NHL2Caenorhabditiselegans                    --EASDVAMFD--NLIYITDFK---------------HHCVQVYTSEGK-FIRKMGEP----------SQTPYPIGIDVSKAGEVLVADTHGNH--LHVVVFSPEG-QHIHSFTHN----
Brat-likeApismellifera                       --EPSDIAISG--KEYFVCDFK---------------GHCVVVFNEEGK-FLRRIGCD----------NVTNFPNGIDISDAGDILIGDSHGNR--FHVAVFSRDG-SLISEFECP----
NCL1-likeMyzuspersicae                       --EPSDIIVRD--DDYYVCDFK---------------GHSIVVFNDEGV-CLRRIGGE----------YITSFPNGIDISENGEILVGDSHGNR--FHVAVFSHDG-THLTDLECP----
Brat-likePapiliomachaon                      --EPSDIAISG--KEFYVCDFK---------------GHCVVVFDDEGR-FLRRIGCE----------NVTNFPNGIDVSDAGDVLIGDSHGNK--FHVAVFSRDG-VLVTEFECP----
Brat-likeCapitellateleta                     --EPSDIAISG--KEYFVCDFK---------------GHCVVVFNEDGQ-FMRRIGCE----------NITNYPNGIDISDHGDVLVGDSHGNR--FHVAVFQSDG-SLIGEFECP----
Brat-likeParasteatodatepidariorum            --EPSDIAVSD--KEFYICDFK---------------GHCVIVFSEEGK-FLRKIGSE----------NVTNFPNGIDISDSGDVLVGDSHGNR--FHIVVFSRQGDTLLSEFECP----
Mei-P26Drosophilamelanogaster                --EPSDIAIRD--NDFYVCDFK---------------GHCVAVFQDDGT-FLYRIGNE----------KVTCFPNGIDISNAGDVLIGDSHGNR--FHVACYSREG-ALQSEFECP----
NHL1Ascarissuum                              --NPQFIAYHKQTQHLYVTDSS---------------NHRVCVFDHNGN-PVFQFGQEGF------HNGQLKFPRGIAVDDQGFIIVADSGN----NRVQIFYPDG-RFMHSFGTWGN--
NHL1isoformcBrugiamalayi                     --HPQFIAVNKRTHNIYVTDSS---------------NHRVCIFDHNGN-PIFQFGSEGF------QNGQMKFPRGIAVDDQGFIIVADSGN----NRVQIFYPNG-RYMHSFGTWGN--
NHL1Caenorhabditiselegans                    --SPLFIAVSRVTHHVYVSDSS---------------NHRISVFDPHGV-HLFSFGEEGF------HGGQFKFPRGIAIDSQENLIIADSGN----NRIQVFDAQG-QFVSSFGTWGG--
NHL1Capitellateleta                          --NPYYITVGHD-RNIYVSDTH---------------NNRIQVFTSQGG-FVNSFGGPGD------QDGKFKHPKGITVDHNGFMVVADSGN----HRIQVLRSDG-TFFAKFGTKGR--
NHL1isoformX2Apismellifera                   --HPHYIAVSNT-NRVIVSDGN---------------NHRIQIFDVNGR-VLTSFGSEGS------DEGQFKFPRGVAVDDQGYIVVADSGN----NRIQIFSPEG-AFLKSFGGWGS--
NHL1-likeAplysiacalifornica                  --NPHYIAVSPD-NKVYVSDSS---------------NHRIQVFSIYGD-FLFSFGICGT------LRGQMKFPRGLAIDHQGFVVVADSGN----NRIQIFRGDG-RFYTMFGSWGS--
NHL1Parasteatodatepidariorum                 --HPHYVAVSTT-NRVIVSDSN---------------NHRIQIYDVNGR-LLSTFGTEGS------EEGQFKYPRGVAVDDQGYLIVGDSGN----NRIQIFHPDG-TFLKAFGTWGS--
NHL1isoformX1Myzuspersicae                   --HPHYIAVSNT-NRVIVSDCN---------------NHRIQVFDVNGR-VISSFGTEGS------ENGQFKFPKGVAVDDQGYILVADSGN----NRIQIFHPDS-TFLRAFGCWGS--
NHL1isoformX1Papiliomachaon                  --HPHYIAVSST-NRVLVSDSN---------------NHRIQVFDVNGR-VLSSFGEEGS------EDGQFKFPRGVAVDDQGYIVVADSGN----NRIQIFHPDG-TFLRAFGSWGS--
AbbaDrosophilamelanogaster                   --HPHYIAVSNT-NRVIVSDSN---------------NHRIQIFDVNGK-VLSTVGGEGS------DDGQFKFPRGVAVDDQGYIFVADSGN----NRIQIFNPDG-SFLKTFGSWGS--
NHL1isoformX1Parasteatodatepidariorum        --HPHYIAVTNT-NRVVVTDTN---------------NHRVQIFDVNGR-SLTTFGSEGS------DEGHFKFPKGIAVDDQGYIIVSDSGN----NRIQIFLPDG-TFLRCFGTWGT--
WechCeratitiscapitata                        --SPRGVCYTPA-GNIIVSDFD---------------NHCLYLVDPDVNEVLSAKGHEGS------GAQEFNRPSGICCDDDGRIIVADSKN----QRIVIHSPAL-DYLWSIDIRPSIN
WechBombyxmori                               --TPRGVCFTTS-GNIIVSDFE---------------NHRLLMVDPTLTKVLHCVGREGS------GIGELNRPSGIVTDDDGRIVVADSKN----HRVLVXTSEL-RILWAVDLKSP--
```

```
WechisoformACluniomarinus                  --TPRGAAFNPQ-GNIIISDFE---------------NHRLILIDASMSKILATKGHEGS------ALHEFCRPSGICCDDDGRIVVADSKN----QRVLIFSPML-EFLWTVEIRPSAR
WechisoformX1Aedesaegypti                  --TPRGACFTPQ-GDVIISDFE---------------NHRLLLIDATLTKVLAAKGHEGT------AVHEFSRPSGICCDDDGRVIVADSKN----QRVLIFSPQL-EFLWAVEIRPSSN
WechMuscadomestica                         --SPRGVCYTPT-GNIIVSDFD---------------NHCLYLIDPDINDILSAKGHEGS------GFQEFNRPSGICCDDDGRIIVADSKN----QRIVVYSPTL-DYLWDIEIRPSLN
WechDrosophilamelanogaster                 --SPRGVCYTPT-GNIIVSDFD---------------NHCLYLIDPDINDILSVKGHEGS------GFHEFNRPSGLCCDDEGRIIVADSKN----QRILVFNQNL-DFMWDIEVRPSIN
TRIM71Daniorerio                           --SPRGVAFNQE-GHLVVTDFN---------------NHRLLVIRPDCQ-SARFLGSEGT------GNGQFLRPQGVAVDQEDRIIVADSRN----HRIQVFEPNG-NFLCKFGTHGN--
TRIM71isoformApismellifera                 --SPRGVAFNPD-GNIVTTDFN---------------NHRLVIIDSDFV-HARVLECESP-----GAPKQFLRPQGLVIDDEGNIIVADSRN----HRIQIFDSTG-TLQWRFGNYGK--
TRIM71Cionaintestinalis                    --SPRGVAFSHD--RIIVTDFN---------------NHRLVIIEPDLQ-SARYLGSEGN------QALQFLRPQGVAIDEEGNIVVADSRN----HRIQVFTPTG-GLIAVFGGASTT-
TRIM71-likeParasteatodatepidariorum        --SPRGVSFTSD-GHMVVTDFN---------------NHRILVVHPNFQ-SARFLGQEGS------NNGHFLRPQGVAVDPEGHIIVADSRN----HRIQIFHPNG-HFLCKFGTYGV--
TRIM71Capitellateleta                      --SPRGVCFNNE-GHMVVTDFN---------------NHRLLVIHPDFQ-SARFLGSEGS------ANGQFLRPQGVCVDQEGNIIVADSRN----HRIQVFQPNG-NFLCKFGTPGS--
TRIM71isoformXenopuslaevis                 --SPRGVAFSQD-GYLVVTDFN---------------NHRLLIIKPDCQ-SAHFLGTEGT------GNGQFLRPQGVAVDQEGRIIVADSRN----HRVQIFEPNG-SFLCKFGTQGS--
TRIM71Homosapiens                          --SPRGVAFNHE-GHLVVTDFN---------------NHRLLVIHPDCQ-SARFLGSEGT------GNGQFLRPQGVAVDQEGRIIVADSRN----HRVQMFESNG-SFLCKFGAQGS--
TRIM71Ornithorhynchusanatinus              --SPRGVTFNHE-GHLVVTDFN---------------NHRLLVIHPDCQ-SARFLGSEGT------GNGQFLRPQGVAVDQEGRIIVADSRN----HRVQIFESNG-SFLCKFGTQGG--
Lin41Caenorhabditiselegans                 --SPRGLCYLPD-GQLLITDFN---------------NHRLAVLSPRNMSEMKVYGSEGD------GDGMFVRPQGVVIDPEGHILVCDSRN----NRVQVFASDD-MRFIGSFG---LG
Lin41Ascarissuum                           --SPRGVCFLPD-GLLVITDFN---------------NHRLAVVSSRGPTEMKCFGSEGE------GDGCFCRPQGVTTDSEGHILVCDSRN----NRVQVLSAED-MHCVATFGGSVPS
Lin41isoformfBrugiamalayi                  --SPRGVCYLHD-GQLVITDFN---------------NHRLVMMSSRGTVDMKMYGGEGD------SEGSFCRPQGITTDNEGHILVCDSRN----NRIQVLNLDG-MQCVASFGGAGLG
NHL3Daniorerio                             --GPHFVAVNNK-NEIVVTDFH---------------NHSVKVYSADGE-FLFKFGSHGE------GNGQFNAPTGVAVDVNGNIIVADWGN----SRIQVFDSAG-SFLSYINTS----
TRIM2Parasteatodatepidariorum              --GPHYAAVNSK-NQIVVSDFH---------------GHCIKIFDAEGS-FLTSFGSNGE------GNGQFNAPTGVTIDDQDNIIVADWGN----CRIQVFDING-SFLSFINTF----
TRIM2Capitellateleta                       --GPHFCAINSR-NDIIVSDFH---------------NHCVKVYDCEGT-FITSFGANGE------GNGQFNAPTGVAVDKNDNILVADWGN----SRIQVFDSSG-SFLSYIHTA----
TRIM2isoformX1Daniorerio                   --GPHFAAVNQN-NEVIVTDFH---------------NHSVKVFSPEGE-FILKFGSNGE------GNGQFNAPTGVAVDANGNIIVADWGN----SRIQVFDSSG-SFLSYINTS----
TRIM2isoformX1Ornithorhynchusanatinus      LDGPHFAAVNSN-NEIIVTDFH---------------NHSVKVFNQEGE-FMLKFGSNGE------GNGQFNAPTGVAVDSNGNIIVADWGN----SRIQVFDGSG-SFLSYINTS----
TRIM2Musmusculus                           --GPHFAAVNSN-NEIIITDFH---------------NHSVKVFNQEGE-FMLKFGSNGE------GNGQFNAPTGVAVDSNGNIIVADWGN----SRIQVFDGSG-SFLSYINTS----
TRIM2Homosapiens                           --GPHFAAVNSN-NEIIITDFH---------------NHSVKVFNQEGE-FMLKFGSNGE------GNGQFNAPTGVAVDSNGNIIVADWGN----SRIQVFDGSG-SFLSYINTS----
TRIM2LhomeologXenopuslaevis                --GPHFAAVNSN-NEIIVTDFH---------------NHSVKVFNQDGE-FILKFGSNGE------GNGQFNAPTGVAVDSNGNIIVADWGN----SRIQVFDGSG-SFLSYINTS----
TRIM3SfhomeologXenopuslaevis               LDGPHFVAVNNK-NEIVVTDFH---------------NHSVKVYSADGE-FLFKFGSHGE------GNGQFNAPTGIAVDSNGNIIVADWGN----SRIQVFDSSG-SFLSYINTM----
TRIM3isoformX1Daniorerio                   AFSPHFVAINNK-NEIVVTDFH---------------NHSVKVYNADGE-FLFKFGSHGE------GNGQFNAPTGVAVDGNGNIIVADWGN----SRIQVFDSSG-SFLSYINTT----
TRIM3Musmusculus                           --GPHFVAVNNK-NEIVVTDFH---------------NHSVKVYSADGE-FLFKFGSHGE------GNGQFNAPTGVAVDSNGNIIVADWGN----SRIQVFDSSG-SFLSYINTS----
TRIM3Homosapiens                           --GPHFVAVNNK-NEIVVTDFH---------------NHSVKVYSADGE-FLFKFGSHGE------GNGQFNAPTGVAVDSNGNIIVADWGN----SRIQVFDSSG-SFLSYINTS----
TRIM32Homosapiens                          --RPKFVTCDAE-GTVYFTQGLGLNLENRQNEHHLEGGFSIGSVGPDGQ-LGRQISHFFS------ENEDFRCIAGMCVDARGDLIVADSSR----KEILHFPKGG-GYSVLIREG----
TP0421Treponemapallidum                    --APGGIAILD--ELVYVADAL---------------KGAIYVFDTAGN-YVRTLVAEGT----------LKHVESVRAW-NGRLLVSLPN------EVMVVDVGL-ASLHTIARL----
BB0236Borreliaburgdorferi                  --GPTGVTYLN--ENIYVADSL---------------RNTIEVFDTSGN-HLYSVFTS------------IEGIEGLSSDFVGNNVIVSSKD-----GVYKYSIAK-KTITKILKA----
NHL3Caenorhabditiselegans                  --EVKCVGVGLD-DDVIVGTT-----------------DELLLYDGAGK-MLRKLNVSPPGTVMPKVGGRFNITTVTCDVTTGQIVVIFTDKKMDRTNIGVISYKG-DFLYSIEPGP---
PGal-1Caenorhabditiselegans                --VPHSLSLIEDMNIVCVADRE---------------NQRVQCFSAGLS-EGDRTLPTGI-----PITSATDIGRVFAIREREHYLIGVTGN-SEDVEAQMFSIDM-QTGKTETF-----
Pal2Drosophilamelanogaster                 --VPHAITLLEHLDLLCIADRE---------------NMRVVCPKAGLI-SSHGEGEPAA------TIQEPDLGRVFGVASFGDIVFAVNGP-TSMLPVRGFTIDP-RSETIIGH-----
Pal1Drosophilamelanogaster                 --IPHALTLVPELQLLCAADRE---------------NGRVQCFLSSNG-TFHSQYHNQL-------IGDRLFSMAYTPAAGGQLVIVNG----PTAELGIHPEHY-NEVHGFVLSMRSK
PamRattusnorvegicus                        --VPHSLALVPHLDQLCVADRE---------------NGRIQCFKTDTK-EFVREIKHASF---GRNVFAISYIPGFLFAVNGKPYFGDQEP----VQGFVMNFSS-GEIIDVFKPV---
AMDHomosapiens                             --VPHSLALVPLLGQLCVADRE---------------NGRIQCFKTDTK-EFVREIKHSSF---GRNVFAISYIPGLLFAVNGKPHFGDQEP----VQGFVMNFSN-GEIIDIFKPV---
Pamn-1Caenorhabditiselegans                --LPHDIVSDSA-GRLLVTDRE---------------NGRVQHMTTQGH-VIEEFKSTMF-----TNIYSAASHEDYVFMVPGRPIMGHETE--GIAVFVGRSGTG-LIEYAFGPTTKGK
MT3624Mycobacteriumtuberculosis            --HPYDVAVDGA-GTVYVTDSG---------------HNRVVALTAGSA-TPVHLPFA-----------DLSFPAGVTVDRDDSVYVADLNN----NRVLKLAAGS-NAQSQLPFTG---
PKNDMycobacteriumtuberculosis              --DPDGVAVDNS-GNVYVTDTD---------------NNRVVKLEAESN-NQVVLPFT-----------DITAPWGIAVDEAGTVYVTEHNT----NQVVKLLAGS-TTSTVLPFTG---
NHLRC1Homosapiens                          --NPRGVAVSWLTGAIAVLEHPLALG-------TGVCSTRVKVFSSSMQ-LVGQVDTFGL----SLYFPSKITASAVTFDHQGNVIVADTSG----PAILCLGKPE-EFPVPKPMV----
TRIM56Homosapiens                          --ALVFLTTSPQ-GHFVGSDWQ---------------QNSVVICDGLGQ-VVGEYKGPGL---------HGCQPGSVSVDKKGYIFLTLREV----NKVVILDPKG-SLLGDFLTA----

BratDrosophilamelanogaster                 ----------------------------------------------VKHAQCFDVALMDDG-SVVLASKDY-----RLYIYRYV--------
BratisoformX1Papiliomachaon                ----------------------------------------------VKHAQCFDVALMDDG-SVVLASKDY-----RLYIYRYV--------
BratApismellifera                          ----------------------------------------------VKHAQCFDVALMDDG-SVVLASKDY-----RLYIYRYV--------
BratMyzuspersicae                          ----------------------------------------------VKHAQCFDVALMDDG-SVVLASKDY-----RLYIYRYI--------
BratParasteatodatepidariorum               ----------------------------------------------VKHAQCFDVALMDDG-SVILASKDY-----RLYIYRYM--------
NCL1-likeParasteatodatepidariorum          ----------------------------------------------VKHAQCFDVALMDDG-SVVLASKDY-----RLYIYRYM--------
NCL1Ascarissuum                            ----------------------------------------------VKHAQCFDVALVEDG-SVVLASKDY-----RLYLYRYAP-------
NCL1Brugiamalayi                           ----------------------------------------------VKHAQCFDVALVEDG-SVVLASKDY-----RLYLYRY---------
NCL1Caenorhabditiselegans                  ----------------------------------------------VKHAQCFDVALVDDG-SVVLASKDY-----RLYLYRFLP-------
BratAscarissuum                            ---------------------------------------------QLKVSRCVGLRITSEG-NIVSISKHNH----NVLMFNTLY-------
NHL2Caenorhabditiselegans                  ---------------------------------------------EFRLSRCVGLRIAKSG-HIVTLCKHNH----TLFVFKPLI-------
Brat-likeApismellifera                     ---------------------------------------------YVKVSRCCGLKITSEG-YIVTLAKNNH----HVLVLNTLY-------
NCL1-likeMyzuspersicae                     ---------------------------------------------YIKVSRCCGLKITSGG-HIVTLAKNNH----HVLVLNATV-------
Brat-likePapiliomachaon                    ---------------------------------------------YVKVSRCCGLKITSEG-YIVTLAKNNH----HVLVLNTLY-------
Brat-likeCapitellateleta                   ---------------------------------------------YVKVSRCCGLNVTSEG-HVVTLAKNNH----HVLVLNTLY-------
Brat-likeParasteatodatepidariorum          ---------------------------------------------YVKVSRCCGLKITSEG-YVVTLAKNNH----HVLVLNTLY-------
Mei-P26Drosophilamelanogaster              ---------------------------------------------HVKVSRCCGLKITSEG-YVVTLAKNNH----HVLVLNTLY-------
NHL1Ascarissuum                            --------------------------------------------GPGQLKGLEDITL-CDK-TIVVSDRENH----RIQLF-----------
NHL1isoformcBrugiamalayi                   --------------------------------------------APGQLKGVEAVAL-IDT-TIVVTDRENH----RIQLF-----------
```

```
NHL1Caenorhabditiselegans                  --------------------------------------------GAGQLKGVEDVCVTADG-SIVVTDRENH----RIQIF-----------
NHL1Capitellateleta                        --------------------------------------------GEGQLRDPEGIAITPDG-KILVADKDNY----RILMF-----------
NHL1isoformX2Apismellifera                 --------------------------------------------GDGEFKGLEGVAVTSTG-NIVVCDRENH----RVQVF-----------
NHL1-likeAplysiacalifornica                --------------------------------------------ENGHFKGLEGVAILANG-NIVVSDRENH----RIQIF-----------
NHL1Parasteatodatepidariorum               --------------------------------------------GDGEFKGLEGIAVSPTG-QILACDRENH----RIQVF-----------
NHL1isoformX1Myzuspersicae                 --------------------------------------------GDGEFKGLEGIAVMSNG-NILVCDRENH----RVQVF-----------
NHL1isoformX1Papiliomachaon                --------------------------------------------SDGEFKGLEGIAVMSGG-NIIVCDRENH----RVQVF-----------
AbbaDrosophilamelanogaster                 --------------------------------------------GDSEFKGLEGVAIMSNG-NILVCDRENH----RVQVF-----------
NHL1isoformX1Parasteatodatepidariorum      --------------------------------------------SDGEFKGLEGLAVTTGG-SIIACDRENH----RVQVF-----------
WechCeratitiscapitata                      R---------------------------------------LMPPTIDEKDRTCDVAVLPDG-RIVFLIELSP----DSKEGTSPYKRFVHIF
WechBombyxmori                             --------------------------------------------GLDDKDRPSDVALTPEG-YLVVLFETLP---DTARDVSSHGKQYIKVY
WechisoformACluniomarinus                  N---------------------------------------VLTSNMDEKDRPSDVALLNDG-RLVVMVETSP----DSREAISHNKTFVQVY
WechisoformX1Aedesaegypti                  N---------------------------------------LLTMGMDEKDRPSDVALLPDG-RLVVMVETSP----DARDQCSPQKTFIQIY
WechMuscadomestica                         P---------------------------------------LMPPTLDEKDRTCDVALMPDG-RIVFLIELSP----DSKEGSNPYKRFVHVF
WechDrosophilamelanogaster                 P---------------------------------------LMPPTLDEKDRTCDVAIMPDG-RIVFLIELSP----DSKEGSNPY-------
TRIM71Daniorerio                           --------------------------------------------GFGQMDRPSGIAVTPDG-VIVAVDFGNN----RILMF-----------
TRIM71isoformX1Apismellifera               --------------------------------------------DDNEMDRPSGIALCPDG-RIVIVDFGNN----RVLLI-----------
TRIM71Cionaintestinalis                    -------------------------------------------NGSALMDRPSGVTVTPHG-HIAVVDFGNN----RVIVF-----------
TRIM71-likeParasteatodatepidariorum        --------------------------------------------GPDQMDRPSGVCVSPEG-YIIIVDFGNN----RIQVF-----------
TRIM71Capitellateleta                      --------------------------------------------GVGQLDRPSGLCLSSDG-AILVVDFGNN----RVQIF-----------
TRIM71isoformX1Xenopuslaevis               --------------------------------------------GFGQLDRPSGIAVTPDG-TIVVVDFGNN----RILAF-----------
TRIM71Homosapiens                          --------------------------------------------GFGQMDRPSGIAITPDG-MIVVVDFGNN----RILVF-----------
TRIM71Ornithorhynchusanatinus              --------------------------------------------GFGQMDRPSGIAVTPDG-MIVVVDFGNN----RILIF-----------
Lin41Caenorhabditiselegans                 PVPNSGFQMP---QELPAPYSSLGGPFGAPAFSSAPTP--LTPSPRQLLDRPTDLAVGPDG-RIYVVDFGNN----CIRVF-----------
Lin41Ascarissuum                           ASCGGKMGI----------------------ASSTRSSPQSSGNDAVTLDRPTDICVSPEG-TIYVVDFGSN----CIRVY-----------
Lin41isoformfBrugiamalayi                  PTLASGVGNASGGESTITPLASMPATHTTVKTSSAQLPTSTNVPKHLVLDRPTDLCVSPDG-LIYVVDFGSS----CIRVY-----------
NHL3Daniorerio                             ---------------------------------------------ADPLYGPQGLALTSDG-HVVVADSGNH----CFKVYRYLQ-------
TRIM2Parasteatodatepidariorum              ---------------------------------------------GDPLYGPQGLAMTSDG-HVVVSDSGNH----CFKIYKYLQ-------
TRIM2Capitellateleta                       ---------------------------------------------GGPLYGPQGLDISPEG-QVIVADSGNH----CLKMYKYLQ-------
TRIM2isoformX1Daniorerio                   ---------------------------------------------ADPLYGPQGLALTSDG-HVVVADSGNH----CFKVYRYLQ-------
TRIM2isoformX1Ornithorhynchusanatinus      ---------------------------------------------ADPLYGPQGLALTSDG-HVVVADSGNH----CFKVYRYLQ-------
TRIM2Musmusculus                           ---------------------------------------------ADPLYGPQGLALTSDG-HVVVADSGNH----CFKVYRYLQ-------
TRIM2Homosapiens                           ---------------------------------------------ADPLYGPQGLALTSDG-HVVVADSGNH----CFKVYRYLQ-------
TRIM2LhomeologXenopuslaevis                ---------------------------------------------ADPLYGPQGLSLTSDG-HVVVADSGNH----CFKVYRYLQ-------
TRIM3ShomeologXenopuslaevis                ---------------------------------------------GDPLYGPQGLSLTSDG-HVVVADSGNH----CFKVYRYLQ-------
TRIM3isoformX1Daniorerio                   ---------------------------------------------ADPLYGPQGLALTSDG-HVAVADSGNH----CFKVYRYLQ-------
TRIM3Musmusculus                           ---------------------------------------------AEPLYGPQGLALTSDG-HVVVADAGNH----CFKAYRYLQ-------
TRIM3Homosapiens                           ---------------------------------------------AEPLYGPQGLALTSDG-HVVVADAGNH----CFKAYRYLQ-------
TRIM32Homosapiens                          ------------------------------------------------LTCPVGIALTPKG-QLLVLDCWDH----CIKIYSYHL-------
TP0421Treponemapallidum                    ---------------------------------------------GNAPIRLTAAVPDANG-NVLLADYKNE----RIEIVSRIS-------
BB0236Borreliaburgdorferi                  ---------------------------------------------DKMNSKISSSILDANN-QMIVSDFNNA----KVSVYKSDA-------
NHL3Caenorhabditiselegans                  ---------------------------------------------HEKFMAPCGIFVHKN--KAFVTDFERN----TVRSYKYK--------
PGal-1Caenorhabditiselegans                ---------------------------------------------AKGVRNTHALAIAADG-VMFVSQLEPS----RILEIRLL--------
Pal2Drosophilamelanogaster                 ---------------------------------------------WGEFKNPHSMAVSVNGSALYVTEIGTNHQTNRVWKYVLA--------
Pal1Drosophilamelanogaster                 QLV------------------------------------SKFGPNNLQFQNPHDVAVTADGNEIYVAELNPM----RIHKFVHRS-------
PamRattusnorvegicus                        ---------------------------------------------RKHFDMPHDIVASEDG-TVYIGDAHTN----TVWKFTLTE-------
AMDHomosapiens                             ---------------------------------------------RKHFDMPHDIVASEDG-TVYIGDAHTN----TVWKFTLTE-------
Pamn-1Caenorhabditiselegans                -----------------------------------------REQMGPQFGQPHCLRVCPDGGHIFVGDIAEG----KARLWQFKI-------
MT3624Mycobacteriumtuberculosis            ------------------------------------------------LFSPTDVAVDNDG-AVYVIDFYNR----MLKLPTA---------
PKNDMycobacteriumtuberculosis              ------------------------------------------------LNTPLAVAVDSDR-TVYVADRGND----RVVKLTS---------
NHLRC1Homosapiens                          ---------------------------------------------THGLSHPVALTFTKEN-SLLVLDTASH----SIKVYKVDW-------
TRIM56Homosapiens                          ---------------------------------------------YHGLEKP-RVTTMVDG-RYLVVSLSNG----TIHIFRVRS-------
```

Supplementary References

1. Aeschimann, F. *et al.* LIN41 Post-transcriptionally Silences mRNAs by Two Distinct and Position-Dependent Mechanisms. *Molecular cell* 65, 476-489 e474 (2017).
2. Tocchini, C. *et al.* The TRIM-NHL protein LIN-41 controls the onset of developmental plasticity in Caenorhabditis elegans. *PLoS Genet* 10, e1004533 (2014).
