# Supplementary material for: Evolutionary plasticity of the NHL domain underlies distinct solutions to RNA recognition
Source: Nat Commun. 2018 Apr 19;9:1549. doi: 10.1038/s41467-018-03920-7 (PMC5908797; doi:10.1038/s41467-018-03920-7)
Supplement: Supplementary file 3 — Description of Additional Supplementary Info [file 41467_2018_3920_MOESM3_ESM.pdf]

Description of Additional Supplementary Files

Title: Supplementary Dataset 1

Description: Plasmids. Plasmids used in this study are listed here. Sequences of primers and DNA fragments (gene blocks) used for cloning and cloning methods are mentioned for each plasmid.
