# Supplementary material for: Evolutionary plasticity of the NHL domain underlies distinct solutions to RNA recognition
Source: Nat Commun. 2018 Apr 19;9:1549. doi: 10.1038/s41467-018-03920-7 (PMC5908797; doi:10.1038/s41467-018-03920-7)
Supplement: Supplementary file 2 — Peer Review Report [file 41467_2018_3920_MOESM2_ESM.pdf]

Reviewers' comments:

Reviewer #1 (Remarks to the Author):

In this study, the authors have analyzed the RNA binding mechanisms of C.elegans and Zebrafish LIN41. By carefully analyzing publicly available data on RNA-RBP interactions using computational approaches, they found that the NHL proteins TRIM71, LIN41 and Wech might interact with a structured RNA element. Indeed, such RNA elements are present in the known CeLIN41 targets lin-29a and mab-10. Such interaction modes are also conserved in mammals where luciferase reporters were transfected into HEK cells. To further characterize the exact mechanisms of interaction between the NHL domain and RNA, the authors crystallized the NHL domain of Zebrafish LIN41 with and without its RNA motif. A positively charged pocket on the surface of the NHL domain accommodates a short loop of a hairpin comprising only three nucleotides. Again using computational analyses, the authors calculated a LIN41 response element (LRE). It contains the mentioned three nucleotide loop with an A/G at position III and an A-U pair next to the loop. Using RIP experiments, the authors find that this LRE is indeed found in target mRNAs preferentially in the 3' UTR. Finally, they compare their structural work with the structure of the related NHL domain of Drosophila BRAT and report that the two domains contact the RNA rather differently. Phylogenetic analysis further revealed that Zebrafish and CeLIN41 and Wech might follow similar mechanisms while the other NHL domains might be closer to BRAT suggesting evolutionary plasticity in this exciting domain.

This is a clear and well-written manuscript combining structural work, computational prediction and functional validation in different systems. It adds another facet to our understanding of the NHL domain. I have only a few minor points that could be considered.

1. The first paragraph of the results section is rather technical and difficult to understand for non-computational people. The authors may want to revisit this part.

2. Figure 1B: it seems that only the three analyzed proteins TRIM71, LIN41 and Wech interact with a structured RNA element. Is this rather unique to these few proteins or is there a bias in the data that has been used for the analysis? This should be clarified.

3. The authors should be more consistent with the use of gene names. They use TRIM71 or hsLIN41 for the same protein. In the last Figure, TRIM71 (DrLIN41) is used? 'Conservation vs TRIM71' means versus Human TRIM71? This needs to be made clearer.

Reviewer #2 (Remarks to the Author):

The manuscript by Kumari et al. describes a combined bioinformatics/computational, structural and functional study of proteins containing RNA-binding NHL domains. The authors carried out a meta-analysis of published RNAcompete data to delineate sequence and structure preferences of diverse RNA-binding domains. They observed three LIN41-related proteins from C. elegans, human and Drosophila as prominent outliers that recognized structure instead of sequence features. They filtered out RNA hairpins with tri-nucleotide loops as the preferred binding motifs and recognized that such features are present in untranslated regions of known C. elegans LIN41 target mRNAs. They confirmed binding of a LIN41 fragment containing filamin and NHL domains to these RNA ligands using EMSA and mutagenesis, and showed that these elements confer LIN41-mediated suppression on reporter constructs in vivo in C. elegans and human systems. The

authors then determined crystal structures of the zebrafish LIN41 filamin-NHL domains alone and in complex with RNA ligands, validating and expanding their expectations on the structure-based recognition of the bound RNAs. The observed binding mode is in stark contrast to RNA binding by the previously investigated NHL domain of Drosophila Brat that recognizes linear, single-stranded RNA sequences. The authors then conducted a comprehensive, structure-based computational analysis to work out a consensus for a LIN41 response element (LRE), featuring a tri-loop, a purine at position 3 of the loop and a loop-closing U-A base pair. They tested their LRE model using RNA binding experiments in vitro and RNA immunoprecipitation/sequencing using tagged LIN41 in adult worms. Finally, based on the observed diverse binding modes of NHL domain-containing proteins, the authors conducted a comprehensive phylogenetic analysis of this group of RNA-binding proteins, presenting evidence for the NHL domain having undergone rapid evolution to yield proteins with diverse RNA-recognition modes based on a common fold.

This manuscript is a nice example for the synergistic use of biochemical, structural, bioinformatics/computational and functional approaches to work out a comprehensive picture of the molecular mechanisms of RNA recognition, the biological roles and the evolutionary history of a widely distributed type of RNA-binding domain. The work described appears to be technically sound, the results are novel and interesting and they expand our understanding of the versatility of RNA-binding domains and their evolution. In particular, the results describe in detail one example, in which a RNA-binding protein recognizes distinct structural features of ligand RNAs rather than linear sequence. Clearly, the manuscript should be of interest to a large readership.

This reviewer has only some minor comments:

1. Why did zebrafish LIN41 not show up as an outlier in the initial meta-analysis, although it seems to behave similar to C. elegans and human LIN41? Were no data on this protein contained in the data sets? Then perhaps briefly mention?

2. In the Introduction or in the beginning of the Results, the authors should systematically introduce the domain organization of LIN41 proteins. Presently, elements like the “filamin” domain are not properly introduced.

3. At times in the main text, the authors should avoid specialist’s jargon and provide more generally understandable descriptions of their approaches (e.g. “n-mer dot-bracket strings”, “Z-value transformation”). Also, the reporter systems used should be briefly explained in the text to make the manuscript more readily accessible.

4. Likewise, in the description of the computational approach to delineate the LRE, what is, e.g. “pairing probability”, which features determine pairing probability and how is it computationally assessed?

54. P. 10, line 3: What is an “Ig-fold” axis?

6. While the manuscript is generally well written, the authors should again go through their text and carefully check meaning and grammar of phrases/sentences; e.g. “... almost all residues forming RNA binding are identical ...”

Reviewer #3 (Remarks to the Author):

Evolutionary Plasticity of the NHL Domain Underlies Distinct Solutions to RNA Recognition

In this manuscript the authors perform an interrogation of high-throughput RBP/RNA binding experiments to uncover a subset pf TRIM-NHL proteins as potential binders of structured RNA.

They find that LIN41 proteins prepress mRNA via structured RNA elements and that shape complementarity and electrostatic interaction underlie RNA specificity of LIN41. Lastly they claim that distinct mechanisms of RNA binding by LIN4 and Brat reflect evolutionary plasticity of the NHL domain

Critiques:

What was the range of RNA sizes that were analyzed in the meta-analysis? What were the size ranges of RNAs in each dataset included in the meta-analysis? Were the methods used similar enough that such a meta-analysis was appropriate?

Page 5, line 15: “folding the RNA sequences in silico.” Which RNA sequences did you fold for the 11-mer analysis? Did you fold only the bound fraction of RNAs from RNAcompete or did you fold all RNAs from the RNAcompete?

In Figure 1a, bottom, you show the top 10 7-mer sequences and 11-mer structure motifs. The ranking of the 7-mer sequences seems intuitive based on how they were bound in the RNAcompete experiment. However the 11-mer structural motif ranking is less clear to me because there are many different RNA sequences that can make any 1 particular structure. So was 1 structure actually an entire bin of different sequences that could form that structure? If so, did you sort the structures without regard to number of sequences that compose that structure?

The alternative way to do this analysis would be to sort the 11-mers based on how they bound in the RNAcompete then determine a structural motif later. In this case, the ranked motifs do not actually account for every possible RNA that can form that structure. Also, this means that you’re simply comparing 7-mer sequences to 11-mer sequences.

It appears that only reference 17 analyzed LIN-41 in their experiments so you could not compare results between datasets. So, how many, if any of the proteins analyzed were in more than 1 dataset that was included in the meta-analysis? And did the datasets have similar results for those particular proteins? It would be important to show similar results for the same protein otherwise the meta-analysis is flawed.

This analysis does not account for binding partners that may influence the RNAs bound.

Figure 2a seems to answer my question above whether structural motifs were sorted without regard to number of sequences composing that structure. Perhaps you should clarify this in the previous section.

Why did you stop the analysis at stem-loops with only 4 nucleotides in the loop?

Have you analyzed conservation of these proteins with their human homologs? In figure 7c where you’re looking at Conservation vs TRIM71 and Conservation vs Brat/
NCL1, it’s unclear to me which TRIM71 and Brat/NCL1 species you’re comparing.

We are very happy that our Reviewers found our work interesting and well done, and thank them for their efforts. Below is our point-by-point response to their comments.

REVIEWER 1

This is a clear and well-written manuscript combining structural work, computational prediction and functional validation in different systems. It adds another facet to our understanding of the NHL domain. I have only a few minor points that could be considered.

1. The first paragraph of the results section is rather technical and difficult to understand for non-computational people. The authors may want to revisit this part.

Following this suggestion, we have rephrased various sentences in this section to make it more understandable for general audience. See page 4.

2. Figure 1B: it seems that only the three analyzed proteins TRIM71, LIN41 and Wech interact with a structured RNA element. Is this rather unique to these few proteins or is there a bias in the data that has been used for the analysis? This should be clarified.

We were also surprised to see that so few proteins depend strongly on structural features of RNA to achieve binding specificity. We carefully designed our analysis to avoid any bias. However, as we mention in the text (page 4), we only scored for binding requiring specific sequence or structure features (high z-score), and not general features like GC-content or overall tendency to form structured RNA. It is also important to bear in mind that this experimental platform (RNAcompete) uses RNAs between 30-41 nucleotides long for the pull downs. Complex RNA structural motifs that may require longer RNA sequences would likely be missed by this platform. We now state this in the main text (page 4).

3. The authors should be more consistent with the use of gene names. They use TRIM71 or hsLIN41 for the same protein. In the last Figure, TRIM71 (DrLIN41) is used? 'Conservation vs TRIM71' means versus Human TRIM71? This needs to be made clearer.

To facilitate understanding, we use species-indicating prefixes (Hs, Ce, Dr; explained on page 2) followed by the generic LIN41. In Fig. 7, TRIM71 refers to a subfamily of proteins, which include homologous proteins from *Danio rerio*, *Apis mellifera*, *Ciona intestinalis*, *Parasteatoda tepidariorum*, *Capitella teleta*, *Xenopus laevis*, *Homo sapiens* and *Ornithorhynchus anatinus*. DrLIN41 in parentheses is shown as a representative of the TRIM71 subfamily as its crystal structure was solved. We have now clarified this in the figure legend. By 'Conservation vs TRIM71'

we mean the TRIM71 subfamily, which includes 8 TRIM71 proteins from various species as mentioned above. In addition to the figure legend, we have now re-labeled Fig. 7c accordingly.

REVIEWER 2

This manuscript is a nice example for the synergistic use of biochemical, structural, bioinformatics/computational and functional approaches to work out a comprehensive picture of the molecular mechanisms of RNA recognition, the biological roles and the evolutionary history of a widely distributed type of RNA-binding domain. The work described appears to be technically sound, the results are novel and interesting and they expand our understanding of the versatility of RNA-binding domains and their evolution. In particular, the results describe in detail one example, in which a RNA-binding protein recognizes distinct structural features of ligand RNAs rather than linear sequence. Clearly, the manuscript should be of interest to a large readership.

This reviewer has only some minor comments:

1. Why did zebrafish LIN41 not show up as an outlier in the initial meta-analysis, although it seems to behave similar to C. elegans and human LIN41? Were no data on this protein contained in the data sets? Then perhaps briefly mention?

Indeed, the zebrafish LIN41 has not been characterized by RNAcompete. We now mention in the text (on page 5) that other homologs have not been analyzed.

2. In the Introduction or in the beginning of the Results, the authors should systematically introduce the domain organization of LIN41 proteins. Presently, elements like the "filamin" domain are not properly introduced.

We have now explained the domain organization in the introduction, on page 2.

3. At times in the main text, the authors should avoid specialist's jargon and provide more generally understandable descriptions of their approaches (e.g. "n-mer dot-bracket strings", "Z-value transformation"). Also, the reporter systems used should be briefly explained in the text to make the manuscript more readily accessible.

We have rephrased various sentences and introduced additional explanation in the section describing computational analysis (page 4). We have now explained the reporter systems on page 6.

4. Likewise, in the description of the computational approach to delineate the LRE, what is, e.g. "pairing probability", which features determine pairing probability and how is it computationally assessed?

We have now added a short explanation (page 11) and also stated that this was calculated by using the (publicly available) tool RNAfold.

5. P. 10, line 3: What is an "Ig-fold" axis?

We mean the axis of the immunoglobulin fold of filamin domain. We now depict this axis in the revised Supplementary Fig. 2b.

6. While the manuscript is generally well written, the authors should again go through their text and carefully check meaning and grammar of phrases/sentences; e.g. "... almost all residues forming RNA binding are identical ..."

We have gone through the manuscript as suggested.

REVIEWER 3

What was the range of RNA sizes that were analyzed in the meta-analysis? What were the size ranges of RNAs in each dataset included in the meta-analysis? Were the methods used similar enough that such a meta-analysis was appropriate?

We believe the comparison is appropriate, as all experiments from the six studies were performed on the same platform (actually involving the same people), using the same pool of RNAs for the pull-downs (30-41 nucleotide long) and employing the same experimental approach. Also our *in silico* analysis was performed identically for all data sets.

Page 5, line 15: "folding the RNA sequences in silico." Which RNA sequences did you fold for the 11-mer analysis? Did you fold only the bound fraction of RNAs from RNAcompete or did you fold all RNAs from the RNAcompete?

We folded all RNA sequences from the RNAcompete experiments such that we could calculate the enrichment of any dot-bracket n-mer in the bound fraction over the unbound fraction. We now mention this in the main text (page 4).

In Figure 1a, bottom, you show the top 10 7-mer sequences and 11-mer structure motifs. The ranking of the 7-mer sequences seems intuitive based on how they were bound in the RNAcompete experiment. However the 11-mer structural motif ranking is less clear to me because there are many different RNA sequences that can make any 1 particular structure. So was 1 structure actually an entire bin of different sequences that could form that structure?

Yes, one particular dot-bracket n-mer could come from different sequences.

If so, did you sort the structures without regard to number of sequences that compose that structure?

Indeed, different structure 11-mers have different number of occurrences in the oligo pool. We are controlling for this by calculating enrichments for a particular structure 11 mer in the bound fraction as compared to the unbound fraction.

Generally speaking, a key requirement in our analysis was to identify sequence and structure motifs by an approach as similar as possible, in order to avoid any analytical bias favoring one over the other. We think we achieved this by a simple swap, replacing nucleotide n-mers by dot-bracket n-mers and performing the exact same analysis. This resulted in no other ambiguity between the sequence 7-mers and structure 11-mers apart from the length. As pointed out by the reviewer, different oligos (all of which are 30-41 nucleotide long) can contain the same structure 11-mer, but this is also true for sequence features (different oligos can contain the same sequence 7-mer).

The alternative way to do this analysis would be to sort the 11-mers based on how they bound in the RNAcompete then determine a structural motif later. In this case, the ranked motifs do not actually account for every possible RNA that can form that structure. Also, this means that you're simply comparing 7-mer sequences to 11-mer sequences.

Sorting by sequence 11-mers is not practical, given the limited number of oligos on the platform (n=241399). At an average oligo length of 35 nts, each oligo contains 25 11-mers (n=35-11+1). Therefore, in total there are 6034975 (241399*25) sequence 11-mer occurrences. The total number of combinations for 11-mers is 4194384 (4^11), which means that each 11-mer would occur on an average only in 1.4 oligos. Thus, the enrichment of every 11-mer would be based mostly on one single oligo, which would not be robust. This is the reason why we (and the authors

of this platform) analyzed 7-mer sequences. Thus, to make a fair comparison, we chose the dot-bracket n-mer length as 11 (producing 9020 combinations) to match the number of structural patterns to the 16384 (4^7) sequence patterns.

It appears that only reference 17 analyzed LIN-41 in their experiments so you could not compare results between datasets. So, how many, if any of the proteins analyzed were in more than 1 dataset that was included in the meta-analysis? And did the datasets have similar results for those particular proteins? It would be important to show similar results for the same protein otherwise the meta-analysis is flawed.

There are currently six, separately published, studies using this platform. As the purpose of these studies is to uncover novel RNA binding determinants, it is unlikely that one protein is examined more than once. Nevertheless, among the TRIM-NHL proteins, the *D. melanogaster* Brat was profiled by two different studies[1, 2]; we have now updated the Fig. 1b, having marked both Brat experiments. Although Brat was the only protein examined in different studies, there are also studies of two human proteins and their planarian homologs; one study examined human (Hs) BRUNOL6 and MBNL1[3] and another their planarian (Smed) counterparts, BRULI and MBNL-1 [4]. We have highlighted these pairs of proteins in a new Figure for the Reviewer 3, showing similar binding preferences for the same protein (Brat) or homologs (BRUNOL6/BRULI and MBNL1/MBNL-1). In the case of LIN41, though the binding of these proteins was examined in one study, we found it very suggestive that all the outliers are homologs.

This analysis does not account for binding partners that may influence the RNAs bound.

Indeed. The RNA compete experiments are performed *in vitro* with a single RNA binding protein/domain and will not account for any modulations to binding specificity arising from binding partners. Importantly, we show a correlation between the *in vitro* derived LIN41 binding motif and its association with RNAs *in vivo* (Fig. 6e). We have now explicitly added in the main text that RNAcompete experiments are performed *in vitro* to avoid any confusion (page 4).

Figure 2a seems to answer my question above whether structural motifs were sorted without regard to number of sequences composing that structure. Perhaps you should clarify this in the previous section.

We have rewritten this section now (page 4), as recommended also by the other two Reviewers.

Why did you stop the analysis at stem-loops with only 4 nucleotides in the loop?

The 4 nucleotides in a SL were meant as a control. We have now included the analysis for also 5 and 6 nucleotides in a SL to make this point stronger. Please see revised Fig. 2a and related text.

Have you analyzed conservation of these proteins with their human homologs? In figure 7c where you're looking at Conservation vs TRIM71 and Conservation vs Brat/NCL1, it's unclear to me which TRIM71 and Brat/NCL1 species you're comparing.

For each protein in question, we have collected their sequences from multiple species and compared their conservation against the TRIM71 and the Brat/NCL1 subfamilies. For clarity, we have now labeled Fig. 7c accordingly. The TRIM71 subfamily includes proteins from *Danio rerio*, *Apis mellifera*, *Ciona intestinalis*, *Parasteatoda tepidariorum*, *Capitella teleta*, *Xenopus laevis*, *Homo sapiens* and *Ornithorhynchus anatinus*. The Brat subfamily includes proteins from *Drosophila melanogaster*, *Papilio machaon*, *Apis mellifera*, *Myzus persicae* and *Parasteatoda tepidariorum*. The NCL1 subfamily includes proteins from *Parasteatoda tepidariorum*, *Ascaris suum*, *Brugia malayi* and *Caenorhabditis elegans*. These details are shown in the Supplementary Fig. 5.

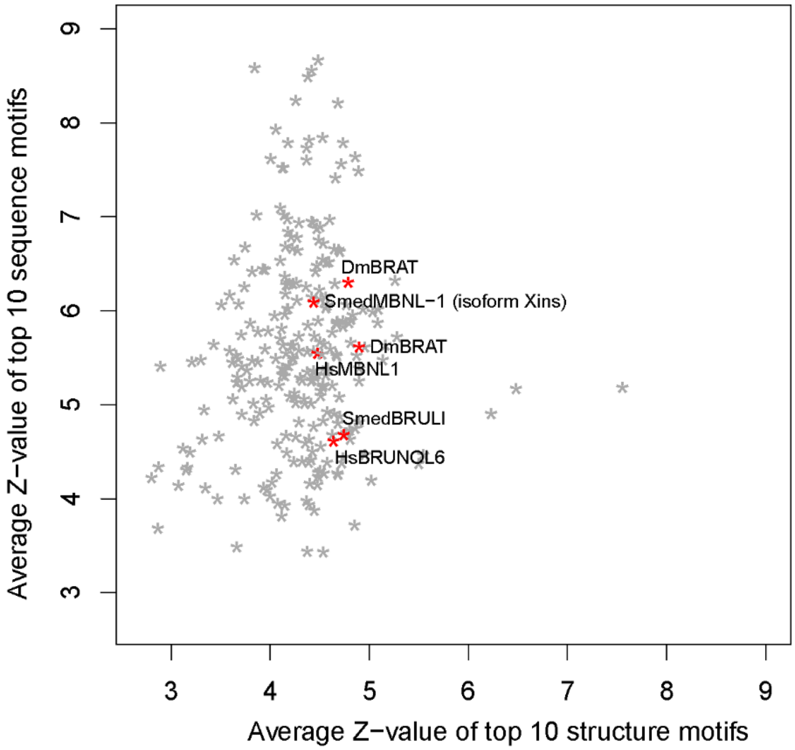

Figure for Reviewer 3: Sequence versus structure preference of proteins profiled by more than one study.

Average Z-values of the top 10 sequence motifs were plotted against the top 10 structure motifs, for each RNA binding experiment, comparing preference for sequence vs. structure. Proteins and their homologs that were profiled in two different studies are highlighted in red.

REFERENCES

1. Laver, J.D. *et al.* Brain tumor is a sequence-specific RNA-binding protein that directs maternal mRNA clearance during the Drosophila maternal-to-zygotic transition. *Genome biology* **16**, 94 (2015).
2. Loedige, I. *et al.* The Crystal Structure of the NHL Domain in Complex with RNA Reveals the Molecular Basis of Drosophila Brain-Tumor-Mediated Gene Regulation. *Cell reports* **13**, 1206-1220 (2015).
3. Ray, D. *et al.* A compendium of RNA-binding motifs for decoding gene regulation. *Nature* **499**, 172-177 (2013).
4. Solana, J. *et al.* Conserved functional antagonism of CELF and MBNL proteins controls stem cell-specific alternative splicing in planarians. *eLife* **5** (2016).

REVIEWERS' COMMENTS:

Reviewer #3 (Remarks to the Author):

All of my concerns have been addressed. The explanation of their meta-analysis is much clearer now.
